# Thieno-Thiazolostilbenes, Thienobenzo-Thiazoles, and Naphtho-Oxazoles: Computational Study and Cholinesterase Inhibitory Activity

**DOI:** 10.3390/molecules28093781

**Published:** 2023-04-27

**Authors:** Milena Mlakić, Ema Đurčević, Ilijana Odak, Danijela Barić, Ines Juričević, Ivana Šagud, Franko Burčul, Zlata Lasić, Željko Marinić, Irena Škorić

**Affiliations:** 1 Department of Organic Chemistry, Faculty of Chemical Engineering and Technology, University of Zagreb, Marulićev Trg 19, HR-10000 Zagreb, Croatia; mdragojev@fkit.hr (M.M.); edurcevic@fkit.hr (E.Đ.); ivana.sagud@halmed.hr (I.Š.); 2 Department of Chemistry, Faculty of Science and Education, University of Mostar, Matice Hrvatske bb, 88000 Mostar, Bosnia and Herzegovina; ilijana.odak@fpmoz.sum.ba (I.O.); ines.juricevic@fpmoz.sum.ba (I.J.); 3 Group for Computational Life Sciences, Division of Physical Chemistry, Ruđer Bošković Institute, Bijenička Cesta 54, HR-10000 Zagreb, Croatia; 4 Croatian Agency for Medicinal Products and Medical Devices, Ksaverska Cesta 4, HR-10000 Zagreb, Croatia; 5 Department of Analytical Chemistry, Faculty of Chemistry and Technology, University of Split, Ruđera Boškovića 35, HR-21000 Split, Croatia; franko@ktf-split.hr; 6 Teva Api Analytical R&D, Pliva, Prilaz Baruna Filipovića 25, HR-10000 Zagreb, Croatia; zlata.lasic01@pliva.com; 7 NMR Center, Rudjer Bošković Institute, Bijenička Cesta 54, HR-10000 Zagreb, Croatia

**Keywords:** cholinesterase inhibition, electronic structure, molecular docking, 1,3-oxazole, thiophene, 1,3-thiazole

## Abstract

Naphtho-triazoles and thienobenzo-triazoles have so far proven to be very potent inhibitors of the enzyme butyrylcholinesterase (BChE). Based on these results, in this work, new thienobenzo-thiazoles were designed and synthesized, and their potential inhibitory activity was tested and compared with their analogs, naphtho-oxazoles. The synthesis was carried out by photochemical cyclization of thieno-thiazolostilbenes obtained in the first reaction step. Several thienobenzo-thiazoles and naphtho-oxazoles have shown significant potential as BChE inhibitors, together with the phenolic thiazolostilbene being the most active of all tested compounds. These results are significant as BChE has been attracting growing attention due to its positive role in the treatment of Alzheimer’s disease. Computational examination based on the DFT approach enabled the characterization of the geometry and electronic structure of the studied molecules. Furthermore, the molecular docking study, accompanied by additional optimization of complexes ligand-active site, offered insight into the structure and stabilizing interactions in the complexes of studied molecules and BChE.

## 1. Introduction

The thiazole ring represents a very important motif with great potential in the field of medicine [1,2,3,4,5,6,7,8,9,10,11,12,13,14,15]. According to the literature, some stilbene analogs containing thiazole moiety were designed, showing promising topoisomerase I inhibitory activity [16]. Another group of thiazole-based stilbene analogs was also evaluated for DNA topoisomerase I.B. (Top1) inhibitory activity. Top1-mediated relaxation assays showed that the synthesized compounds possessed variable Top1 inhibitory activity [17]. Some analogs of benzimidazole-based thiazoles assessed for their in vitro acetylcholinesterase (AChE) and butyrylcholinesterase (BChE) inhibitory activity were found to exhibit good inhibitory potential against cholinesterase enzymes [18]. It was known that high BChE levels are interconnected with the distinctive neuropathologic characteristic of Alzheimer’s disease (AD) and that both enzymes BChE and AChE are pharmacologically appropriate targets in neurodegenerative disorders. On the other hand, benzothiazole scaffold possesses a great medicinal significance, as benzothiazoles and their heterocyclic derivatives represent an important class of compounds having a large spectrum of biological activities [19,20] with some detected role in the treatment of AD as well [21,22].

**Figure 1:**
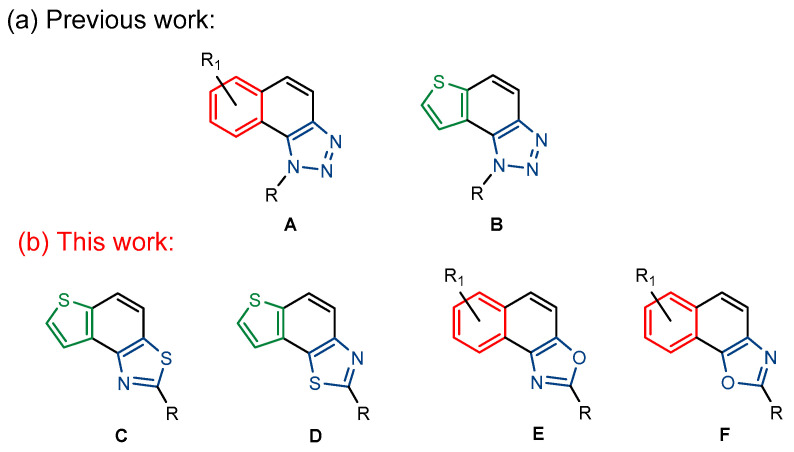
Previous (**a**) and current (**b**) structures of interest as cholinesterase inhibitors.

In our previous research, some new 1,2,3-triazolostilbene analogs were synthesized and photochemically transformed to substituted naphthotriazoles (Figure 1, structures A and B) for studying the acetyl- (AChE) and butyrylcholinesterase (BChE) inhibitory and anti-inflammatory activity. The naphthotriazoles as electrocyclization photoproducts showed interesting results on cholinesterase inhibition connected with the inhibition of TNFα cytokine production [23]. Among the expanded group of substituted thienobenzo/naphtho-triazoles [24], the most potent BChE inhibitor was the allyl-thienobenzotriazole, showing, at the same time, TNFα production inhibition in LPS-stimulated PBMCs, while *p*-methoxybenzyl- and hydroxybuthyl-naphthotriazoles with chloro or methoxy groups showed the best TNFα production inhibition. The latest study on the new functionalized group of thienobenzo/naphtho-triazoles [25] aimed to test their inhibition potency toward cholinesterases, where the compounds were obtained by two different, valuable synthetic strategies. Most of those synthesized and isolated structures also showed better inhibition of the BChE than AChE, and the binding affinity of BChE for seven compounds was similar to that reported for common cholinesterase inhibitors. These results are important as BChE has been attracting growing attention due to its important role in AD, as a complex neurodegenerative and irretrievable abnormality, especially in the advanced stage of the disease [26,27].

Motivated by all mentioned above, we continued with the synthesis and photochemistry of new 1,3-thiazolo-stilbenes (Figure 1, structures C and D), trying to see if the heterocyclic ring 1,3-thiazole versus 1,2,3-triazole (Figure 1, structure B) or the planar benzothiazole/naphthothiazole moiety in the photoproducts is crucial for the cholinesterase inhibitory activity of this group of compounds. Additionally, we also tested naphtho-oxazoles (Figure 1, structures E and F) obtained previously from 1,3-oxazole stilbenes [28] for inhibition of cholinesterase enzymes, given that they have a structural link with these new thiazole derivatives, but also with previously investigated naphtho-triazoles (Figure 1, structure A) [24]. A computational study of new molecules enabled more detailed insight into the characterization of their conformations and UV-Vis spectra. The molecular docking study is performed to identify stabilizing interactions responsible for the inhibitory activity of tested molecules.

## 2. Results and Discussion

### 2.1. Synthesis and Spectroscopic Characterization of New Thiazolostilbenes ***1a***–***10a*** and Thienobenzo-Thiazoles ***1***–***5***, ***7***, and ***9***

**Scheme 1:**
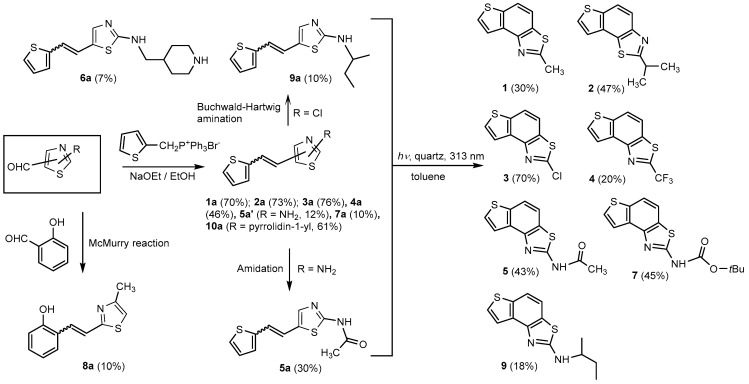
Reaction pathway for the synthesis of thieno-thiazolostilbenes **1a**–**10a** and thienobenzo-thiazoles **1**–**5, 7,** and **9**. Percentages in parentheses represent yields in reactions for compounds **1a**–**10a**, **7,** and **9**, and isolated yields for compounds **1**–**5**.

**Figure 2:**
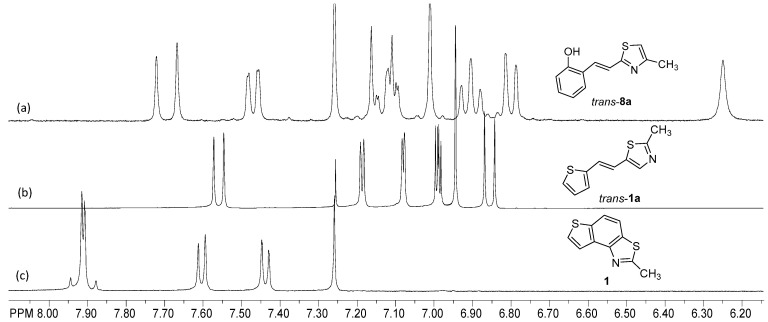
Crucial parts of the ^1^H NMR spectra of *trans*-**8a** (**a**), *trans*-**1a** (**b**), and **1** (**c**).

Thiazolo-stilbenes **1a**–**10a** were obtained as mixtures of *cis*- and *trans*-isomers (Scheme 1), some of them by Wittig reaction (**1a**–**4a, 5a′** (R = NH_2_) **7a** and **10a**) or alternative strategies. Thiazol-2-amines **6a** and **9a** were synthesized from the chlorine derivative **3a** by Buchwald-Hartwig amination, while thiazolo-stilbene **8a** was obtained by McMurry reaction. Yields on the products of Wittig reactions as a mixture of isomers were up to 76%. In some cases, pure isomers have been successfully separated (**1a**–**3a** and **10a**) and spectroscopically characterized (see Section 3 and ESI). Yields in the Buchwald-Hartwig amination reactions were 7% (**6a**) and 10% (**9a**), while the McMurry reaction was performed in 10% yield, where only the *trans*-isomer of compound **8a** was isolated. In the ^1^H NMR spectra of isolated geometrical isomers (Materials and Methods, Figure 2 and ESI), resolved patterns for ethylenic protons with the characteristic coupling constants, the signals for the protons on various substituents and the singlets for the protons on the 1,3-thiazole rings can be detected.

The further intention of the research was to transform the successfully obtained new thieno-thiazolostilbenes into their electrocyclization photoproducts thienobenzo-thiazoles (Scheme 1) as new biological targets. In aerobic conditions, a mixture of isomers of compounds **1a**–**10a** was dissolved in toluene (~2.5 × 10^−3^ M) and irradiated with 10 UV lamps at 313 nm in a quartz vessel with the addition of a catalytic amount of iodine for 1–5 h to achieve almost complete conversion. Starting compounds **1a**–**7a** proved reactive in obtaining photoproducts **1**–**5**, **7,** and **9** in isolated yields of 10–70%, depending on the substituents. Starting compounds **6a**, **8a** and **10a** were inactive in forming electrocyclization photoproducts. All isolated thienobenzo-thiazoles **1**–**7** were fully spectroscopically characterized (Materials and Methods, Figure 2 and ESI). In their ^1^H NMR spectra of **1a**–**10a**, the signals of ethylenic protons could be identified. The disappearance of the signals for ethylenic protons for the photoproducts **1**–**5**, **7**, and **9** compared to the starting thieno-thiazolostilbenes **1a**–**10a** was also easily detected. The photochemical reaction of **1a**–**10a** was generally accompanied by the appearance of some high-molecular-weight products, which remained on the chromatographic columns and were not investigated further.

**Figure 3:**
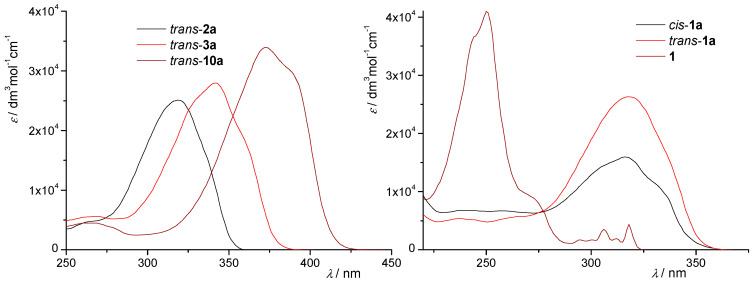
UV spectra (ACN) of thiazolostilbenes *trans*-**2a**, *trans*-**3a,** and *trans*-**10a** (**left**); and *cis*-**1a**, *trans*-**1a** and their electrocyclization product **1** (**right**).

UV-Vis spectra in acetonitrile (Figure 3) of representatives of *trans*-isomers are typical for diarylethenes [29,30] with strong absorption maxima between 318–373 nm (Figure 3 and Section 3). The values for the λ_max_ of the photoproducts are about 250 nm (Figure 3, right panel). The UV-Vis spectra of photoproducts are elaborated in more detail in Section 2.2., using the computational approach based on Time-dependent Density Functional Theory.

**Figure 4:**
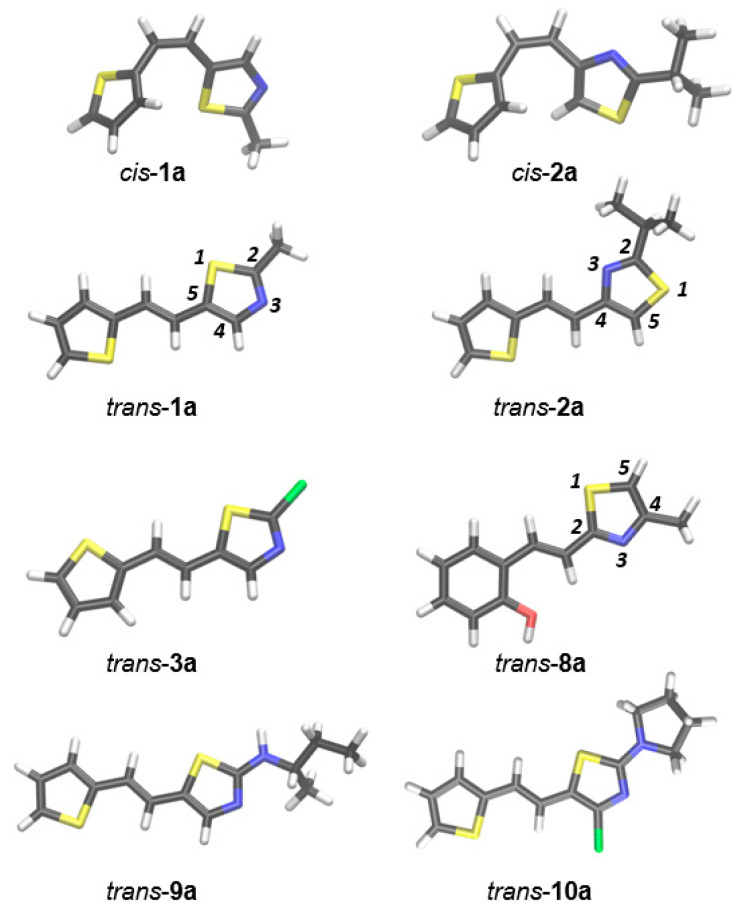
The most stable conformers of isolated *cis*- and *trans*-isomers of thieno-thiazolostilbenes. Geometries are optimized at M062X/6-31G(d) level of theory.

**Figure 5:**
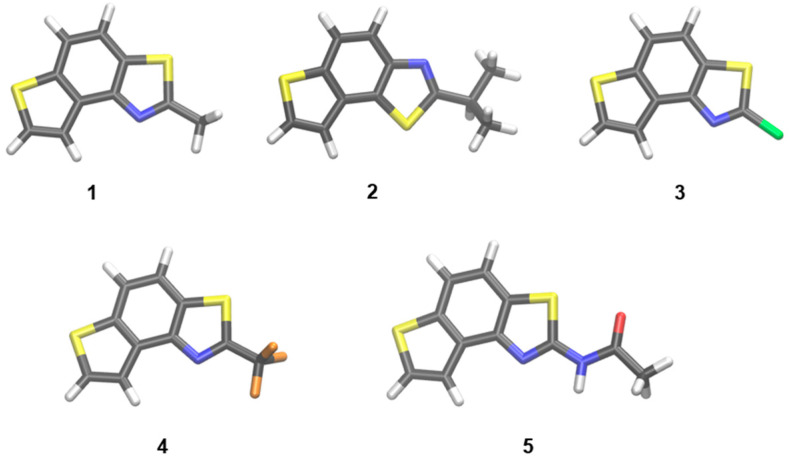
The geometries of isolated thienobenzo-thiazoles **1**–**5**, optimized at M062X/6-31G(d) level of theory.

We computationally examined conformations of isolated *cis*- and *trans*-isomers of thieno-thiazolostilbenes (*cis*-**1a**, *cis*-**2a**, *trans*: **1a**, **2a**, **3a**, **8a**, **9a,** and **10**a) and thienobenzo-thiazoles **1**–**5**, using M06-2X/6-31G(d) model. Optimized geometries of the most stable conformers are shown in Figure 4 and Figure 5.

All studied thieno-thiazolostilbenes have a common scaffold consisting of three subunits: the thiazole ring, central ethylene moiety, and thiophene, except for *trans*-**8a**, where thiophene is replaced with 2-hydroxyphenyl. It should be noted that in compounds **1a**, **3a**, **9a**, and **10a,** thiazole is substituted by 2-thiophene-2-yl-vinyl at position 5 (as marked in Figure 4), compound **2a** has 2-thiophene-2-yl-vinyl at position 4 of the thiazole, while in **8a** the substituent (possessing 2-hydroxyphenyl instead of thiophene) is placed at position 2. For all *trans*-isomers of thieno-thiazolostilbenes, the most stable conformation is the one where all three scaffold subunits are coplanar, which enables efficient conjugation of π-electrons. Not surprisingly, *cis*-isomers are not planar due to the steric hindrance caused by the vicinity of thiophene and thiazole in this configuration. Even in the most stable conformer of *cis*-**1a,** where one of the hydrogens from the thiophene is oriented toward thiazole’s sulfur, the planarity cannot be attained because this hydrogen bond is too weak to overcome the steric repulsions.

Optimized geometries of successfully isolated photocyclization products **1**–**5** are shown in Figure 5. Their structures are used to calculate UV-Vis spectra (see later). Expectedly, the orientation of the thiazole subunit in compound **2** is different than in other isolated thienobenzo-thiazoles due to the different position of 2-thiophene-2-yl-vinyl in starting compound **2a**.

UV-Vis spectra of synthesized and isolated compounds are measured in acetonitrile, as presented in Section 2.1. and Section 3. To reveal which orbitals participate in transitions observed in the measured spectra, we performed Time-dependent Density Functional Theory calculations (TD-DFT) for molecules whose spectra are shown in Figure 3 (*trans*-**2a**, *trans*-**3a**, *trans*-**10a**, *cis*-**1a**, *trans*-**1a**, and **1**) and remaining photocyclization products **2**–**5**. The time-dependent perturbation equation based on the Runge-Gross theorems [31] was solved for 20 excited singlet states for each of the molecules, using an implicit conductor-like polarizable continuum model (CPCM) for the description of solvent (acetonitrile). Results are presented in Table 1.

**Figure 6:**
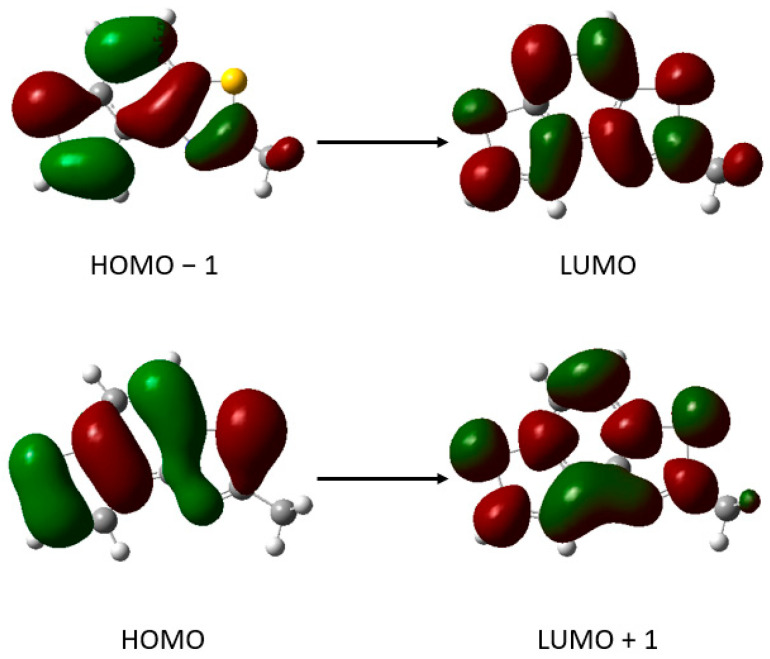
Canonical orbitals involved in main transitions in calculated spectra of compound **1**.

According to data from Table 1, for thieno-thiazolostilbenes, the calculated data are shifted toward higher values of λ_max_ for 8 to 15 nm, with one more considerable discrepancy of 29 nm found for the compound *trans*-**1a**. In spectra of photocyclization products **1**–**5**, the agreement between experimental and computational results is very good; positions of the absorption maximum in their calculated spectra differ for ~10 nm from the experimental data. The direction of this deviation is consistent, so we can assume that assignments of transitions predicted computationally are reliable. The transition responsible for absorption maximum in spectra of thieno-thiazolostilbenes is always HOMO to LUMO. In compound **1**, the transition between HOMO to LUMO + 1 is the most relevant, with a contribution of 73%. The second transition contributing to this absorption maximum with 27% is HOMO − 1 to LUMO. In the calculated spectrum of compound **2**, two major transitions corresponding to the transitions in the spectrum of compound **1** are found, with a contribution of 33% and 49%, respectively. However, two less significant additional transitions from the highest occupied orbital are also predicted: to LUMO (10%) and LUMO + 1 (8%). Figure 6 shows the orbitals that participate in transitions in the calculated spectra of compound 1. Just as the similarity found between experimental spectra of **1** and **2**, orbitals involved in the main transitions for compound **2** are analogous to orbitals of **1**.

**Figure 7:**
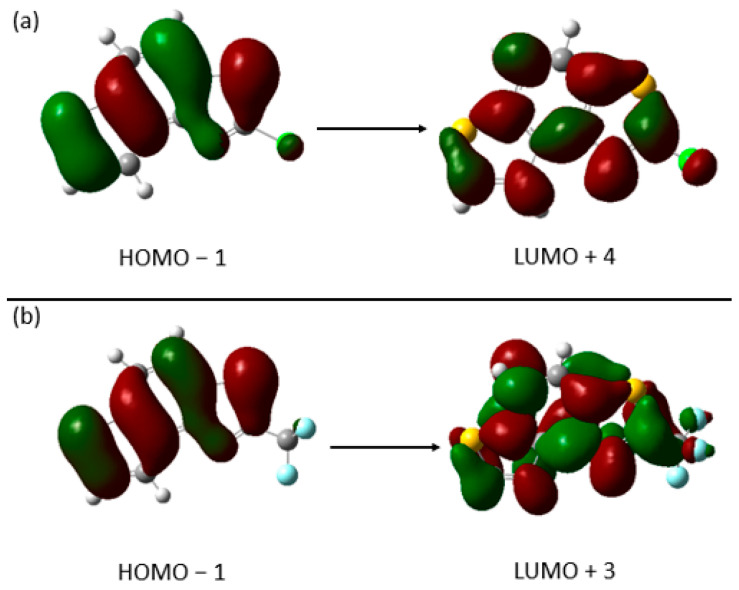
Canonical orbitals involved in main transitions in calculated spectra of compounds **3** (**a**) and **4** (**b**).

For compounds **3** and **4**, where the substituent at the thiazole ring is chlorine and -CF_3_ group, respectively, calculations predict that the dominant transition responsible for the position of absorption maximum involves very high virtual orbitals (LUMO + 4 and LUMO + 3). They are presented in Figure 7.

In the spectrum of compound **5**, the assignments of transitions responsible for absorption maximum reveal that the amide group at the thiazole is involved in the transitions, which was expected due to its co-planarity with the scaffold. 

### 2.2. Inhibitory Activity of Thiazolostilbenes, Thienobenzo-Thiazoles, and Naphtho-Oxazoles toward Enzymes Cholinesterases

All of the above-described pure derivatives synthesized in sufficient amounts were evaluated for cholinesterase inhibitory activity according to Ellman’s method (see Section 3). Tested compounds can be divided into three groups: thieno-thiazolostilbenes, thienobenzo-thiazoles (photocyclization products of thieno-thiazolostilbenes), and naphtho-oxazoles. Key determinants in a structure-activity relationship are a type of heterocyclic ring, the molecule’s geometry, and, where possible, the type and position of a substituent.

**Figure 8:**
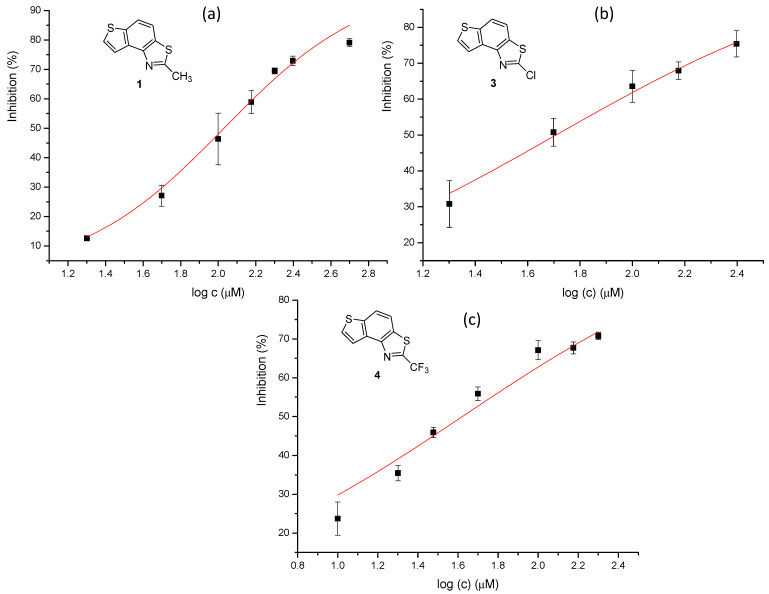
Dose–response curve for the inhibition of BChE by **1** (**a**), **3** (**b**), and **4** (**c**).

**Figure 9:**
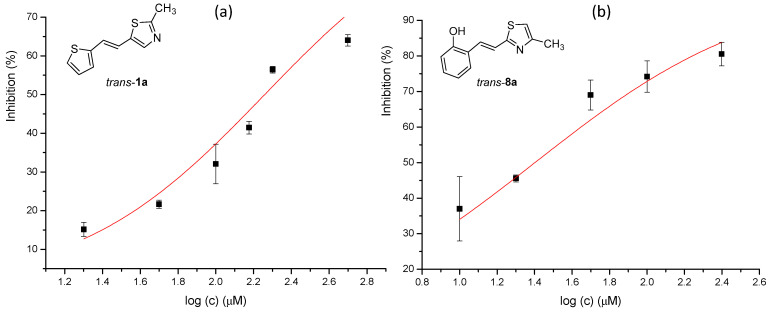
Dose–response curve for the inhibition of BChE by *trans*-**1a** (**a**) and *trans*-**8a** (**b**).

Tested compounds were active only toward BChE, and the results are presented in Table 2 and Figure 8 and Figure 9. Among stilbene derivatives, the best result is achieved by the derivative with a phenolic ring *trans*-**8a** and the thiophene derivative *trans*-**1a** (Table 2, Figure 9). In fact, the phenolic derivative is the most active of all tested thiazoles. Comparing its activity with previously studied thienostilbenes [32] shows that replacing thiophene with thiazole resulted in a total loss of activity toward AChE but only a slight decrease in inhibitory activity toward BChE. Replacement of phenol from *trans*-**8a** with thiophene in *trans*-**1a** reduces the IC_50_ value by seven times. Replacing the methyl group from *trans*-**1a** with an isopropyl group in *trans*-**2a** (along with the inversion of the sulfur and nitrogen position) also reduces the activity. Furthermore, a change in geometry from *trans*-**1a** to *cis*-**1a** also diminished inhibitory activity, as was observed earlier [24]. Other tested stilbene derivatives were ineffective inhibitors (*cis*-**1a**, *trans*-**3a** and **5a**). The limited solubility of compounds **9a** and *trans*-**10a** did not allow measurements at higher concentrations.

Three of the five tested electrocyclization products achieved an IC_50_ value, namely the one with methyl substituent **1**, chlorine **3,** and trifluoromethyl **4** (Table 2, Figure 8). Derivatives **3** and **4** have very good inhibitory activity with IC_50_ values in the range of huperzine A (IC_50_ = 53.6 μM), while methyl substituent reduces activity twice. Stilbene *trans*-**3a** and its photocyclizaton product **3** exemplify how planar naphthalene-like geometry is more active than the stilbene one, which was already observed in similar stilbene/naphthalene research [23,24,33]. Comparison of thiophene thiazoles with previously studied thiophene triazoles indicates that electrocyclic thiophene triazoles are much more successful inhibitors of both cholinesterases, BChE and AChE. It seems that the type of heterocyclic ring is crucial for the activity. 

Compounds **11**–**23,** possessing the oxazole group, also exhibited poor inhibitory activity towards AChE. The only compound with oxazole that showed some activity towards AChE is molecule **16** (with an IC_50_ value of 130.8 μM and 63.8% of inhibition at 201.7 as the concentration in μM for maximal effect measured). Naphtho-oxazole **15** showed the best inhibitory activity toward BChE. Comparing data for molecules **13**, **14**, and **15**, it is evident that the methyl group’s position affects the activity, with **14** having twice the activity of **13**, while compound **15**, having an extra methyl group on its five-membered ring, displayed three times higher inhibitory activity than **14**. In compound **16**, the nitro group placed in the same position as methyl in **14** only slightly improves the inhibitory activity. Substitution with methoxy and dimethylamino group in compounds **15** and **17,** respectively, causes the decrease in the inhibitory activity. Compound **19**, with inversed O and N atom positions in its five-membered ring, showed mildly higher activity when compared to analog naphtho-oxazole from the previous research [34]. Comparing compounds **20** and **22** to previously reported naphtho-oxazoles from the same study, slightly better inhibitory activity is also evident.

Comparing thienobenzo-thiazoles and naphtho-oxazoles studied here shows that these two types of compounds share significant similarities related to the presence of thiazole or oxazole ring, respectively. Both studied types of compounds are inactive toward the AChE; their inhibitory activity toward BChE depends on the substituent on the thiazole or oxazole ring, and the best results are obtained in the same range of concentrations. 

### 2.3. Molecular Docking

For compounds that showed the most promising inhibitory potential toward BChE, we performed a molecular docking study to visualize the possible conformations of compounds within the active site of BChE and identify stabilizing interactions between the active site of the enzyme and examined molecules. To assess and scrutinize the results suggested by molecular docking, we performed additional geometry optimizations of the active site docked with selected compounds, utilizing a quantum mechanical cluster-continuum approach [35]. For each investigated system, the structure with the lowest estimated free energy of binding (from the most populated cluster of distinctive conformations) was chosen and prepared for QM optimization. During the optimization, the α-carbon atoms of residues in the active site were held fixed, enabling the active site’s architecture in the enzyme to be preserved [35] (details in Section 3.6). Structures were optimized at the CPCM/B3LYP/6-31G(d) level of theory, with the dielectric constant ε = 4.

**Figure 10:**
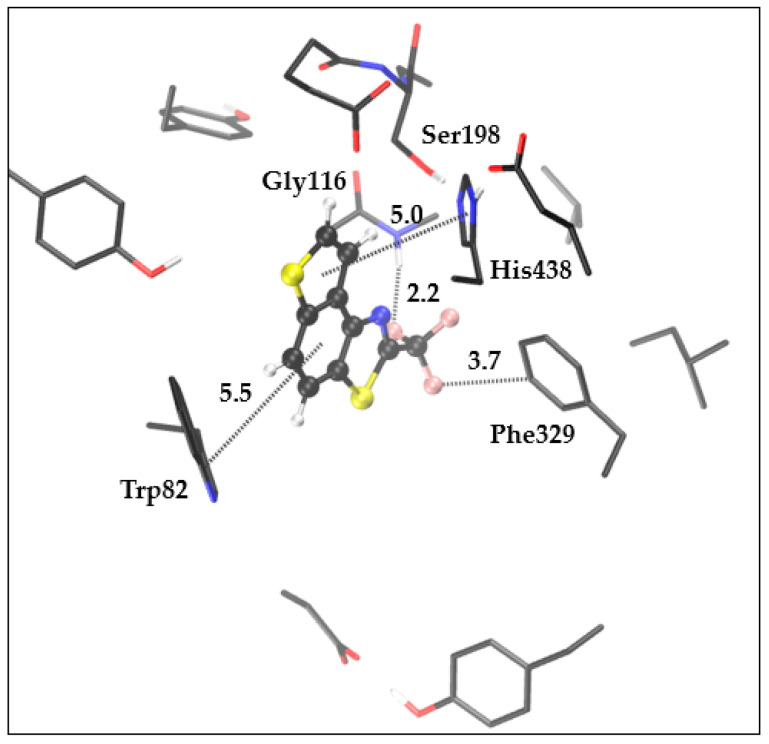
The optimized complex structure between the active site of BChE and molecule **4**. Distances are given in angstroms. Hydrogen atoms of residues are not shown for clarity.

Among thieno-thiazolostilbenes, compound **4** showed the best inhibitory activity toward BChE. The optimized structure of the complex formed between molecule **4** and the active site of BChE is presented in Figure 10.

The presence of the trifluoromethyl group enables its hydrophobic interaction with Phe329. At the same time, due to the fluorine’s ability to participate in polar interactions, the orientation of the trifluoromethyl group results in a hydrogen bond within another fluorine of -CF_3_ and hydrogen that belongs to peptide bond between Gly116-Gly117. This orientation of ligand also enables the involvement of the thiophene ring in π-π stacking with His438, while the central phenyl ring placement leads to the possibility for additional π-π stacking with Trp82 in the anionic subsite of the enzyme’s active site. It should be kept in mind that, given the shape and dimensions of ligand **4**, there are possibilities for different conformations of the complex that could induce alternative stabilizing interactions. 

**Figure 11:**
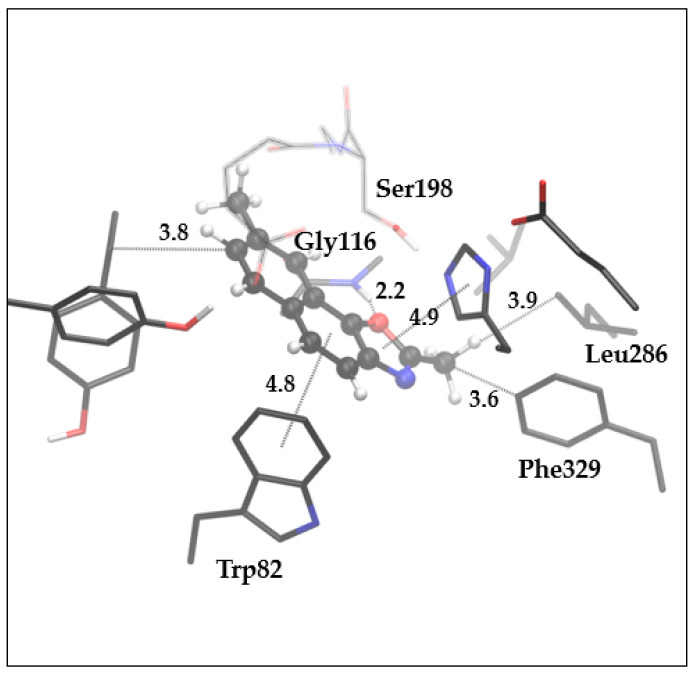
The optimized complex structure between the active site of BChE and molecule **15**. Distances are given in angstroms. Hydrogen atoms of residues are not shown for clarity.

Given the experimentally observed similarities between thieno-thiazolostilbenes and naphtho-oxazoles, we performed molecular docking of the best-performing oxazole, compound **15**, into the active site of BChE, followed by the optimization of the complex ligand-active site (Figure 11). 

Here, one hydrogen bond is observed between the oxazole’s oxygen and the amide group’s hydrogen that connects Gly116 and Gly117. The Methyl group at the oxazole ring is oriented toward the acyl pocket of the active site, thus enabling hydrophobic interaction with Leu296. The contacts with anionic site residues Phe329 and Trp82 result in hydrophobic interaction of aforementioned methyl and phenylalanine and π-π stacking between the central phenyl of the ligand and tryptophan. Another π-π stacking is present between the oxazole ring and His438. The plane of the main scaffold of the ligand is placed very close (~3 Å) to the esteratic part of the active site, thus blocking the substrate from approaching Ser198 and preventing the covalent bonding of the substrate to serine oxygen. 

Finally, the best inhibitory activity among all tested compounds was performed by thieno-thiazolostilbene *trans*-**8**. Unlike the compounds **4** and **15**, whose docking resulted in 25 poses so similar that they were grouped into one conformational cluster, structures obtained by molecular docking of *trans*-**8** resulted in five distinctive clusters of conformations, with the distribution of 11, 2, 3, 8, and 1 member per cluster (Table S1 in the Supplementary Materials). We optimized the structures of the enzyme-ligand complex formed with the lowest energy conformer from the cluster with 11 and 8 members, respectively.

**Figure 12:**
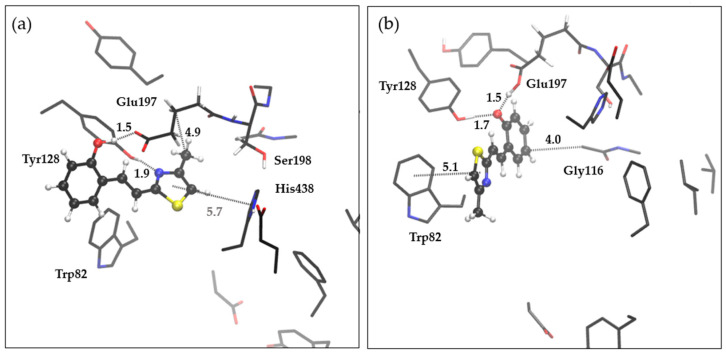
The optimized structures of the complex between *trans*-**8** and the active site of BChE. (**a**) The structure obtained by optimizing the docked compound’s geometry with the lowest binding energy from the most populated cluster, and (**b**) the optimized complex’ structure obtained from the starting geometry of docked compound with the lowest binding energy from the second most populated cluster. Distances given in angstroms. The ligand is presented with a ball and sticks model. Hydrogens of the residues omitted for clarity.

Figure 12 represents the optimized structures of the complex between *trans*-**8** and the active site of BChE. 

It is clear that thiazolostilbenes behave differently than thienobenzo-thiazoles and naphtho-oxazoles due to the flexibility caused by the presence of single bonds that can easily rotate. Additionally, *trans*-**8** possesses a hydroxy-group that can engage in a strong hydrogen bond with neighboring residues. In the first structure (Figure 12a), the HB is formed with Glu197, while the second structure (Figure 12b) reveals complete proton transfer from the hydroxyl group to Glu197, coupled with the occurrence of strong HB between Tyr128 and phenolate anion formed upon the proton transfer to glutamate. In structure (a), another H-bond is formed between Tyr128 and nitrogen of the thiazole ring. The orientation of methyl in the structure (a) enables hydrophobic alkyl-π interaction with a backbone that belongs to tripeptide Glu197-Ser198-Ala199 at a distance of 4.9 Å. In structure (b), the thiazole ring is involved in pi-pi stacking with Trp82, while in (a), the only possibility for this type of interaction could be realized with His438, but probably very weak because of the distance of 5.7 Å. Another hydrophobic interaction is observed in (b) between the phenyl ring and Gly116. In the end, both orientations of ligand *trans*-**8** possess enough potential for efficient stabilization within the active site of BChE. However, the structure (b) is slightly thermodynamically more stable (Δ*G* = 2.9 kcal mol^−1^, obtained as a difference in free energies of (a) and (b) calculated at (CPCM)B3LYP/6-31G(d)) due to the presence of two strong hydrogen bonds. 

It should be kept in mind that the static picture obtained by the molecular docking study does not offer a full characterization of our ligands’ behavior in the active site of BChE. To obtain a faithful description of systems that covers most possibilities of binding modes within the active site, molecular dynamics simulations should be applied.

## 3. Materials and Methods

### 3.1. General Remarks

Nuclear magnetic resonance (NMR) spectroscopic data for ^1^H and ^13^C nuclei were recorded at room temperature on Bruker Avance 300 and 600 MHz spectrometers. Deuterated chloroform, CDCl_3_, with tetramethylsilane as standard, was used for recording NMR spectra. Chemical shifts were reported in parts per million. All used solvents for the synthesis were purified by distillation and are commercially available. Anhydrous magnesium sulfate, MgSO_4_, was used for drying organic layers after extractions. The column chromatography was performed on columns with silica gel (60 Å, technical grade) using the appropriate solvent system. The abbreviations used in this experimental procedure were NMR—nuclear magnetic resonance, ACN—acetonitrile, EtOAc—ethyl acetate, PE—petroleum ether, E—diethylether, EtOH—ethanol, DCM—dichloromethane, and sh—shoulder. Preparative photochemical irradiations were carried out with 3.0 mL solutions in 1 mL closed vessel in a photochemical reactor Rayonet equipped with UV lamps of 313 nm. Before irradiation, in the reaction mixtures catalytic amount of iodine, I_2_ was added. High-resolution mass spectrometry (HRMS) analyses were carried out on a mass spectrometer (MALDI TOF/TOF analyzer) equipped with an Nd:YAG laser operating at 355 nm with a fitting rate of 200 Hz in the positive (H+) or negative (-H) ion reflector mode. All solvents were removed from the solutions by rotary evaporator under reduced pressure.

### 3.2. General Procedure for the Synthesis of the 2-Thiophene Phosphonium Salt

The 2-thiophene phosphonium salt was first synthesized in a three-necked flask (0.25 L) by preparing the corresponding 2-thiophene bromide (0.06 mol). Phosphorus tribromide, PBr_3_ (0.02 mol), was added dropwise to a cooled (0 °C) reaction flask solution of 2-thiophene methanol (0.06 mol) and anhydrous E (75 mL). The reaction mixture was stirred for one h, and methanol (0.015 L) and water (0.1 L) were added directly. The reaction mixture was extracted with E, dried under anhydrous MgSO_4,_ and filtrated. The organic layer was evaporated under pressure. Yellow oil of corresponding 2-thiophene bromide was dissolved in toluene (0.02 L), and triphenylphosphine (0.04 mol) was added and stirred for three days at room temperature. The reaction mixture was filtrated under reduced pressure, and the beige salt was dried under pressure in an exicator for 12 h. Dried phosphonium salt was used in all further experiments. 

### 3.3. General Procedure for the Synthesis of New Thiazolostilbenes ***1a**–**10a***

Compounds **1a**–**10a** were obtained as mixtures of *cis*- and *trans*-isomers. Products **1a**–**4a**, **5a′** (R = NH_2_), **7a** and **10a** were obtained using Wittig reaction. The reaction apparatus was purged with N_2_ for 15 min before adding the reactants. The reaction is carried out in a three-necked flask (100 mL) equipped with a chlorine-calcium tube and an N_2_ balloon connected. Phosphonium salt (5 mmol) was added to the 40 mL of EtOH, and the mixture was stirred with a magnetic stirrer. The solutions of sodium ethoxides (5 mmol, 1.1 eq of Na dissolved in 10 mL of absolute ethanol) were added in strictly anhydrous conditions under nitrogen dropwise. Corresponding aldehydes (5 mmol) were then added to the reaction mixtures, and the reaction mixtures were allowed to stir for 24 h at room temperature. The reaction mixtures were evaporated on a vacuum evaporator and dissolved in toluene. Mixtures were then extracted with toluene (3 × 15 mL). The organic layers were dried under anhydrous magnesium sulfate, MgSO_4_. Products **1a**–**4a**, **5a′**, **7a**, and **10a** were isolated by repeated column chromatography on silica gel using PE/E, PE/DCM, and E/EtOAc solvent systems. The first isomer to eluate is *trans*-isomer, and the *cis*-isomer is isolated in the last fractions. The spectroscopic characterization of new thiazolostilbenes is given below. 

*2-methyl-5-(2-(thiophen-2-yl)vinyl)thiazole* (**1a**) (70%). Column chromatography on silica gel using PE/E (30%) afforded 815 mg of a *cis*- and *trans*-isomer mixture. Repeated column chromatography on silica gel using PE/E (5%) afforded pure *trans*-isomer in the first fractions and *cis*-isomer in the last fractions.


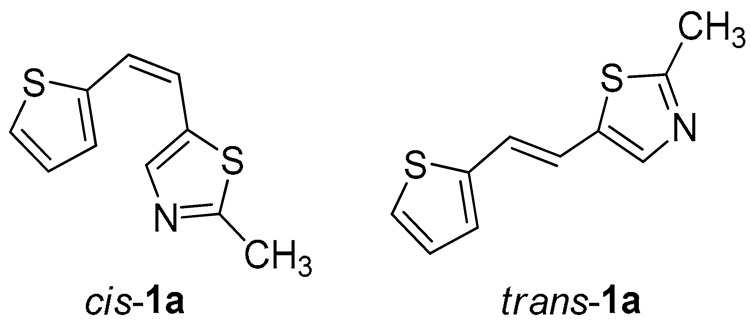


*(Z)-2-methyl-5-(2-(thiophen-2-yl)vinyl)thiazole* (*cis*-**1a**): 33 mg (isolated yield 20%), yellow oil, *R_f_*(PE/E = 5%) = 0.62; UV (ACN) *λ*_max_/nm (*ε*/dm^3^ mol^−1^ cm^−1^) 315 (15,948); ^1^H NMR (CDCl_3_, 600 MHz) *δ*/ppm: 7.25 (d, *J* = 7.7 Hz, 1H), 7.23 (d, *J* = 5.3 Hz, 1H), 7.13 (s, 1H), 6.97 (dd, *J* = 5.1, 3.6 Hz, 1H), 6.72 (d, *J* = 12.4 Hz, 1H), 6.43 (d, *J* = 12.4 Hz, 1H); ^13^C NMR (CDCl_3_, 75 MHz) *δ*/ppm: 165.1, 151.6, 139.6, 129.9, 126.7, 126.5, 124.0, 120.7, 117.1, and 19.1; and MS (ESI) *m*/*z* (%, fragment): 208 (100); 

*(E)-2-methyl-5-(2-(thiophen-2-yl)vinyl)thiazole* (*trans*-**1a**): 20 mg (isolated yield 10%), yellow oil, *R_f_*(PE/E = 5%) = 0.71; UV (ACN) *λ*_max_/nm (*ε*/dm^3^ mol^−1^ cm^−1^) 318 (26,303); ^1^H NMR (CDCl_3_, 600 MHz) *δ*/ppm: 7.55 (d, *J* = 15.8 Hz, 1H), 7.18 (d, *J* = 4.8 Hz, 1H), 7.08 (d, *J* = 3.5 Hz, 1H), 6.98 (dd, *J* = 5.1, 3.6 Hz, 1H), 6.94 (s, 1H), 6.85 (d, *J* = 15.8 Hz, 1H), 2.73 (s, 3H); ^13^C NMR (CDCl_3_, 75 MHz) *δ*/ppm: 166.2, 153.4, 142.5, 127.7, 126.6, 124.5, 124.2, 120.9, 114.7, and 19.4; and MS (ESI) *m*/*z* (%, fragment): 208 (100);

HRMS (*m*/*z*) for the mixture of configurational isomers C_10_H_9_NS_2_: [M + H]^+^calcd = 207.0176, [M + H]^+^measured = 207.0180.

*2-isopropyl-5-(2-(thiophen-2-yl)vinyl)thiazole* (**2a**) (73%). Column chromatography on silica gel using PE/E (20%) afforded 111 mg of a mixture of *cis*- and *trans*-isomer. Repeated column chromatography on silica gel using PE/E (10%) afforded pure *trans*-isomer in the first fractions and *cis*-isomer in the last fractions.


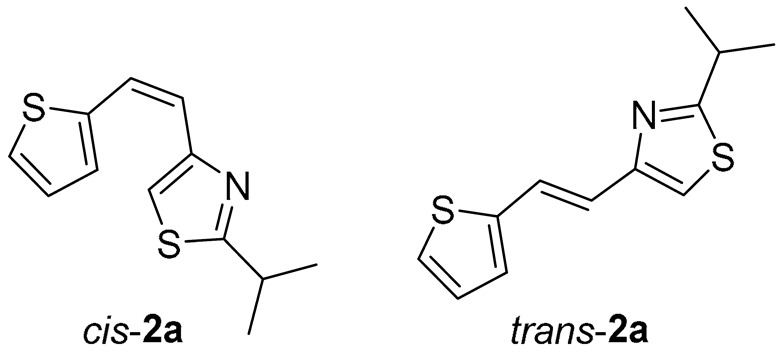


*(Z)-2-isopropyl-5-(2-(thiophen-2-yl)vinyl)thiazole* (*cis*-**2a**): 44 mg (isolated yield 40%), yellow oil, *R_f_*(PE/E = 10%) = 0.53; UV (ACN) *λ*_max_/nm (*ε*/dm^3^ mol^−1^ cm^−1^) 316 (13,832); ^1^H NMR (CDCl_3_, 600 MHz) *δ*/ppm: 7.29 (d, *J* = 3.4 Hz, 1H), 7.24 (d, *J* = 5.0 Hz, 1H), 7.13 (s, 1H), 6.96 (dd, *J* = 4.9, 3.5 Hz, 1H), 6.71 (d, *J* = 12.3 Hz, 1H), 6.42 (d, *J* = 12.3 Hz, 1H), 3.41–3.34 (m, 1H), 1.46 (s, 3H), 1.45 (s, 3H); ^13^C NMR (CDCl_3_, 75 MHz) *δ*/ppm: 177.1, 151.5, 139.7, 130.1, 126.8, 126.5, 123.7, 120.6, 116.4, 33.4, and 23.2; and MS (ESI) *m*/*z* (%, fragment): 236 (100); 

*(E)-2-isopropyl-5-(2-(thiophen-2-yl)vinyl)thiazole* (*trans*-**2a**): 27 mg (isolated yield 25%), yellow oil, *R_f_*(PE/E = 10%) = 0.56; UV (ACN) *λ*_max_/nm (*ε*/dm^3^ mol^−1^ cm^−1^) 319 (25,079); ^1^H NMR (CDCl_3_, 600 MHz) *δ*/ppm: 7.49 (d, *J* = 15.9 Hz, 1H), 7.11 (d, *J* = 5.0 Hz, 1H), 7.01 (d, *J* = 2.9 Hz, 1H), 6.93–6.91 (m, 2H), 6.80 (d, *J* = 15.9 Hz, 1H), 3.34–3.20 (m, 1H), 1.36 (s, 3H), 1.34 (s, 3H); ^13^C NMR (CDCl_3_, 75 MHz) *δ*/ppm: 177.2, 152.1, 141.6, 126.6, 125.4, 123.4, 123.1, 120.2, 112.8, 32.5, and 22.2; and MS (ESI) *m*/*z* (%, fragment): 236 (100); 

HRMS (*m*/*z*) for the mixture of configurational isomers C_12_H_13_NS_2_: [M + H]^+^calcd = 235.0489, [M + H]^+^measured = 235.0490.

*2-chloro-5-(2-(thiophen-2-yl)vinyl)thiazole* (**3a**) (76%). Column chromatography on silica gel using PE/E (5%) afforded 295 mg of a mixture of *cis*- and *trans*-isomer. Repeated column chromatography on silica gel using PE/E (1%) afforded pure *trans*-isomer in the first fractions.


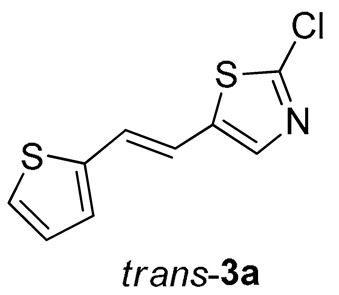


*(E)-2-chloro-5-(2-(thiophen-2-yl)vinyl)thiazole* (*trans*-**3a**): 20 mg (isolated yield 40%), yellow oil, *R_f_*(PE/E = 10%) = 0.59; UV (ACN) *λ*_max_/nm (*ε*/dm^3^ mol^−1^ cm^−1^) 342 (27,961); ^1^H NMR (CDCl_3_, 600 MHz) *δ*/ppm: 7.45 (s, 1H), 7.24 (d, *J* = 5.1 Hz, 1H), 7.07 (d, *J* = 3.3 Hz, 1H), 7.00 (dd, *J* = 4.9, 3.5 Hz, 1H), 6.92 (d, *J* = 16.2 Hz, 1H), 6.89 (d, *J* = 16.2 Hz, 1H; ^13^C NMR (CDCl_3_, 75 MHz) *δ*/ppm: 149.6, 141.2, 139.7, 139.1, 127.6, 127.3, 125.6, 125.3, and 117.0; and MS (ESI) *m*/*z* (%, fragment): 227/229 (100); HRMS (*m*/*z*) for C_9_H_7_ClNS_2_: [M + H]^+^calcd = 226.9630, [M + H]^+^measured = 226.9633.

*5-(2-(thiophen-2-yl)vinyl)-2-(trifluoromethyl)thiazole* (**4a**) (46%). Column chromatography on silica gel using PE/E (20%) afforded 66 mg of a mixture of *cis*- and *trans*-isomer of **4a** for the photochemical experiment. 


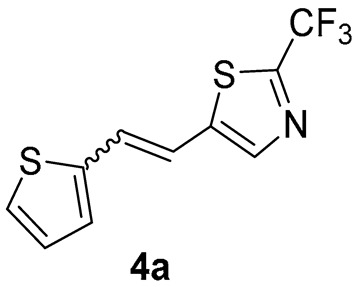


MS (ESI) *m*/*z* (%, fragment): 261 (100); HRMS (*m*/*z*) for the mixture of configurational isomers C_10_H_6_F_3_NS_2_: [M + H]^+^calcd = 260.9894, [M + H]^+^measured = 260.9896.

*N-(5-(2-(thiophen-2-yl)vinyl)thiazol-2-yl)acetamide* (**5a**) (30%). The reaction mixtures of *cis*- and *trans*-isomer were filtered under reduced pressure. Thiazol-2-acetamide **5a** was synthesized from thiazol-2-amine **5a′** in a round flask (0.025 L) using acetic anhydride (0.0015 L) at room temperature overnight. The water was added (0.00075 L), the reaction mixture was filtered under reduced pressure, and the beige solid was dried under pressure in an exicator for 2 h.


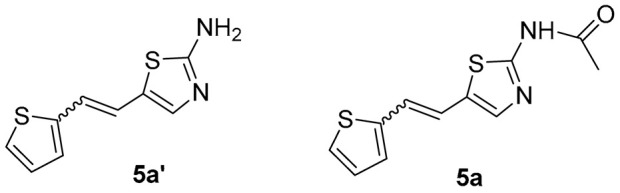


MS (ESI) *m*/*z* (%, fragment): 209 (100); HRMS (*m*/*z*) for the mixture of configurational isomers of **5a′**, C_9_H_8_N_2_S_2_: [M + H]^+^calcd = 208.0129, and [M + H]^+^measured = 208.0133.

MS (ESI) *m*/*z* (%, fragment): 251 (100), 209 (15), 121 (25); HRMS (*m*/*z*) for the mixture of configurational isomers of **5a**, C_11_H_10_N_2_OS_2_: [M + H]^+^calcd = 250.0234, and [M + H]^+^measured = 250.0235.

*N-(4-bromo-5-(2-(thiophen-2-yl)vinyl)thiazol-2-yl)pivalamide* (**7a**) (10%). Column chromatography on silica gel using PE/E (30%) afforded 30 mg of a mixture of *cis*-, *trans*-isomers, and starting aldehyde. 


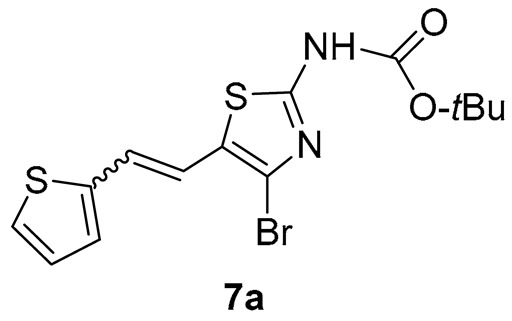


MS (ESI) *m*/*z* (%, fragment): 408/410 (20), 386/388 (10), 330/332 (100), 121 (55); HRMS (*m*/*z*) for the mixture of configurational isomers of **7a**, C_14_H_15_BrN_2_O_2_S_2_: [M + H]^+^calcd = 385.9758, and [M + H]^+^measured = 385.9751.

*4-chloro-2-(pyrrolidin-1-yl)-5-(2-(thiophen-2-yl)vinyl)thiazole* (**10a**) (61%). Column chromatography on silica gel using PE/E (20%) afforded 61 mg of a *cis*- and *trans*-isomer mixture. Repeated column chromatography on silica gel using PE/E (10%) afforded pure *trans*-isomer in the first fractions.


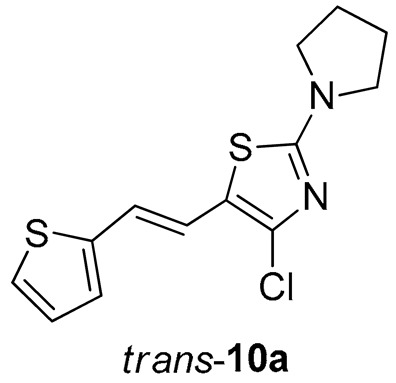


*(E)-4-chloro-2-(pyrrolidin-1-yl)-5-(2-(thiophen-2-yl)vinyl)thiazole* (*trans*-**10a**): 8 mg (isolated yield 32%), yellow oil, *R_f_*(PE/E = 20%) = 0.83; UV (ACN) *λ*_max_/nm (*ε*/dm^3^ mol^−1^ cm^−1^) 373 (33,945); ^1^H NMR (CDCl_3_, 600 MHz) *δ*/ppm: 7.15–7.14 (m, 1H), 6.96 (d, *J* = 4.5 Hz, 2H), 6.94 (d, *J* = 15.7 Hz, 1H), 6.57 (d, *J* = 15.7 Hz, 1H), 3.48 (t, *J* = 6.7 Hz, 4H), 2.07–2.05 (m, 4H; ^13^C NMR (CDCl_3_, 75 MHz) *δ*/ppm: 163.0, 142.8, 135.0, 127.6, 125.0, 123.8, 119.5, 117.9, 116.5, 49.3, and 25.7; MS (ESI) *m*/*z* (%, fragment): 297/299 (100); HRMS (*m*/*z*) for C_13_H_13_ClN_2_S_2_: [M + H]^+^calcd = 296.0209, and [M + H]^+^measured = 296.0206.

Thiazol-2-amines **6a** and **9a** were synthesized using **3a** (0.00046 mol, 1 eq), XPhos (0.000093 mmol, 0.2 eq), Pd(OAc)_2_ (0.00024 mmol, 0.05 eq), and Cs_2_CO_3_, (0.00069 mmol, 1.5 eq) dissolved in 2 mL of dioxane, and benzyl-amines (0.00096 mmol, 2 eq) were added. The reaction mixtures were purged with argon and heated to 120 °C in *sealed* tubes for 24 h. The reactions were always performed starting from pure isomers of **3a**, and the solvent was evaporated under pressure. The target isomer was purified by column chromatography on silica gel using E/EtOAc (0–60%) as eluent afforded in the first fractions traces of **3a** and in the last fractions mixture of isomers for **6a** (used later for photochemical reaction) and pure *trans*-isomer for **9a**.

*N-(piperidin-4-ylmethyl)-5-(2-(thiophen-2-yl)vinyl)thiazol-2-amine* (**6a**) (7%). Column chromatography on silica gel using E/EtOAc (60%) afforded 7 mg of a *trans-*isomer and XPhos ligand mixture. 

*N-(sec-butyl)-5-(2-(thiophen-2-yl)vinyl)thiazol-2-amine* (*trans*-**9a**) (10%). Column chromatography on silica gel using DCM/E (40%) afforded pure *trans*-isomer.


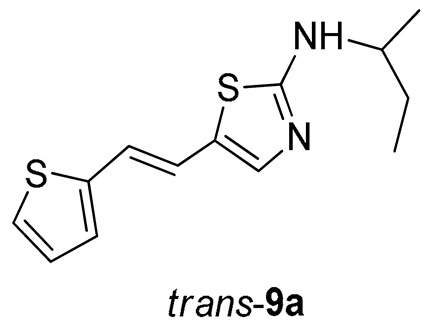


*(E)-N-(sec-butyl)-5-(2-(thiophen-2-yl)vinyl)thiazol-2-amine* (*trans*-**9a**): 10 mg (isolated yield 10%), yellow oil, *R_f_*(DCM/E = 40%) = 0.24; UV (ACN) *λ*_max_/nm (*ε*/dm^3^ mol^−1^ cm^−1^) 362 (10,237); ^1^H NMR (CDCl_3_, 600 MHz) *δ*/ppm: 7.12 (d, *J* = 4.9 Hz, 1H), 7.05 (s, 1H), 6.97–6.93 (m, 2H), 6.90 (d, *J* = 15.8 Hz, 1H), 6.59 (d, *J* = 15.8 Hz, 1H), 5.55 (s, 1H), 3.54–3.46 (m, 1H), 1.67–1.56 (m, 2H), 1.27 (d, *J* = 6.4 Hz, 1H), 0.98 (t, *J* = 7.6 Hz, 1H); ^13^C NMR (CDCl_3_, 75 MHz) *δ*/ppm: 168.3, 142.9, 139.0, 127.6, 125.4, 124.8, 123.5, 119.5, 119.2, 53.8, 29.7, 20.3, and 10.36; MS (ESI) *m*/*z* (%, fragment): 265 (100); HRMS (*m*/*z*) for C_13_H_16_N_2_S_2_: [M + H]^+^calcd = 264.0755, and [M + H]^+^measured = 264.0753.

Thiazolo-stilbene **8a** was synthesized by McMurry reaction. The reaction apparatus was under argon, Ar atmosphere while adding the reactants. The reaction is carried out in a three-necked flask (100 mL) with a magnetic stirrer. The zinc powder (0.00985 mol) and 30 mL of THF were added to the flask. The mixture was cooled to −5 °C, and titanium (IV) chloride, TiCl_4_ (0.0045 mol), was slowly added by a syringe with a temperature kept under 0 °C. The reaction mixture was warmed to room temperature, stirred for half an hour, then heated at reflux for 3 h. The mixture was cooled again to −5 °C, and the solution of two corresponding aldehydes (in 1:1.2 mol ratio, 0.00197 mol, 0.0012 mol) dissolved in THF (12 mL) was added dropwise. After aldehydes addition, the reaction mixture was heated at reflux for 2 h, then at room temperature overnight. The reaction was quenched with 10% aqueous sodium bicarbonate, NaHCO_3_ solution. The mixture was extracted with EtOAc (3 × 25 mL). The organic layers were dried under anhydrous MgSO_4_. Product **8a** was isolated by repeated column chromatography on silica gel using PE/E as a solvent system.

*2-(2-(2-methylthiazol-5-yl)vinyl)phenol* (**8a**) (10%). Column chromatography on silica gel using PE/E (90%) afforded 21 mg of a *cis-* and *trans-*isomer mixture. Repeated column chromatography on silica gel using PE/E (80%) afforded pure *trans*-isomer in the first fractions.


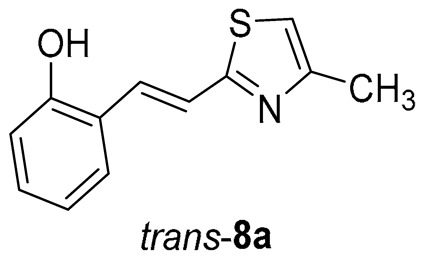


*(E)-2-(2-(2-methylthiazol-5-yl)vinyl)phenol* (*trans*-**8a**): 13 mg (isolated yield from the mixture 65%), yellow oil, *R_f_*(PE/E = 80%) = 0.50; UV (ACN) *λ*_max_/nm (*ε*/dm^3^ mol^−1^ cm^−1^) 345 (sh, 6109), 332 (21,971), 315 (sh, 20,036), 300 (sh, 19,398), 285 (23,628); ^1^H NMR (CDCl_3_, 600 MHz) *δ*/ppm: 7.69 (d, *J* = 15.9 Hz, 1H), 7.47 (d, *J* = 7.4 Hz, 1H), 7.16–7.09 (m, 2H), 7.01 (s, 1H), 6.90 (t, *J* = 7.1 Hz, 1H), 6.79 (d, *J* = 8.6 Hz, 1H), 6.25 (s, 1H), 2.76 (s, 3H); ^13^C NMR (CDCl_3_, 75 MHz) *δ*/ppm: 116.3, 154.2, 153.8, 128.8, 127.5, 126.0, 124.3, 122.5, 120.8, 116.1, 114.6, and 19.3; MS (ESI) *m*/*z* (%, fragment): 218 (20); 158 (100); HRMS (*m*/*z*) for C_12_H_11_NOS: [M + H]^+^calcd = 217.0561, and [M + H]^+^measured = 217.0557.

### 3.4. General Procedure for the Synthesis of the Electrocyclization Photoproducts ***1**–**10***

Mixtures of previously synthesized compounds **1a**–**10a** were dissolved in toluene p.a. (~2.5 × 10^−3^ M) and transferred to a quartz vessel (50 mL) with the addition of a catalytic amount of I_2_ and irradiated with 10 UV lamps at 313 nm in a photochemical reactor Rayonet for 1–5 h to achieve almost complete conversions. After removing the solvent by a rotary evaporator under reduced pressure, the photoproducts **1**–**5**, **7**, and **9** were purified by column chromatography from the remains of the starting substrates or unreacted aldehyde from the Wittig reaction. Pure photoproducts **1**–**5** were isolated and completely spectroscopically characterized by NMR and HRMS measurements. Compounds **7** and **9** were not isolated in a pure form, and further purification was impossible due to the insufficient amount of samples. Irradiation reaction did not produce photoproducts **6**, **8,** and **10**. 


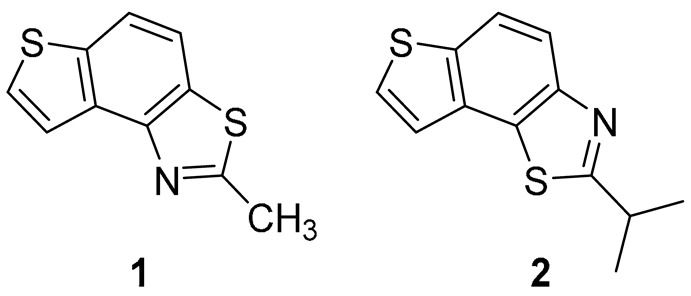


*2-methylthieno[2′,3′:5,6]benzo[1,2-d]thiazole* (**1**): 16 mg (isolated yield 30%), yellow oil, *R_f_*(PE/E = 5%) = 0.35; UV (ACN) *λ*_max_/nm (*ε*/dm^3^ mol^−1^ cm^−1^) 249 (40,956); ^1^H NMR (CDCl_3_, 600 MHz) *δ*/ppm: 7.91 (dd, *J* = 10.9, 8.6 Hz, 2H), 7.60 (d, *J* = 5.4 Hz, 1H), 7.44 (d, *J* = 5.2 Hz, 1H), 2.89 (s, 3H); ^13^C NMR (CDCl_3_, 75 MHz) *δ*/ppm: 164.9, 151.3, 136.5, 132.7, 129.8, 128.2, 122.3, 120.3, 118.9, and 20.0; MS (ESI) *m*/*z* (%, fragment): 206 (100); HRMS (*m*/*z*) for C_10_H_7_NS_2_: [M + H]^+^calcd = 205.0020, and [M + H]^+^measured = 205.0023.

*2-isopropylthieno[3′,2′:3,4]benzo[1,2-d]thiazole* (**2**): 14 mg (isolated yield 47%), yellow oil, *R_f_*(PE/E = 10%) = 0.40; UV (ACN) *λ*_max_/nm (*ε*/dm^3^ mol^−1^ cm^−1^) 251 (33,705); ^1^H NMR (CDCl_3_, 600 MHz) *δ*/ppm: 7.95 (d, *J* = 8.8Hz, 1H), 7.89 (d, *J* = 8.8 Hz, 1H), 7.59 (d, *J* = 5.4 Hz, 1H), 7.45 (d, *J* = 5.4 Hz, 1H), 3.52–3.45 (m, 1H), 1.53 (s, 3H), 1.51 (s, 3H); ^13^C NMR (CDCl_3_, 75 MHz) *δ*/ppm: 176.7, 151.0, 136.4, 132.9, 128.9, 128.1, 122.3, 120.2, 119.2, 34.0, and 23.1; MS (ESI) *m*/*z* (%, fragment): 234 (100); HRMS (*m*/*z*) for C_12_H_11_NS_2_: [M + H]^+^calcd = 233.0333, and [M + H]^+^measured = 233.0335.


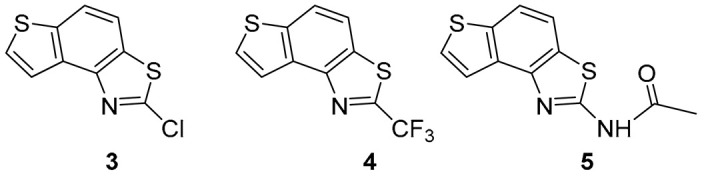


*2-chlorothieno[2′,3′:5,6]benzo[1,2-d]thiazole* (**3**): 5 mg (isolated yield 70%), yellow oil, *R_f_*(PE/E = 10%) = 0.45; UV (ACN) *λ*_max_/nm (*ε*/dm^3^ mol^−1^ cm^−1^) 214 (25,256); ^1^H NMR (CDCl_3_, 600 MHz) *δ*/ppm: 8.04 (d, *J* = 5.4 Hz, 1H), 7.99 (d, *J* = 8.8 Hz, 1H), 7.80 (d, *J* = 8.8 Hz, 1H), 7.72 (d, *J* = 6.1 Hz, 1H); ^13^C NMR (CDCl_3_, 75 MHz) *δ*/ppm: 152.7, 146.0, 138.9, 133.8, 132.1, 127.9, 121.7, 120.4, and 116.5; MS (ESI) *m*/*z* (%, fragment): 225/227 (75), 121 (100); HRMS (*m*/*z*) for C_9_H_4_ClNS_2_: [M + H]^+^calcd = 224.9474, and [M + H]^+^measured = 224.9475.

*2-(trifluoromethyl)thieno[2′,3′:5,6]benzo[1,2-d]thiazole* (**4**): 4 mg (isolated yield 20%), yellow oil, *R_f_*(PE/E = 10%) = 0.58; UV (ACN) *λ*_max_/nm (*ε*/dm^3^ mol^−1^ cm^−1^) 213 (26,252); ^1^H NMR (CDCl_3_, 600 MHz) *δ*/ppm: 8.08 (d, *J* = 5.5 Hz, 1H), 8.04 (d, *J* = 8.6 Hz, 1H), 7.89 (d, *J* = 8.6 Hz, 1H), 7.69 (d, *J* = 5.5 Hz, 1H); ^13^C NMR (CDCl_3_, 75 MHz) *δ*/ppm: 128.4, 122.3, 122.0, and 117.1 (the characteristic signal for the CF_3_ group is not clear); MS (ESI) *m*/*z* (%, fragment): 259 (100); HRMS (*m*/*z*) for C_10_H_4_F_3_NS_2_: [M + H]^+^calcd = 258.9737, and [M + H]^+^measured = 258.9740.

*N-(thieno[2′,3′:5,6]benzo[1,2-d]thiazol-2-yl)acetamide* (**5**): 2 mg (isolated yield 43%), yellow oil, *R_f_*(E/EtOAc = 60%) = 0.65; UV (ACN) *λ*_max_/nm (*ε*/dm^3^ mol^−1^ cm^−1^) 221 (17,611); ^1^H NMR (CDCl_3_, 600 MHz) *δ*/ppm: 9.36 (s, 1H), 7.82–7.81 (m, 2H), 7.76 (d, *J* = 8.6 Hz, 1H), 7.57 (d, *J* = 5.5 Hz, 1H), 2.37 (s, 3H); ^13^C NMR (CDCl_3_, 75 MHz) *δ*/ppm: 126.9, 121.2, 116.5, 117.4, and 29.6 (the signal for the amide carbon is not clear); MS (ESI) *m*/*z* (%, fragment): 249 (100), 207 (25), 121 (50); HRMS (*m*/*z*) for C_11_H_8_N_2_OS_2_: [M + H]^+^calcd = 248.0078, and [M + H]^+^measured = 248.0079.

### 3.5. Cholinesterase Inhibition Activity Measurements

AChE and BChE inhibition were determined using a modified spectrophotometric Ellman’s method [36]. Acetylthiocholine iodide (ATChI), S-butyrylthiocholine iodide (BTChI), AChE (EC 3.1.1.7, *Electrophorus electricus*, Type: V-S), BChE (EC 3.1.1.8, equine serum), and Trisma base were purchased from Sigma-Aldrich (St. Louis, MO, USA), while Ellman’s reagent 5,50-dithiobis-(2-nitrobenzoic acid) (DTNB) was purchased from Zwijndrecht (Antwerpen, Belgium). The reaction mixture contained 180 µL Tris-HCl buffer (50 mM, pH 8.0) or phosphate buffer, 10 µL of the enzyme (final concentration 0.03 U/mL), 10 µL of the tested solution, and 10 μL of DTNB (final concentration 0.3 mM). The reaction started with adding 10 μL of ATChI/BTChI (final concentration of 0.5 mM). The developing yellow color was measured at 405 nm over 6 min at room temperature using a 96-well microplate reader (IRE 96, SFRI Medical Diagnostics, Bordeaux, France). The experiment was run in triplicate. The percentage of enzyme inhibition was calculated from measured data according to the equation: Inhibition (%) = [(A_c_ − A_T_)/A._C_.]∙100, where A._C_. is the enzyme activity without the test sample, and A_T_ is the enzyme activity with the test sample, calculated as mean values ± standard deviation. In the control measurement, the tested compound was replaced by a buffer solution. Non-enzymatic hydrolysis was measured as blank for each measurement. The IC_50_ value was calculated by a nonlinear fit of compound concentration (log) values vs. response.

### 3.6. Computational Study

Geometry optimizations and UV-Vis spectra are calculated using the Gaussian16 program package [37]. The geometries are optimized at the M06-2X/6-31G(d) level of theory, and the minima on potential energy surface for all structures are verified by performing vibrational analysis. UV-Vis spectra are calculated using the (CPCM)TD-PBEh1PBE/6-31+G(d)//M06-2X/6-31G(d) model. The conductor-like polarizable continuum model of solvation (CPCM) [38] was used to describe the solvent effect of acetonitrile. A molecular docking study was performed using the Autodock program package [39], with crystal structure 7AIY.pdb [40] for BChE taken from the Protein Data Bank. The protein was prepared prior to molecular docking analysis by co-crystallized ligand using MGL Tools 1.5.6, nonpolar hydrogen bonds were fused, Kollman-type charges were assigned, and the proteins were saved to the file. pdbqt form. The ligands were prepared using structures optimized at M062X/6-31G(d) computational model, polar hydrogens were preserved, the number of rotatable bonds was identified, and ligands were converted to pdbqt format using MGL Tools-1.5.6. The docking results were obtained using the Lamarckian Genetic Algorithm, with 25 requested genetic algorithm dockings with 25 binding poses for each ligand. The residues of the enzymes were kept rigid during the docking. The model system for QM cluster-continuum calculations is prepared by modifying residues of the active site: carboxyl and amino groups are removed from α carbons and substituted with hydrogen atoms, thus deriving methyl groups. The geometry optimization of complexes is then carried out at (CPCM)B3LYP/6-31g(d) level of theory using the Gaussian program package; coordinates of α carbons were held frozen, so the architecture of the active site initially present in the enzyme is conserved during the optimization. The protein environment is described by implicit solvation, with dielectric constant *ε* = 4.

## 4. Conclusions

New thieno-thiazolostilbenes and thienobenzo-thiazoles were designed and synthesized, and their potential inhibitory activity toward cholinesterases was tested and compared with their analogs, naphtho-oxazoles. Thieno-thiazolostilbenes **1a**–**10a** were obtained as mixtures of *cis*- and *trans-*isomers, some by Wittig reaction, while others by continued Buchwald-Hartwig amination or by McMurry reaction. From thieno-thiazolostilbenes **1a**–**10a**, corresponding thienobenzo-thiazoles **1**–**5**, **7**, and **9** were successfully obtained by photochemical cyclization. All isolated pure compounds were evaluated for cholinesterases’ inhibitory activity. Key determinants in a structure-activity relationship were the type of heterocyclic ring, the molecule’s geometry, and, where possible, the type and position of a substituent. Among stilbene derivatives, the best result is achieved by the derivative with a phenolic ring *trans*-**8a** and its thiophene analog *trans*-**1a**. The phenolic thiazolostilbene is the most active of all tested compounds. Three of the five tested photocyclization thiazole products achieved an IC_50_ value, compound **1** with methyl substituent, **3** with chlorine, and molecule **4** with trifluoromethyl. The achieved inhibitory activity is very promising, with IC_50_ values in the range of common reversible inhibitor huperzine A. Comparing electrocyclic thiazoles and oxazoles leads to the observation that these two rings are similar: one of the similarities being the inactivity towards the AChE enzyme. As the tested structures showed drastically better inhibition of the BchE than AchE, these results are very important as BchE has been attracting growing attention due to its positive role in Alzheimer’s disease. A computational study revealed that thieno-benzo-thiazoles and naphtho-oxazole that showed the best inhibitory activity toward BchE owe that to the rigidity of the main scaffold that can block the esteratic subsite of the active site, accompanied by π-π stacking between the ligand and the residues of the active site, that additionally stabilizes the formed complex. For more flexible thieno-thiazolostilbene *trans*-**8**, the main stabilizing interaction responsible for the inhibitory activity is its ability to form H-bonds with residues of the active site (due to the presence of the hydroxyl group at the phenyl ring), accompanied by some hydrophobic interactions.

## Figures and Tables

**Table 1:** Experimental and calculated values of λ_max_ of thieno-thiazolostilbenes (*cis*-**1a**, *trans*: **1a**, **2a**, **3a**, and **10a**) and thienobenzo-thiazoles **1**–**5** along with main assignments obtained by calculations. The computational model (CPCM)TD-PBEh1PBE/6-31+G(d) (in acetonitrile) is employed, with geometries optimized at M06-2X/6-31G(d) level of theory. H and L denote the highest occupied and lowest unoccupied molecular orbital, respectively.

Molecule	λ_max_/nm (exp)	λ_max_/nm (calc)	Assignment
*trans*-**2a**	318	328	H → L
*trans*-**3a**	342	357	H → L
*trans*-**10a**	373	384	H → L
*cis*-**1a**	315	323	H → L
*trans*-**1a**	320	349	H → L
**1**	249	245	H − 1→ L H → L + 1
**2**	251	242	H − 1→ L H − 1→ L + 1
**3**	214	203	H − 1→ L + 4
**4**	213	204	H − 1→ L + 3
**5**	221	211	H − 1→ L + 2

**Table 2:** Calculated IC_50_ values and inhibitions percents for thiazoles **1a**–**10a** and **1**–**5,** and oxazoles **11**–**23**.

Comp. Number	Structure	IC_50_ (μM) BChE	Inhibition (%) ^(a)^	Comp. Number	Structure	IC_50_ (μM) BChE	Inhibition (%) ^(a)^
**1**	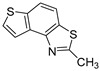	107.3	79.1 (500)	**11**	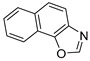	-	0.9 (188.0)
**2**	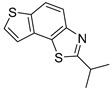	-	46.8 (200)	**12**	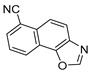	-	40.9 (176.0)
**3**	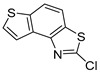	51.1	75.4 (250)	**13**	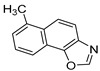	350.7	57.8 (396.8)
**4**	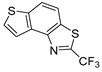	41.9	70.8 (200)	**14**	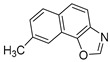	154.7	63.3 (173.6)
**5**	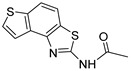	-	24.9 (250)	**15**	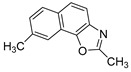	44.4	98.3 (368.6)
*cis*-**1a**	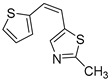	-	36.4 (500)	**16**	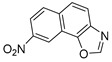	137.4	64.9 (201.7)
*trans*-**1a**	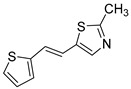	181.4	64.0 (500)	**17**	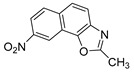	-	16.8 (159.5)
*cis*-**2a**	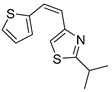	-	45.5 (250)	**18**	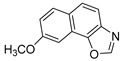	-	38.0 (273.6)
*trans*-**2a**	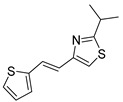	-	45.8 (250)	**19**	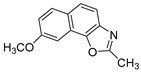	170.2	81.9 (405.2)
*trans*-**3a**	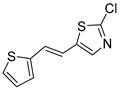	-	10.0 (250)	**20**	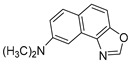	183.9	82.2 (364.2)
**5a**	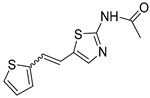	-	30.6 (250)	**21**	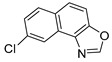	-	32.6 (312.3)
*trans*-**8a**	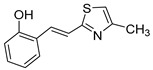	25.2	80.5 (250)	**22**	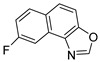	127.7	69.9 (315.8)
*trans*-**9a**	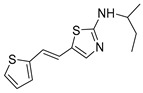	- ^(b)^	47.7 (100)	**23**	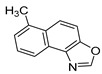	-	20.6 (297.5)
*trans*-**10a**	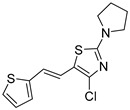	- ^(b)^	25.3 (100)	**Huperzine A**		53.4	

^(a)^ Concentrations in μM are given in brackets for the maximal effect measured. ^(b)^ For compounds *trans*-**9a** and *trans*-**10a,** it was impossible to determine the IC_50_ values due to the coloration of the samples.

## Data Availability

The data presented in this study are available on request from the corresponding author. The data are not publicly available due to privacy.

## Supplementary Materials

The following supporting information can be downloaded at: https://www.mdpi.com/article/10.3390/molecules28093781/s1: ^1^H and ^13^C NMR spectra of compounds **1a**–**10a** and **1**–**5**, **7,** and **9** (Figures S1–S64); MS and HRMS analyses of compounds **1a**–**10a** and **1**–**5**, **7,** and **9** (Figures S65–S81); Cartesian coordinates of studied thieno-thiazolostilbenes and thienobenzo-thiazoles, and Cartesian coordinates of molecules docked into the active site of BChE. Table S1: Results obtained by molecular docking.

## Sample Availability

Not available.
