# Supplementary material for: Thieno-Thiazolostilbenes, Thienobenzo-Thiazoles, and Naphtho-Oxazoles: Computational Study and Cholinesterase Inhibitory Activity
Source: Molecules. 2023 Apr 27;28(9):3781. doi: 10.3390/molecules28093781 (PMC10180155; doi:10.3390/molecules28093781)
Supplement: Supplementary file 1 [file molecules-28-03781-s001.zip › molecules-2348300-SI.pdf]

Supplementary Materials

Article

Thieno-Thiazolostilbenes, Thienobenzo-Thiazoles, and Naphtho-Oxazoles: Computational Study and Cholinesterase Inhibitory Activity

Milena Mlakić [1], Ema Đurčević [1], Ilijana Odak [2], Danijela Barić [3,*], Ines Juričević [2], Ivana Šagud [1,4], Franko Burčul [5], Zlata Lasić [6], Željko Marinić [7] and Irena Škorić [1,*]

[1] Department of Organic Chemistry, Faculty of Chemical Engineering and Technology, University of Zagreb, Marulićev trg 19, HR-10000 Zagreb, Croatia
[2] Department of Chemistry, Faculty of Science and Education, University of Mostar, Matice hrvatske bb, 88000 Mostar, Bosnia and Herzegovina
[3] Group for Computational Life Sciences, Division of Physical Chemistry, Ruđer Bošković Institute, Bijenička Cesta 54, HR-10000 Zagreb, Croatia
[4] Croatian Agency for Medicinal Products and Medical Devices, Ksaverska Cesta 4, HR-10000 Zagreb, Croatia
[5] Department of Analytical Chemistry, Faculty of Chemistry and Technology, University of Split, Ruđera Boškovića 35, HR-21000 Split, Croatia
[6] Teva api Analytical R&D, Pliva, Prilaz Baruna Filipovića 25, HR-10000 Zagreb, Croatia; zlata.lasic01@pliva.com
[7] NMR Center, Rudjer Bošković Institute, Bijenička Cesta 54, HR-10000 Zagreb, Croatia;
* Correspondence: dbaric@irb.hr (D.B.); iskoric@fkit.hr (I.Š.); Tel.: +385-1-4571-385 (D.B.); +385-1-4597-241 (I.Š.)

1. ^{1}H and ^{13}C NMR spectra of compounds **1a–10a** and **1–5, 7** and **9** (Figures S1-S64).

2. MS and HRMS analyses of compounds **1a–10a** and **1–5, 7** and **9** (Figures S65-S81).

3. Geometries of studied thieno-thiazolostilbenes and thienobenzo-thiazoles

4. Table S1.

4. Geometries of optimized complexes of molecules docked into the active site of BChE

1. ^{1}H and ^{13}C NMR spectra of compounds 1a–10a and 1–7

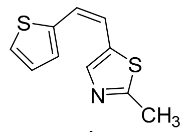

cis-**1a**

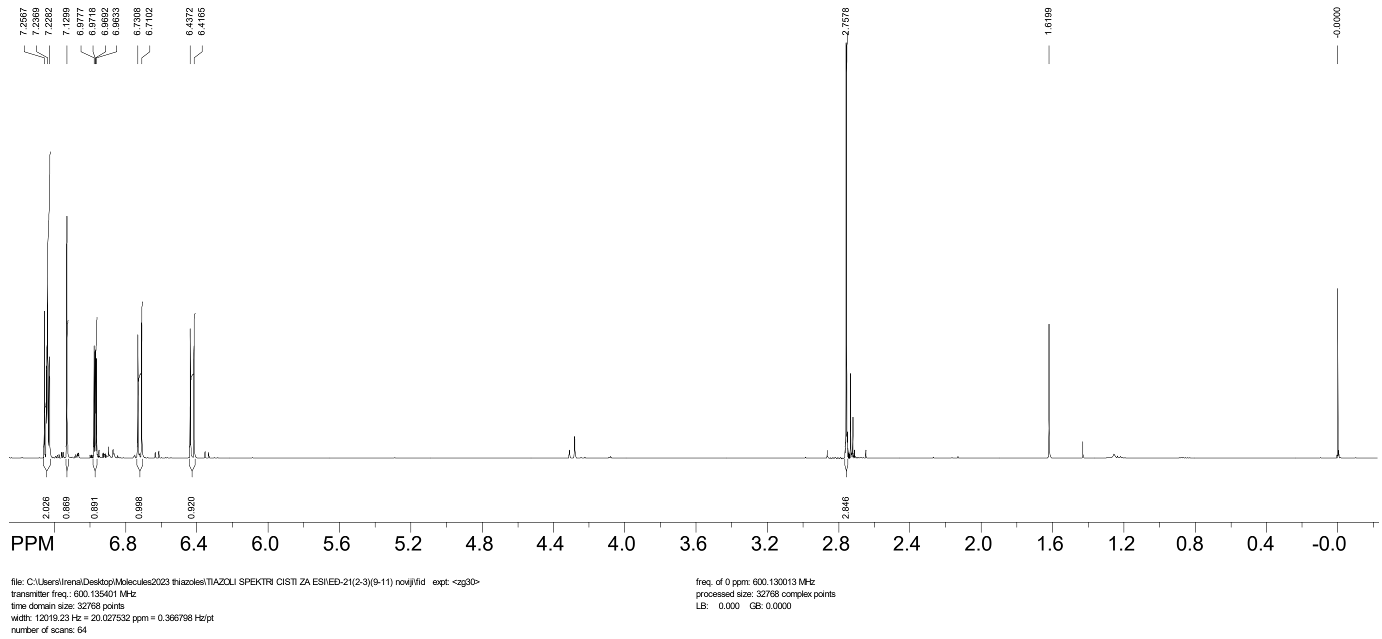

Figure S1. ^{1}H NMR spectrum ($CDCl_3$) of *cis*-**1a**.

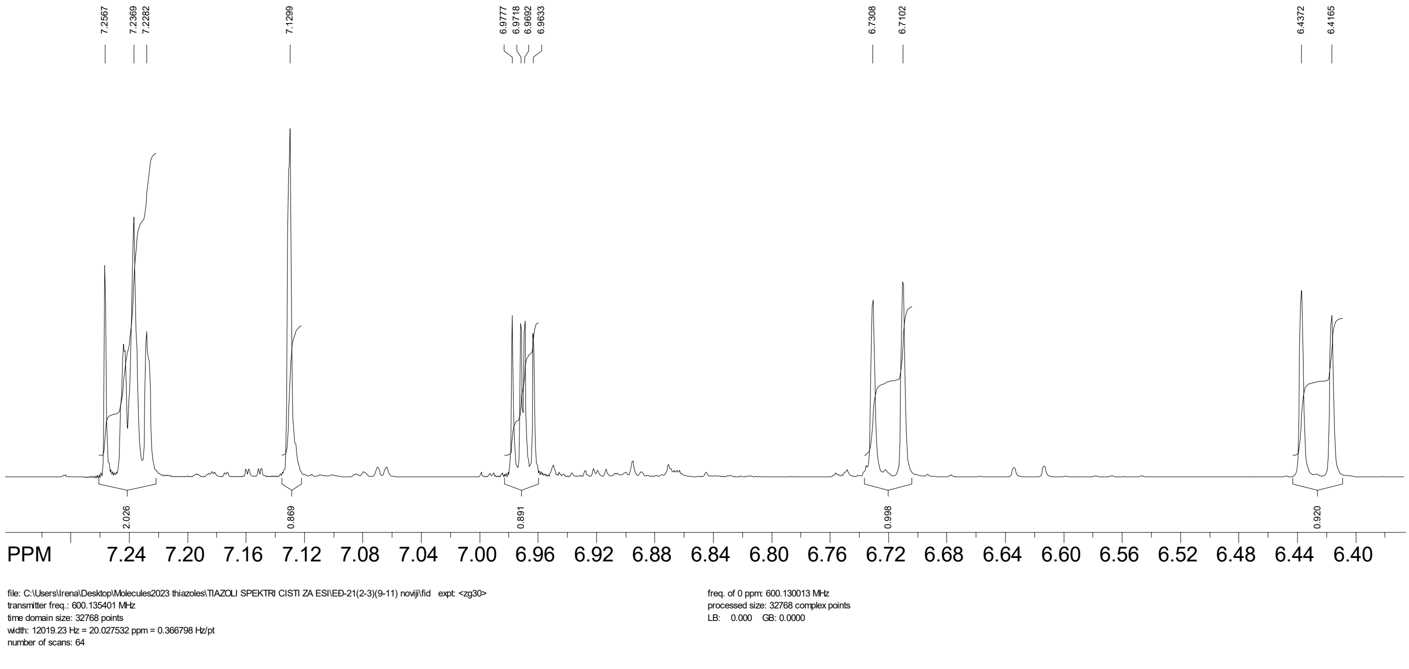

Figure S2. Aromatic part of the ^{1}H NMR spectrum ($CDCl_3$) of *cis*-**1a**.

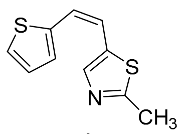

cis-**1a**

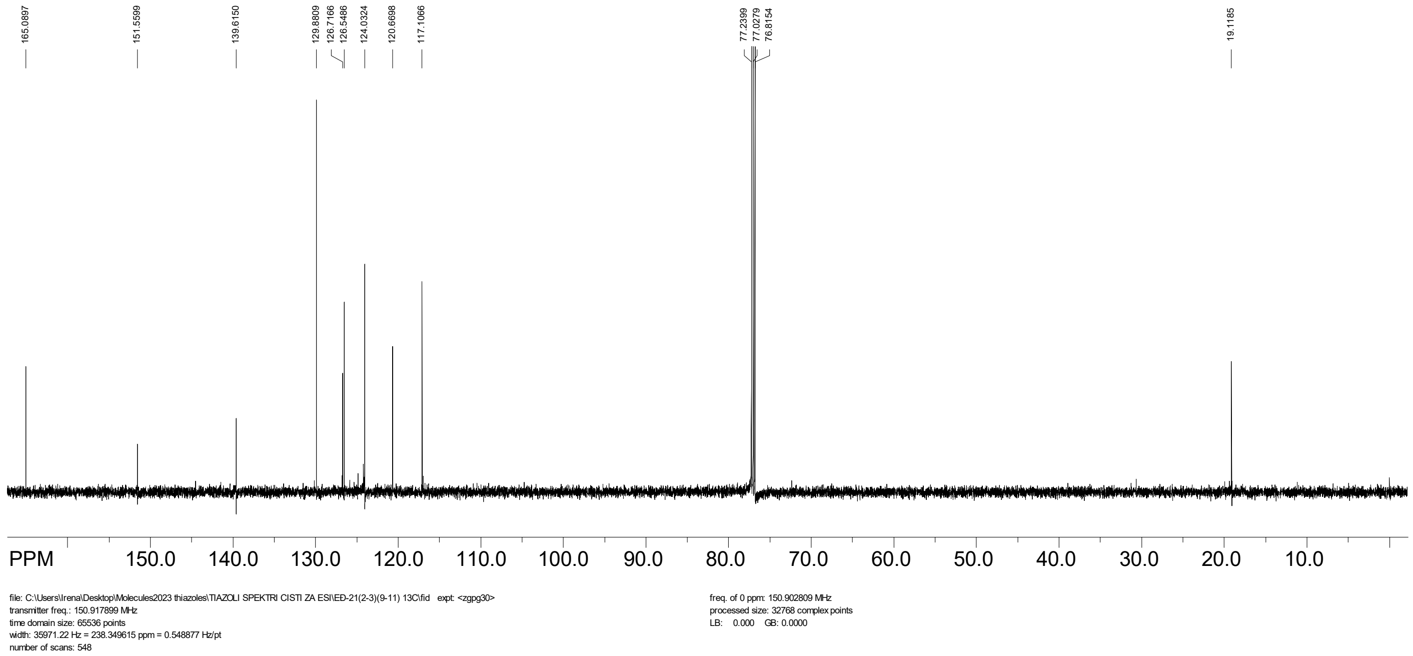

Figure S3. ^{13}C NMR spectrum ($CDCl_3$) of *cis*-**1a**.

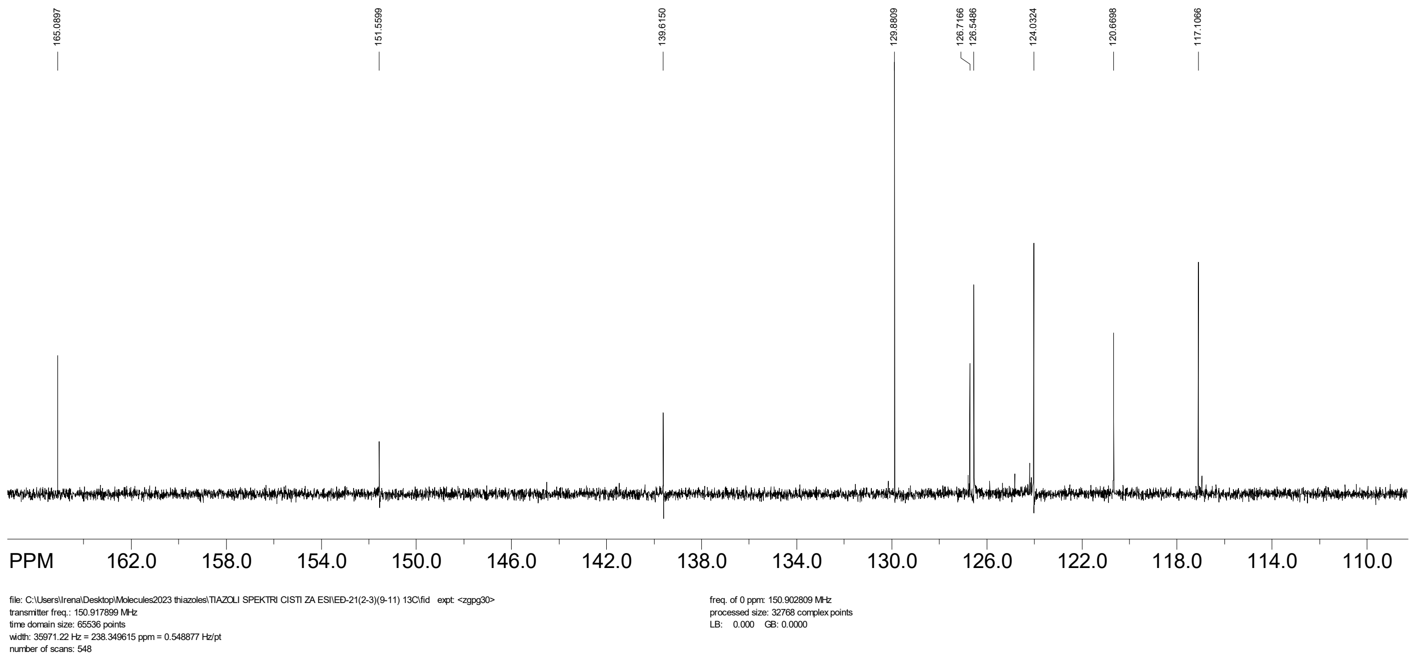

Figure S4. Aromatic part of the ^{13}C NMR spectrum ($CDCl_3$) of *cis*-**1a**.

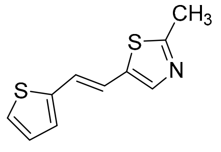

trans-**1a**

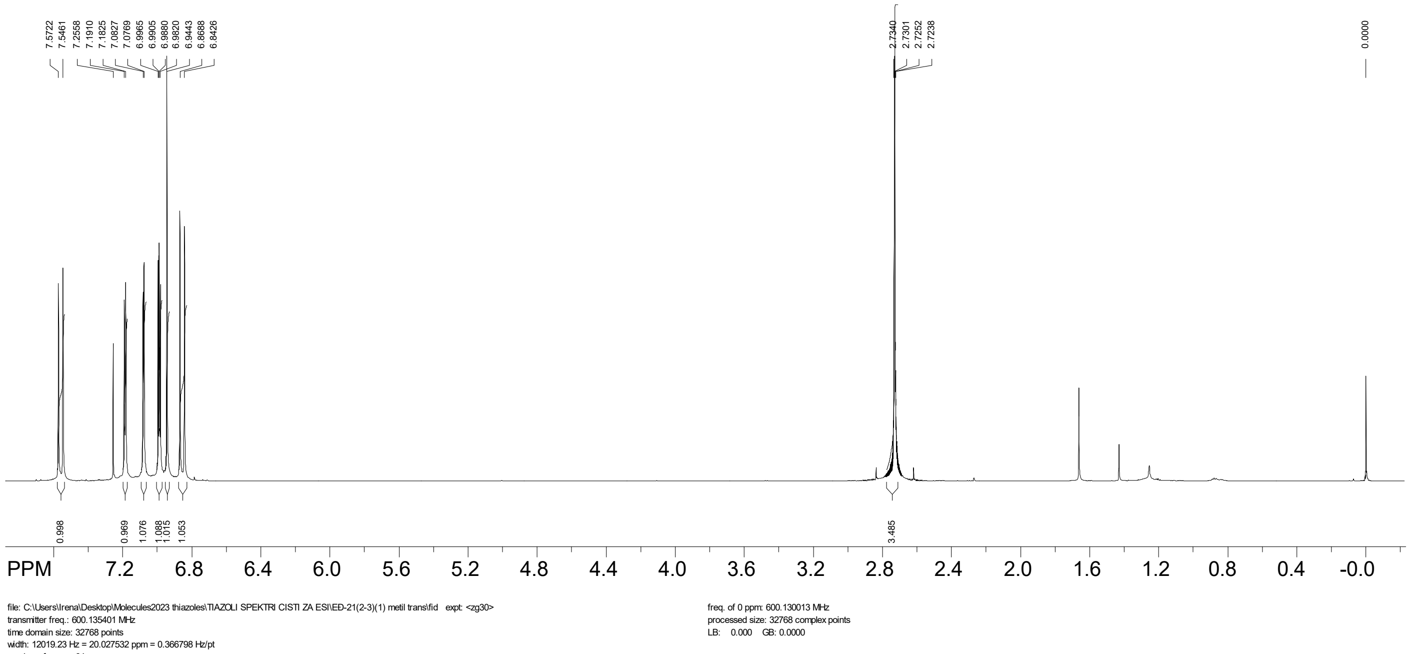

Figure S5. ^{1}H NMR spectrum ($CDCl_3$) of *trans*-**1a**.

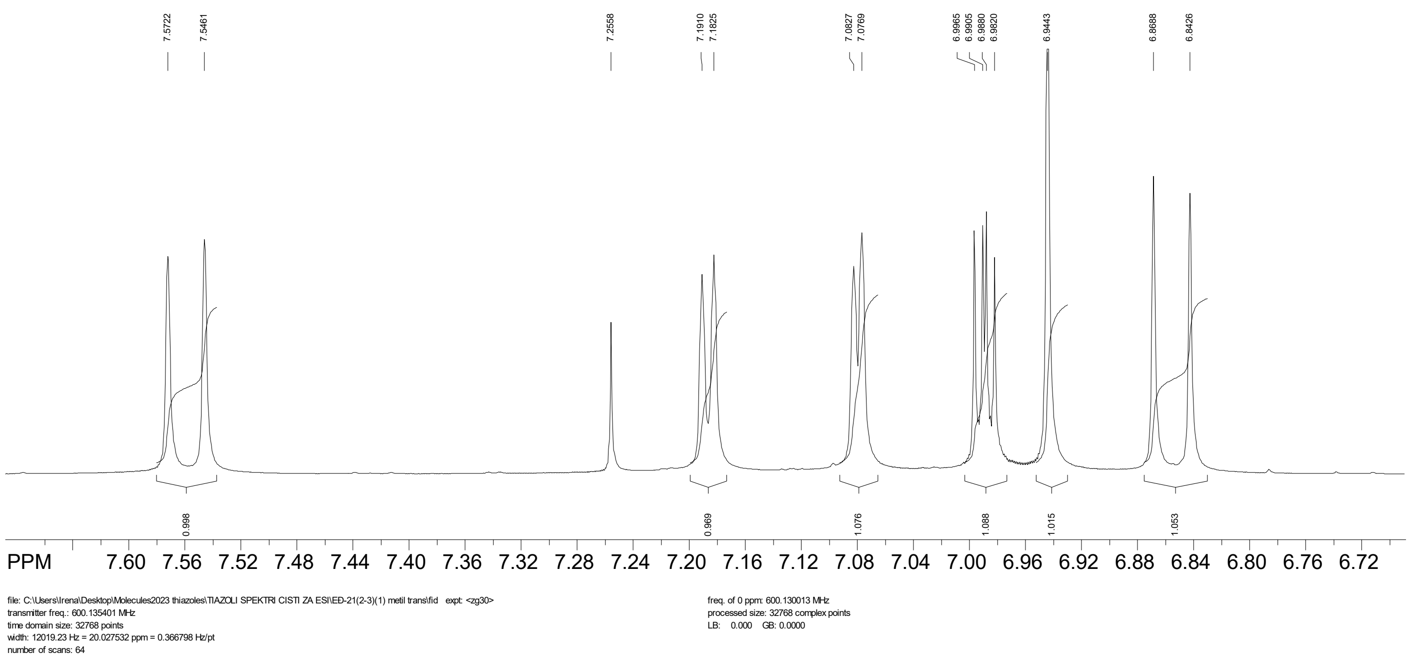

Figure S6. Aromatic part of the ^{1}H NMR spectrum ($CDCl_3$) of *trans*-**1a**.

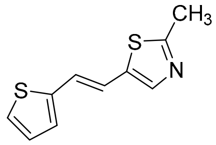

trans-**1a**

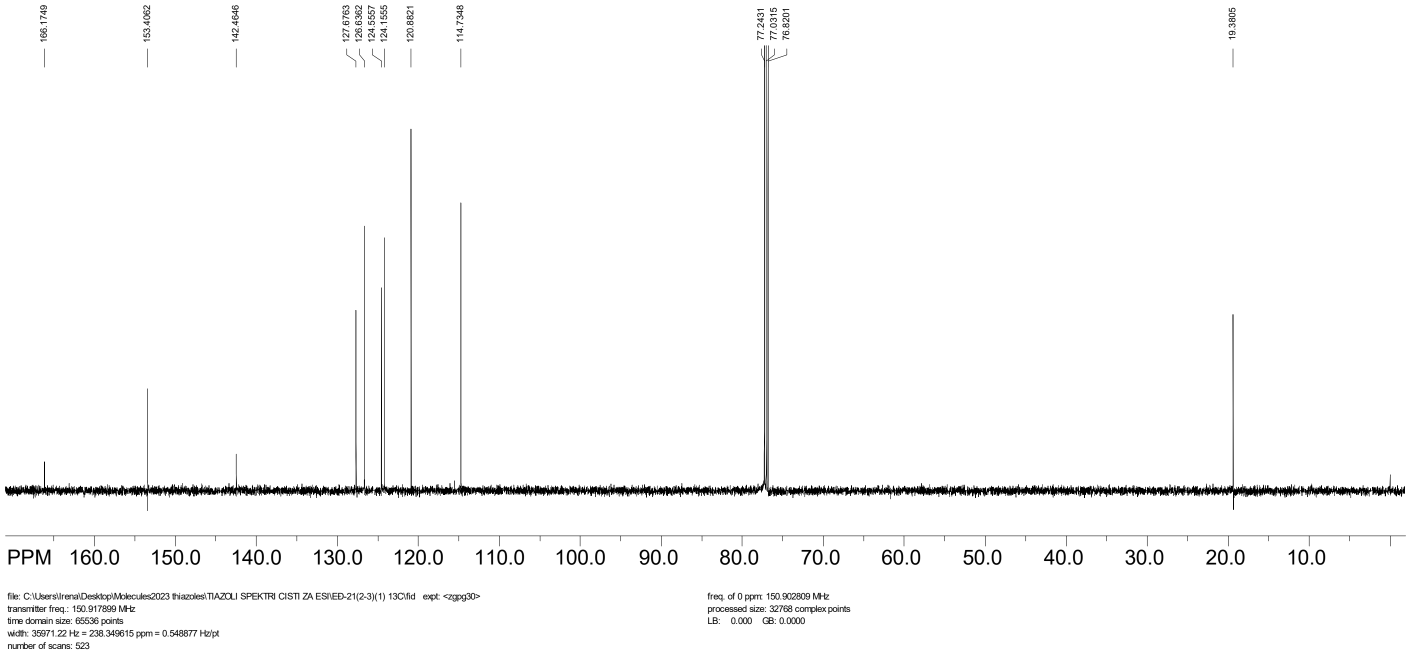

Figure S7. ^{13}C NMR spectrum ($CDCl_3$) of *trans*-**1a**.

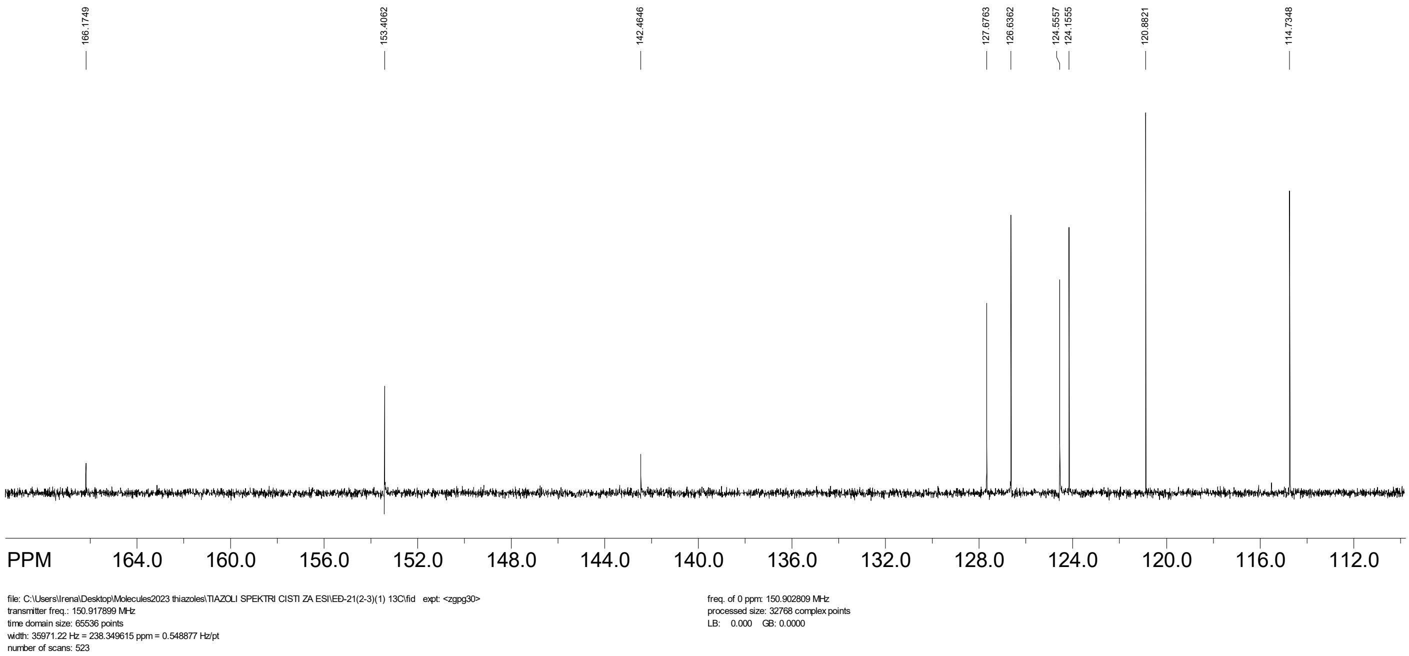

Figure S8. Aromatic part of the ^{13}C NMR spectrum ($CDCl_3$) of *trans*-**1a**.

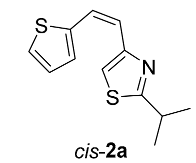

cis-**2a**

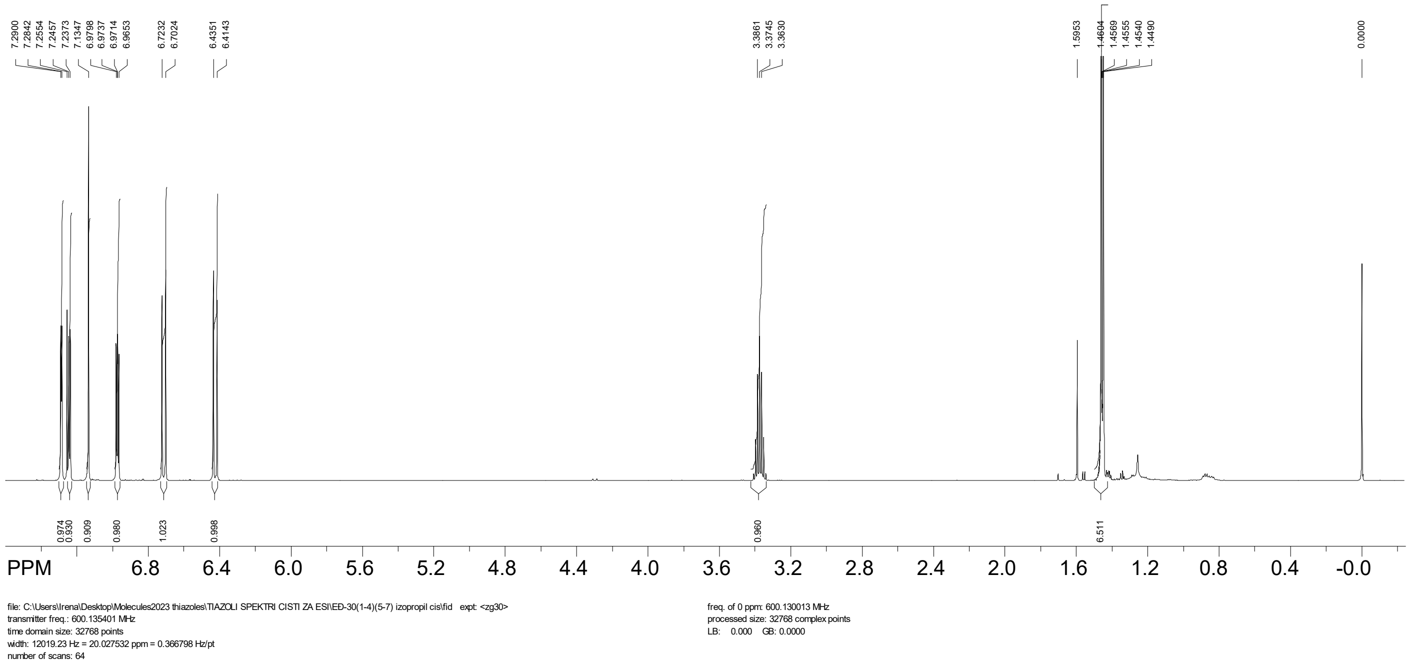

Figure S9. ^{1}H NMR spectrum ($CDCl_3$) of *cis*-**2a**.

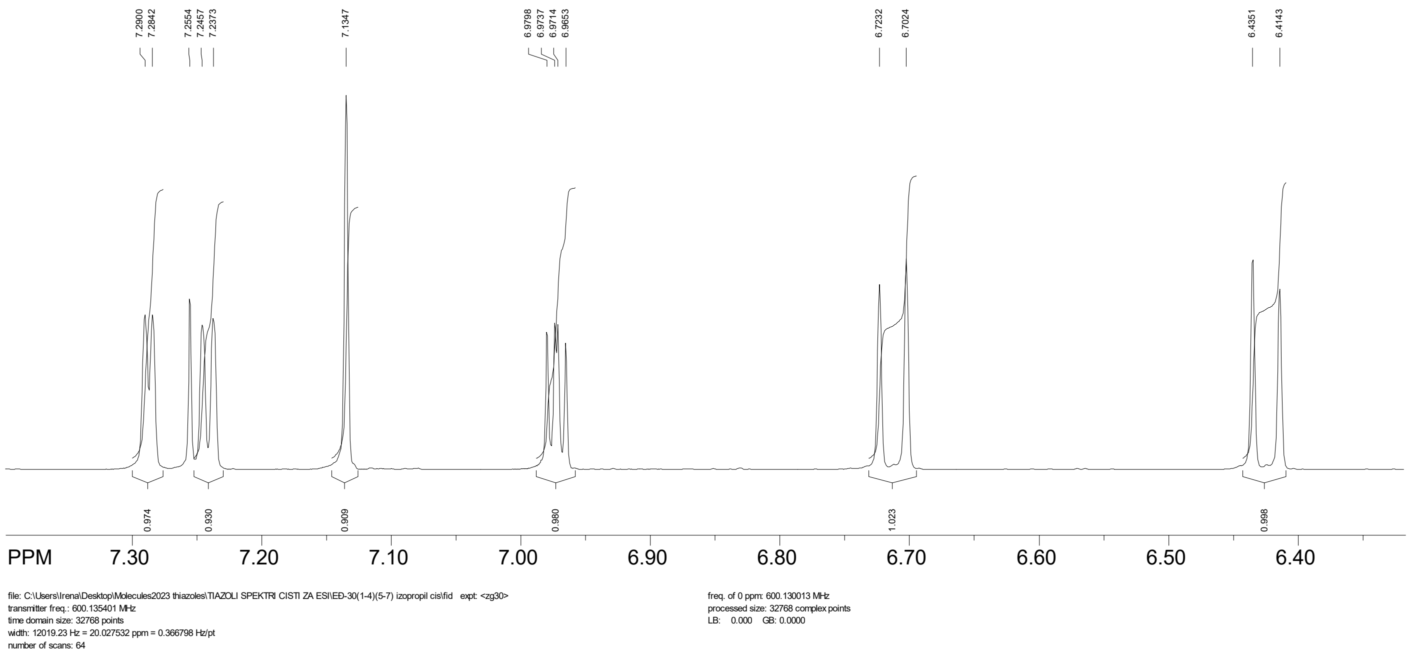

Figure S10. Aromatic part of the ^{1}H NMR spectrum ($CDCl_3$) of *cis*-**2a**.

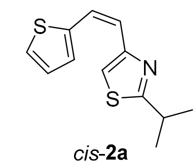

cis-**2a**

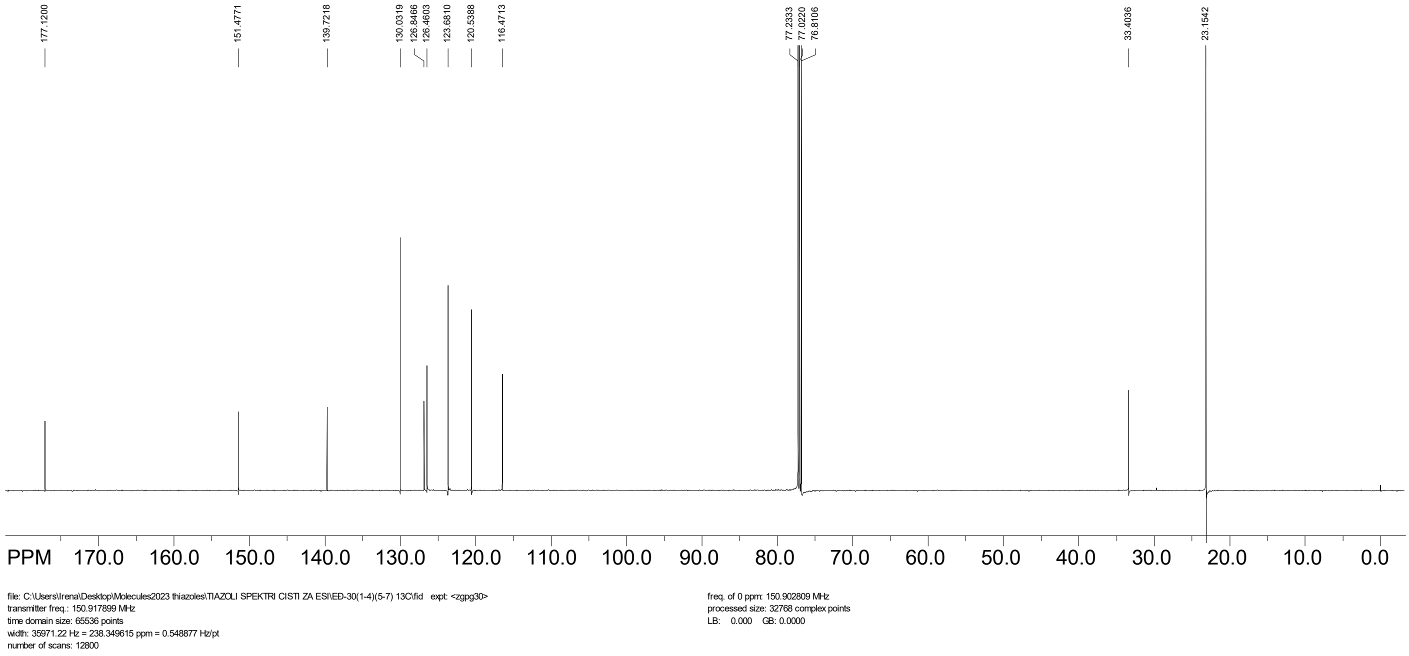

Figure S11. ^{13}C NMR spectrum ($CDCl_3$) of *cis*-**2a**.

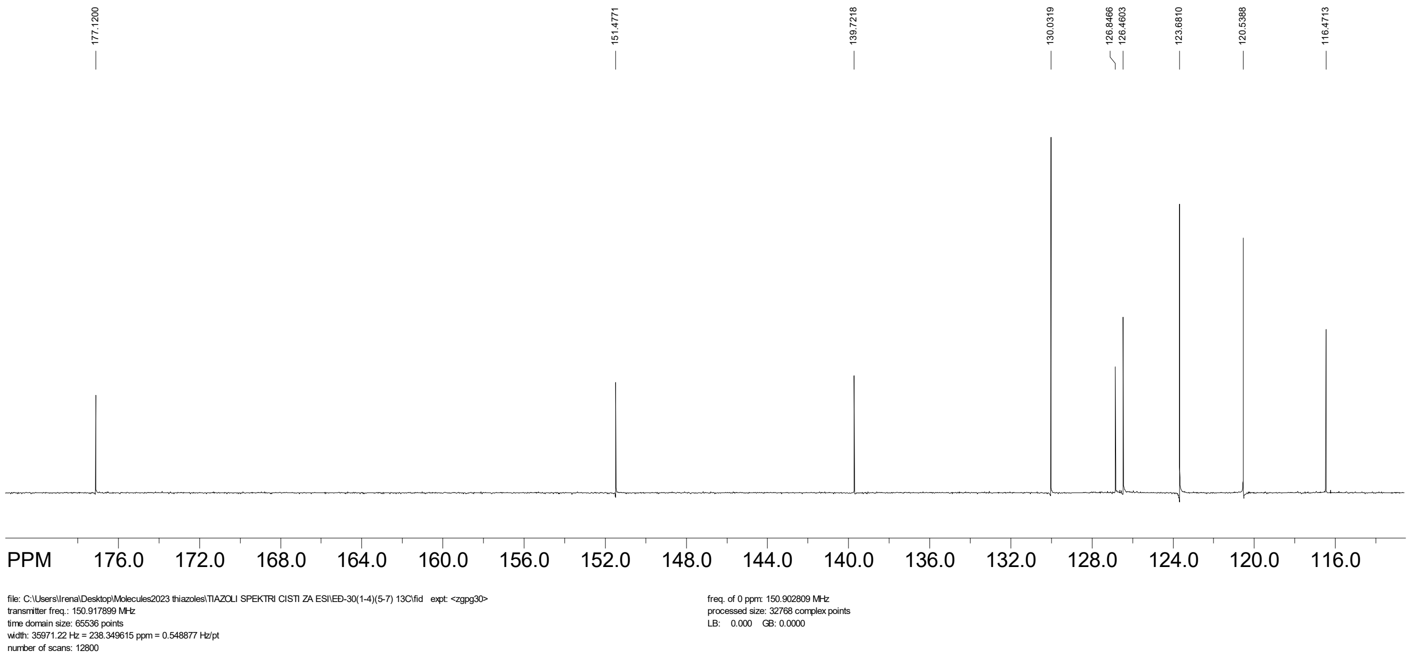

Figure S12. Aromatic part of the ^{13}C NMR spectrum ($CDCl_3$) of *cis*-**2a**.

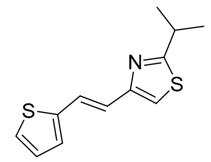

trans-**2a**

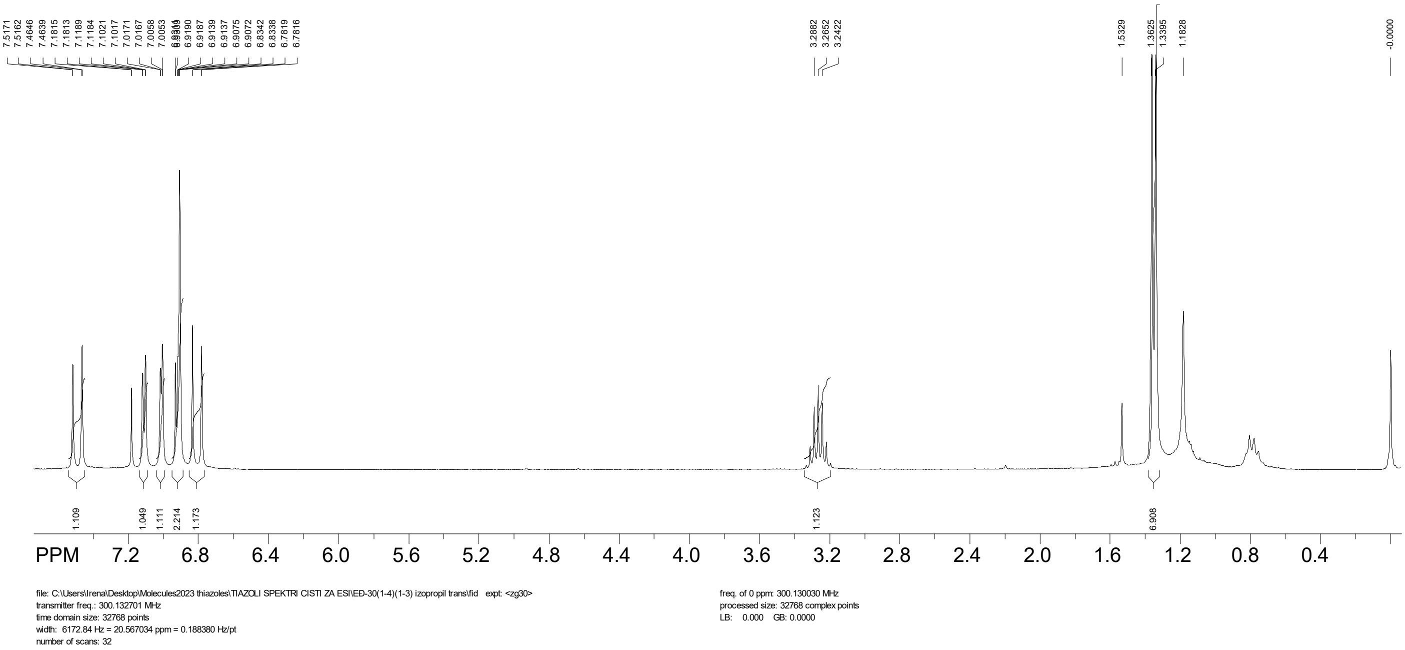

Figure S13. ^{1}H NMR spectrum ($CDCl_3$) of *trans*-**2a**.

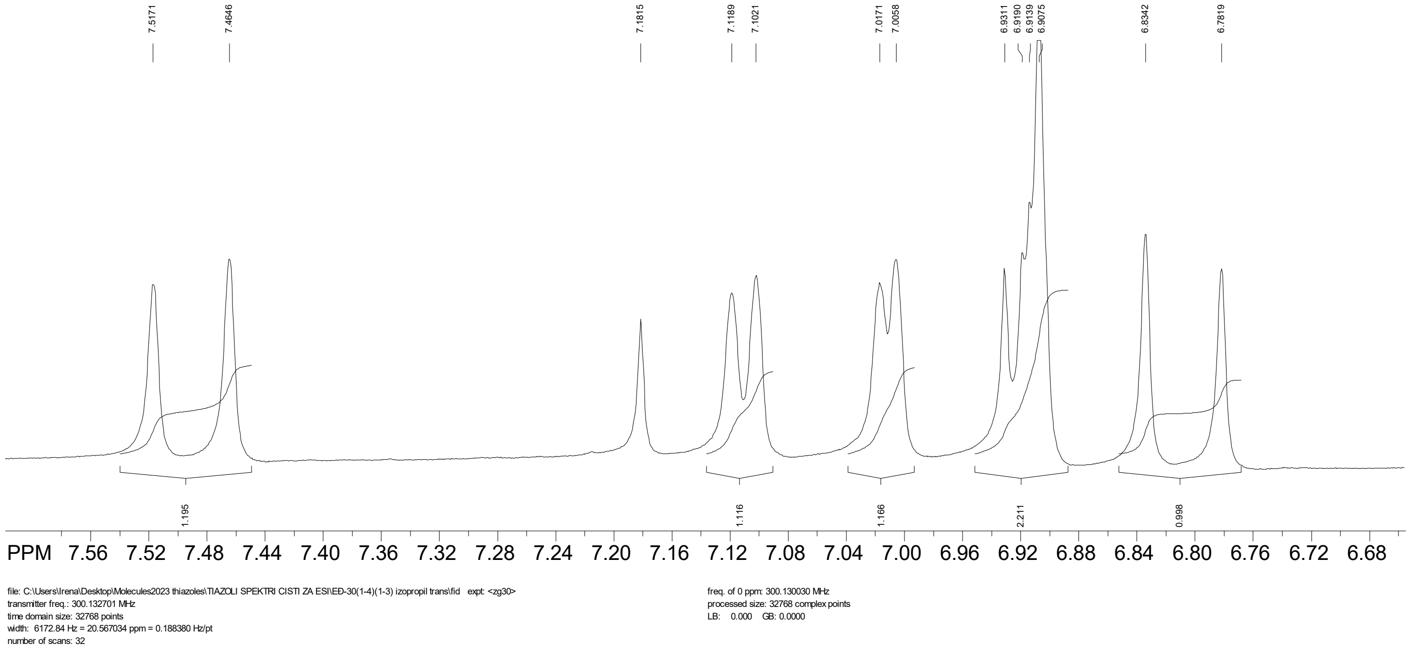

Figure S14. Aromatic part of the ^{1}H NMR spectrum ($CDCl_3$) of *trans*-**2a**.

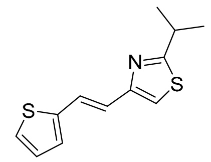

trans-**2a**

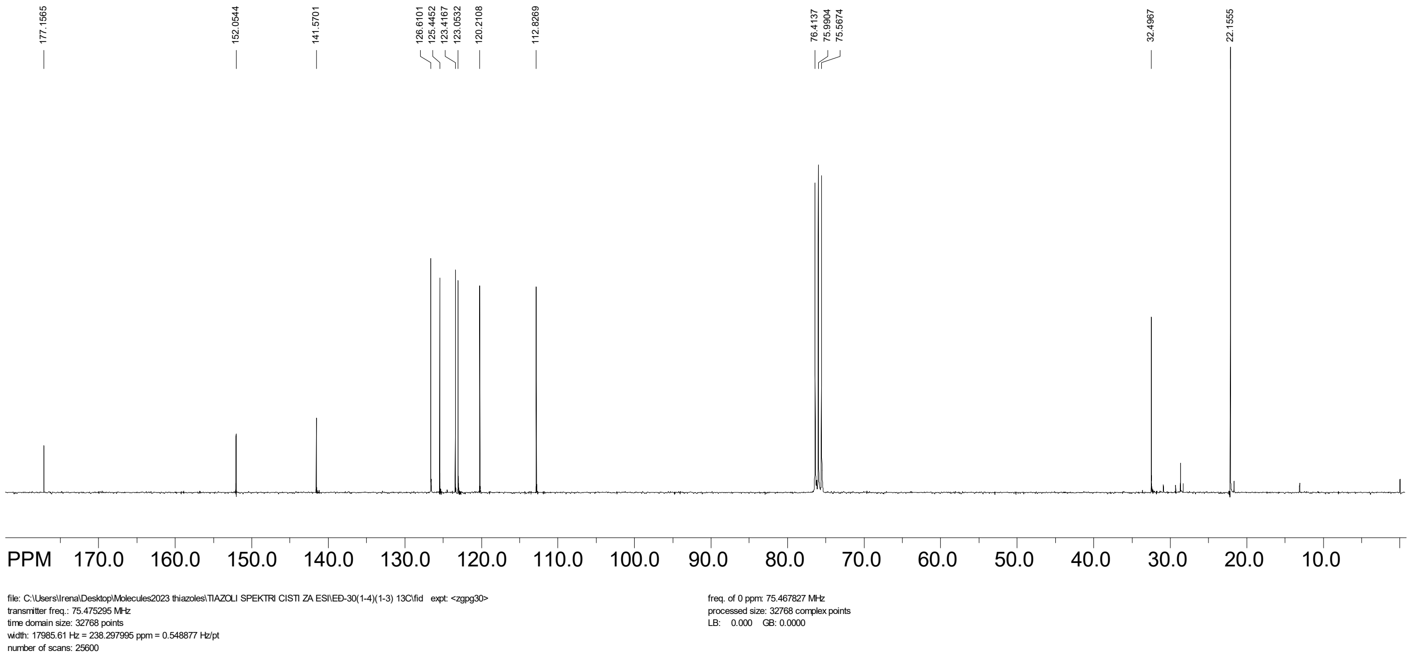

Figure S15. ^{13}C NMR spectrum ($CDCl_3$) of *trans*-**2a**.

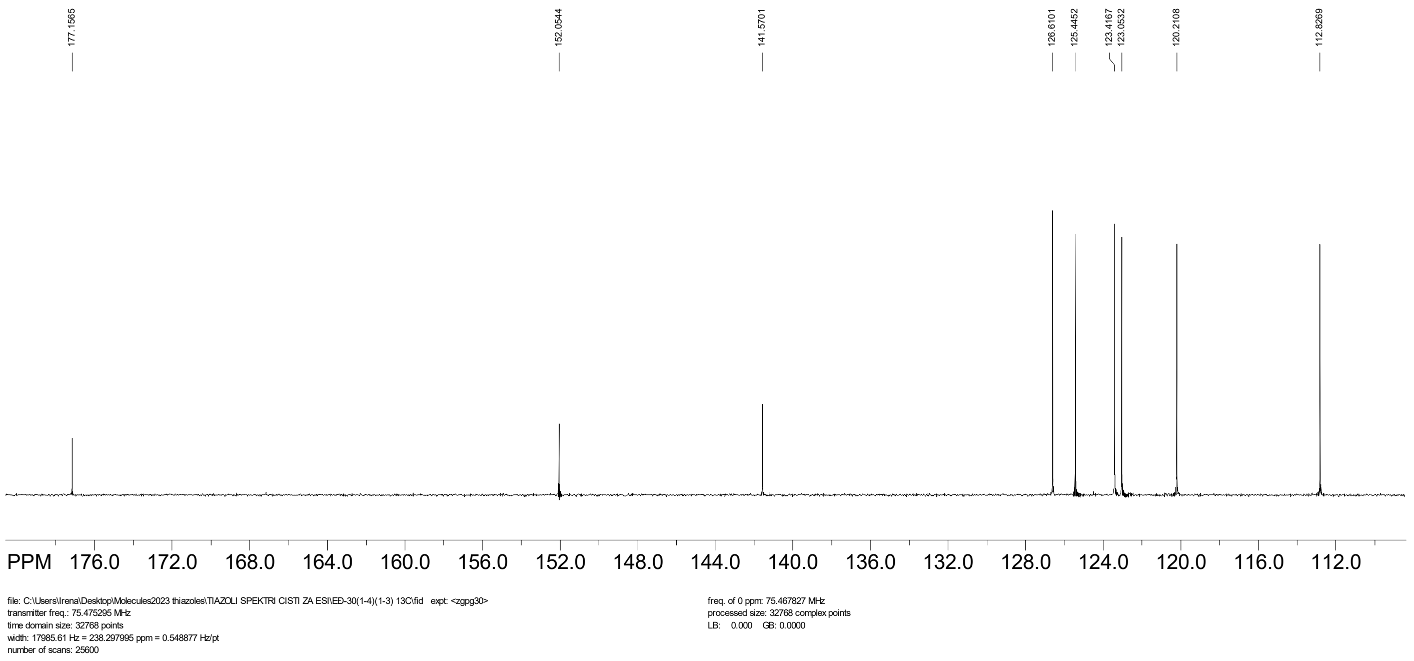

Figure S16. Aromatic part of the ^{13}C NMR spectrum ($CDCl_3$) of *trans*-**2a**.

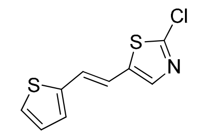

trans-**3a**

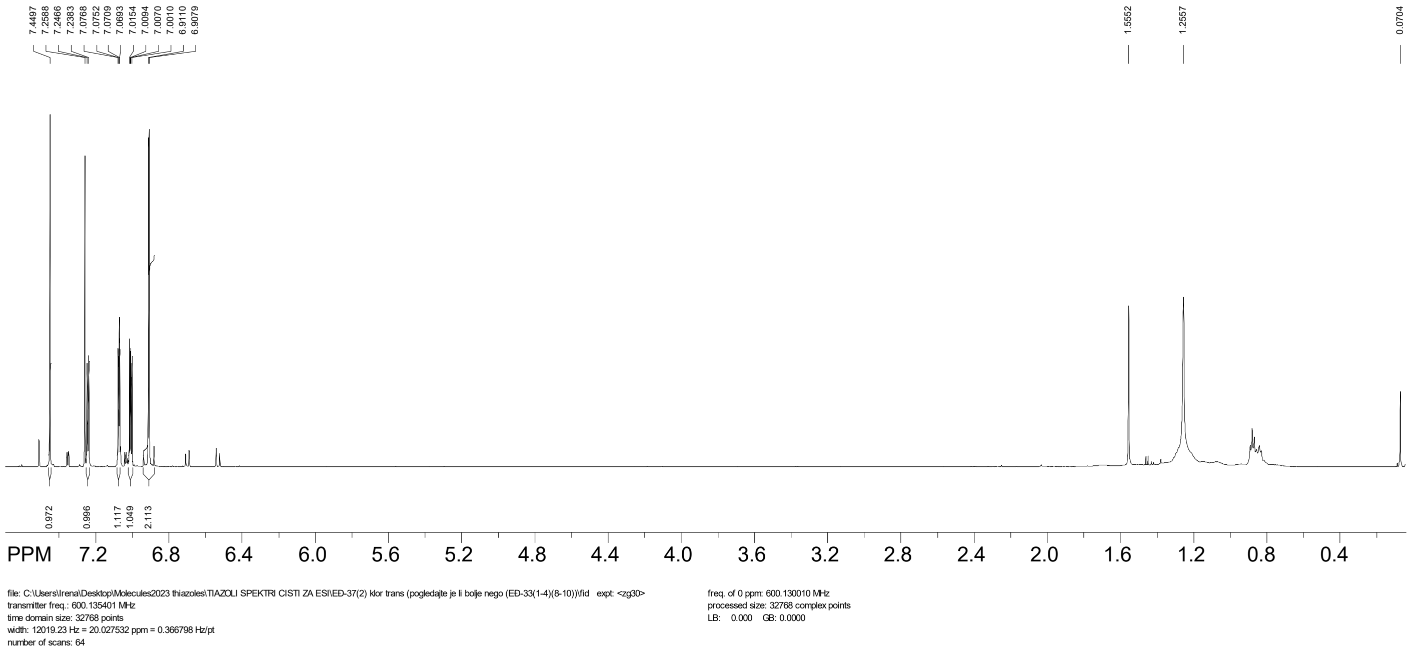

Figure S17. ^{1}H NMR spectrum ($CDCl_3$) of *trans*-**3a**.

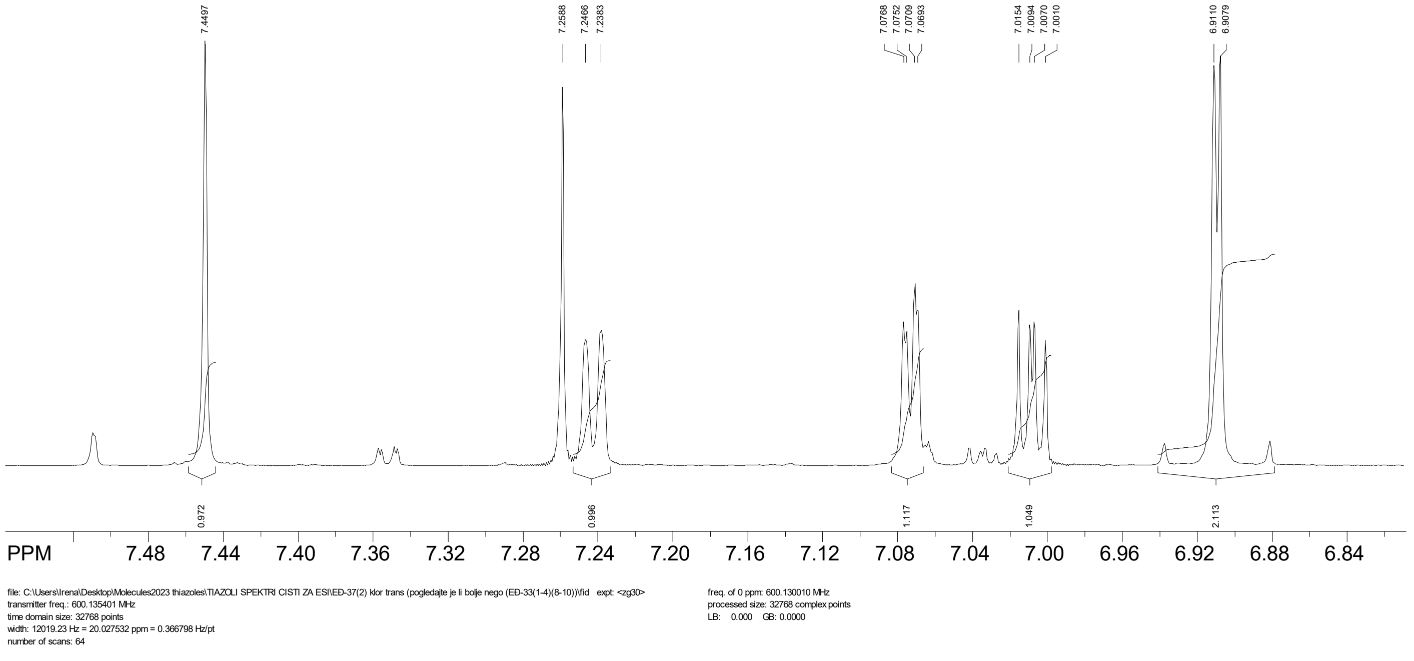

Figure S18. Aromatic part of the ^{1}H NMR spectrum ($CDCl_3$) of *trans*-**3a**.

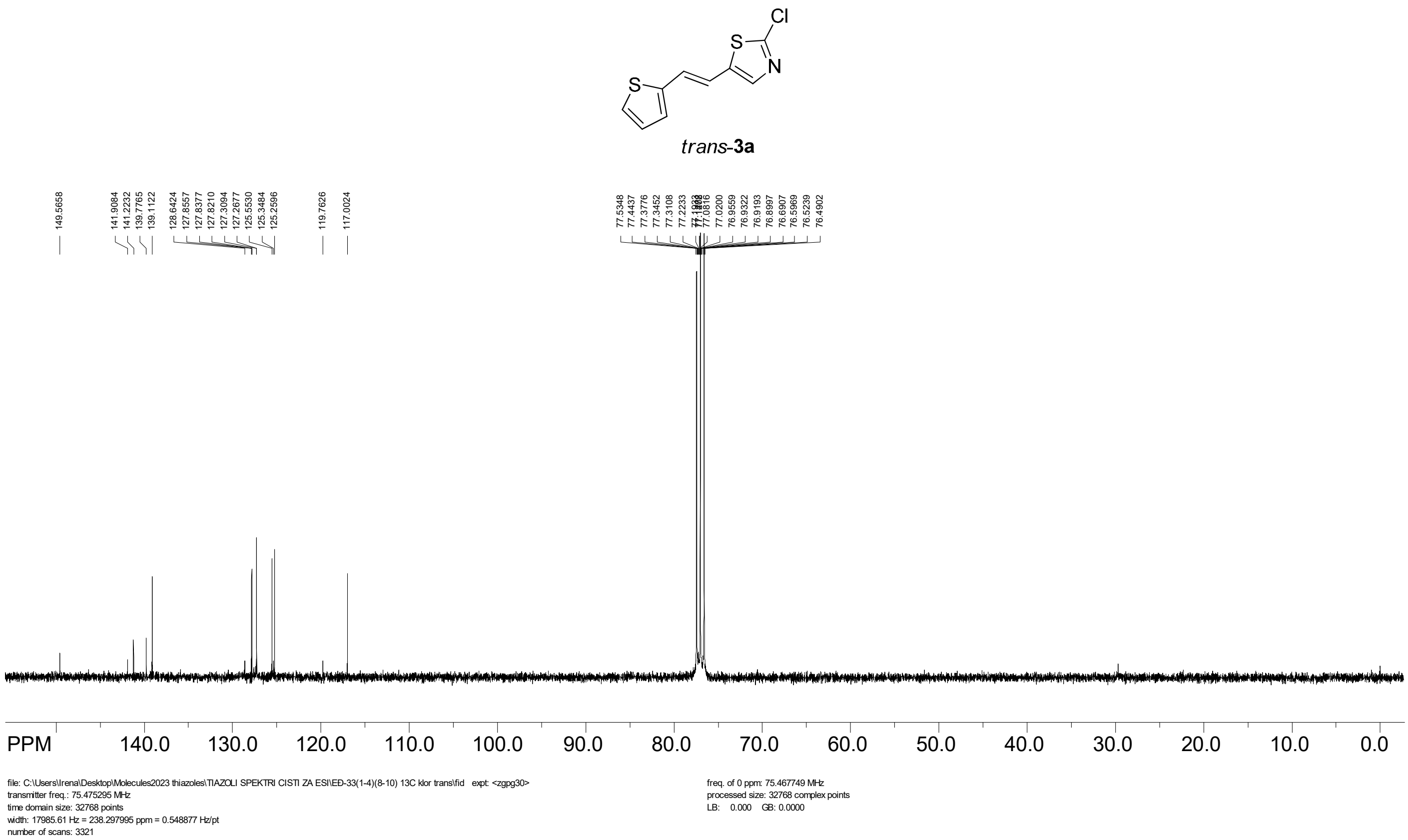

Figure S19. ^{13}C NMR spectrum ($CDCl_3$) of *trans*-**3a**.

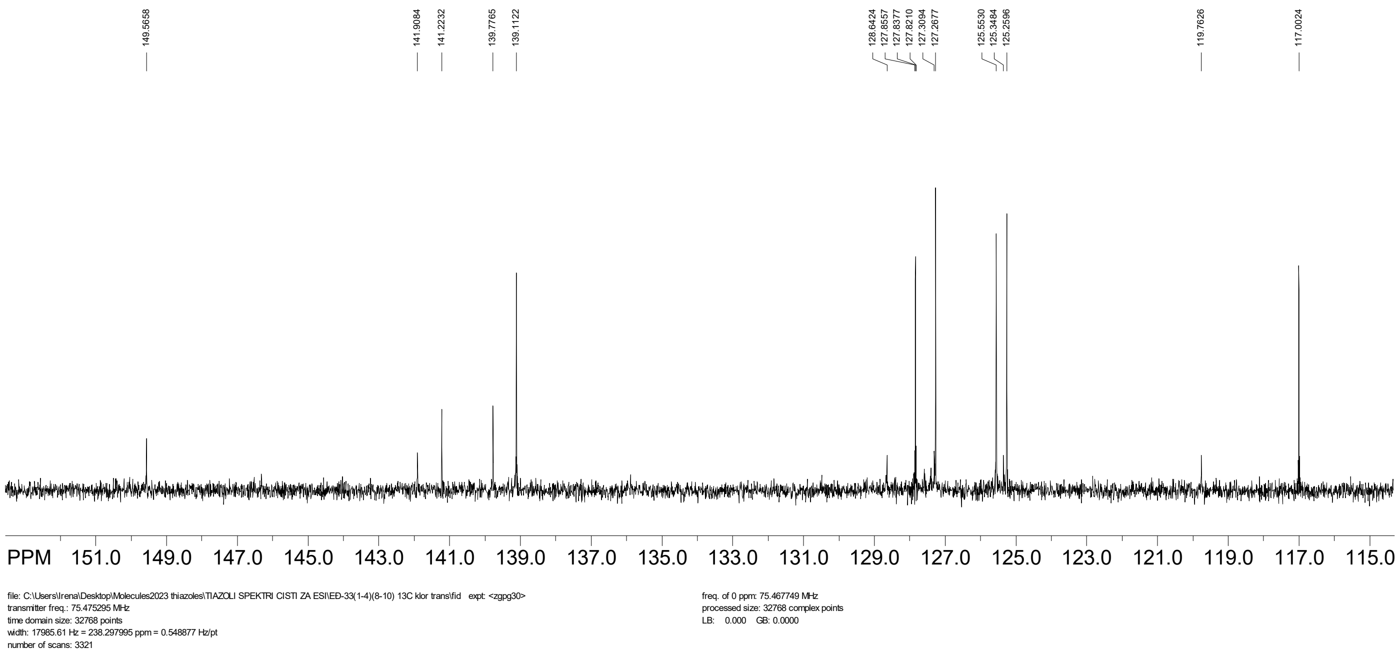

Figure S20. Aromatic part of the ^{13}C NMR spectrum ($CDCl_3$) of *trans*-**3a**.

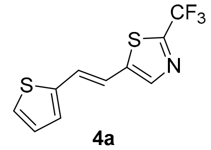

4a

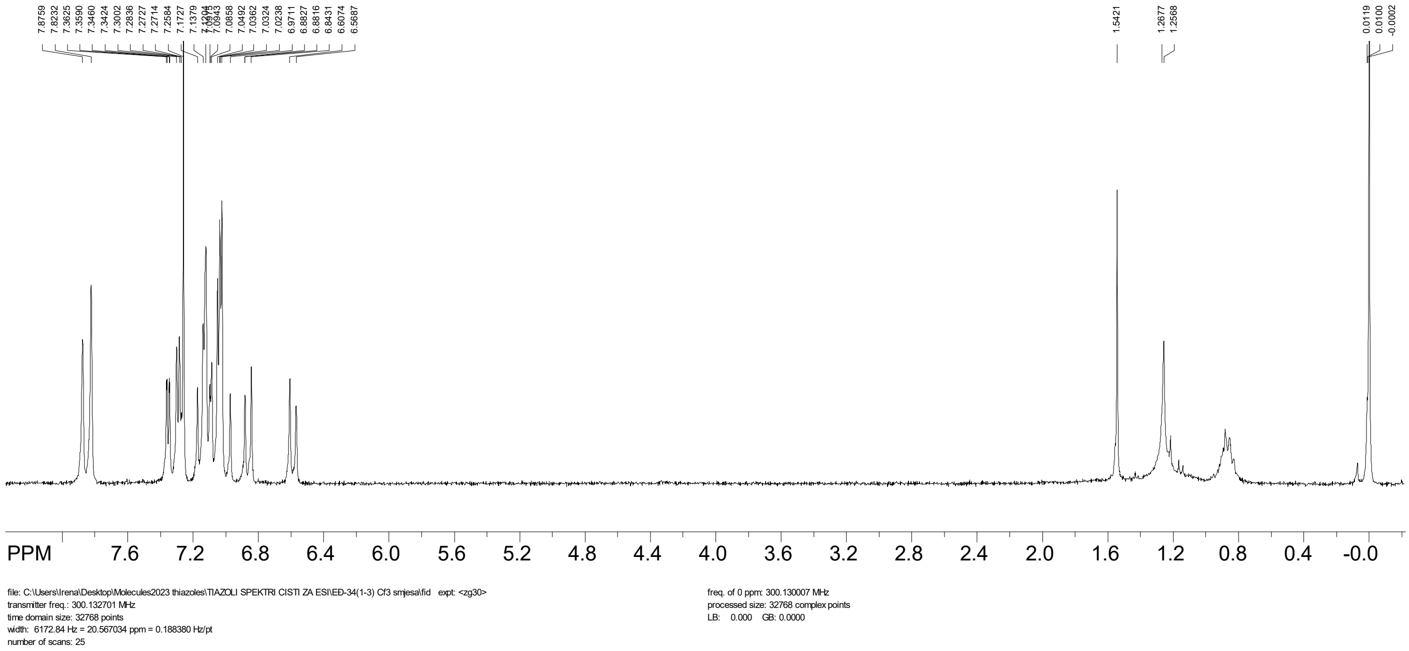

Figure S21. ^{1}H NMR spectrum ($CDCl_3$) of the pure mixture of isomers of **4a**.

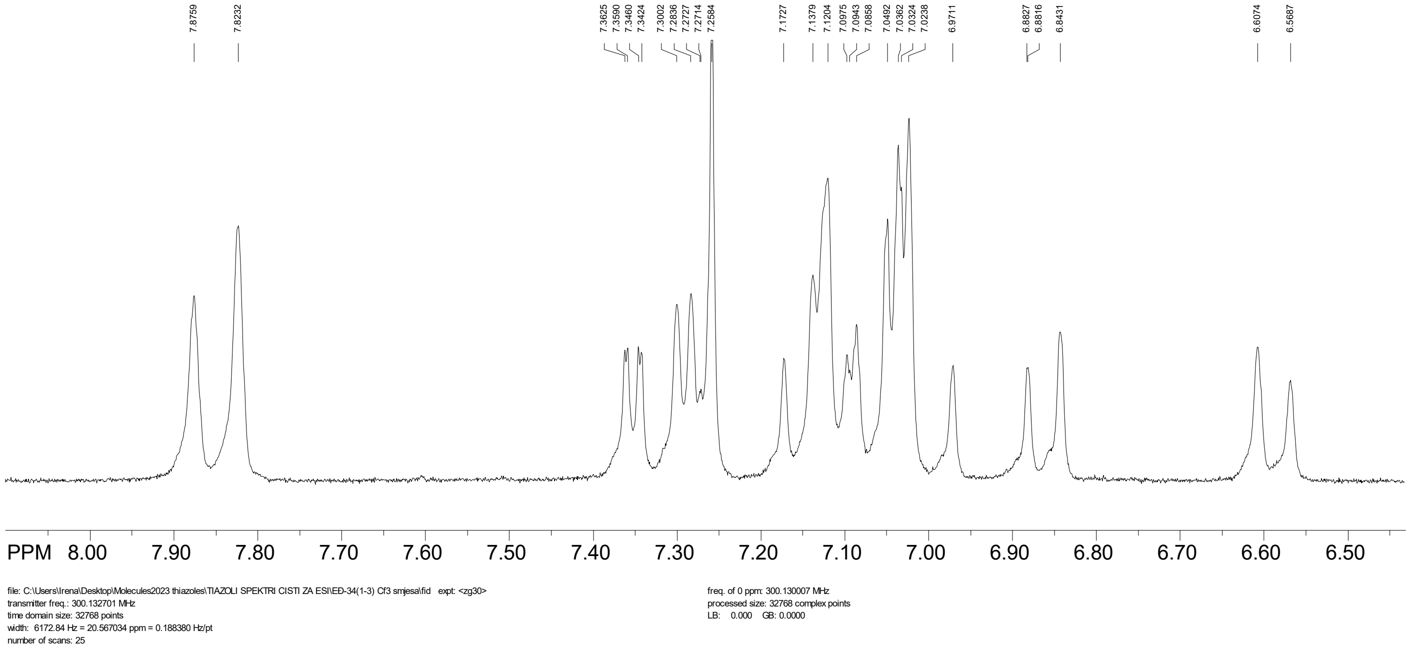

Figure S22. Aromatic part of the ^{1}H NMR spectrum ($CDCl_3$) of the pure mixture of isomers of **4a**.

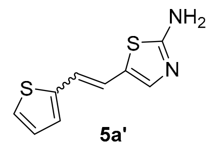

5a'

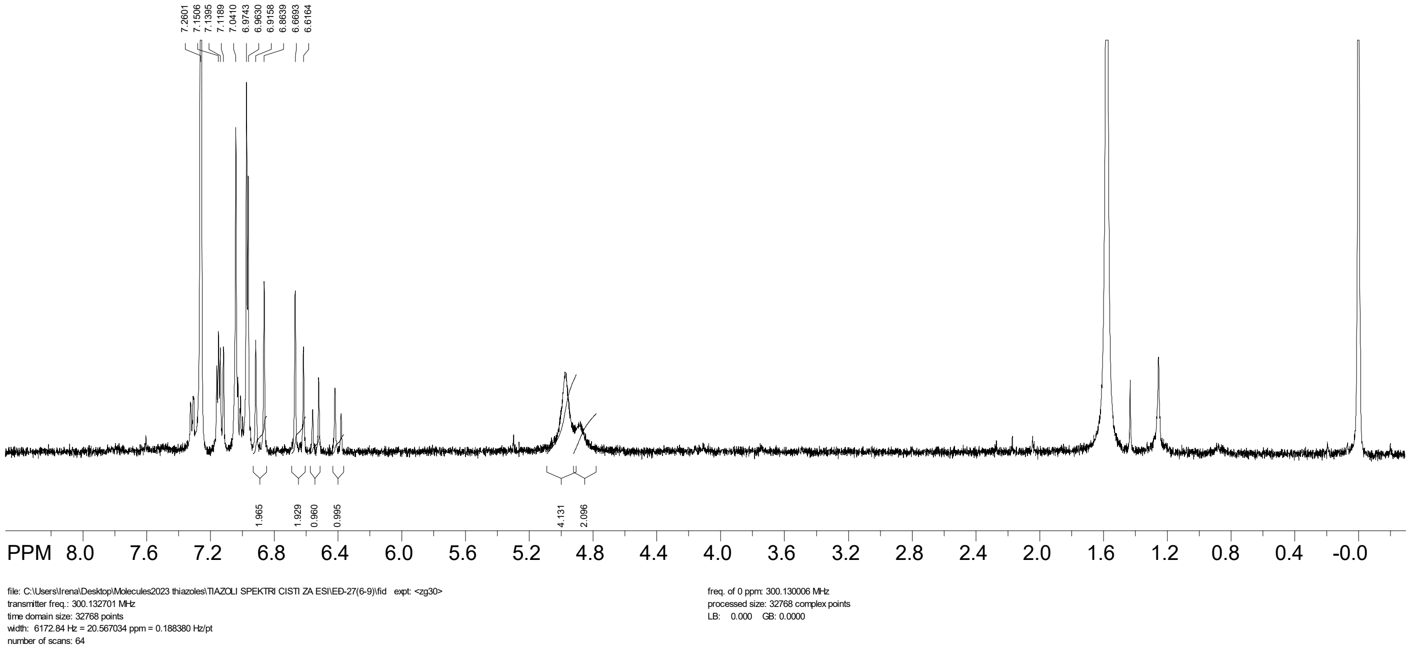

Figure S23. ^{1}H NMR spectrum ($CDCl_3$) of the pure mixture of isomers of **5a**'.

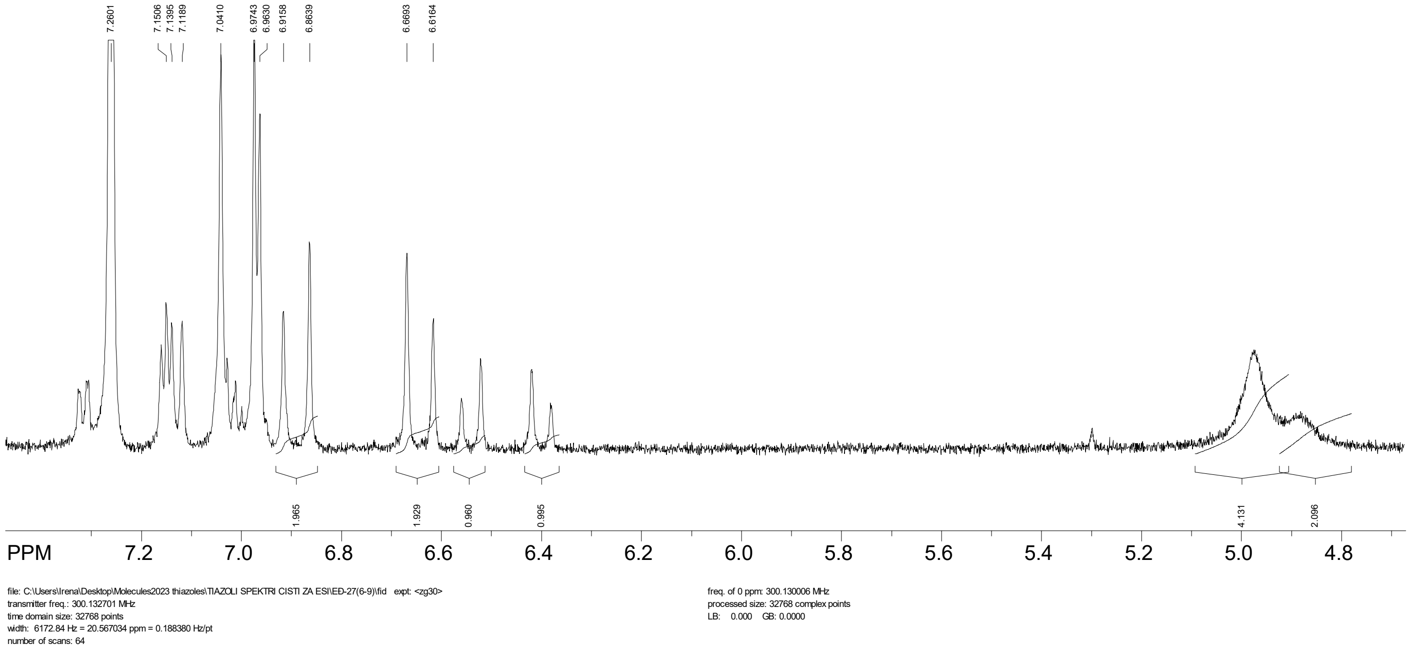

Figure S24. Aromatic part of the ^{1}H NMR spectrum ($CDCl_3$) of the pure mixture of isomers of **5a**'.

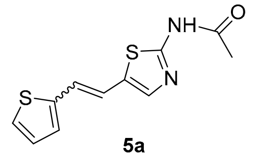

5a

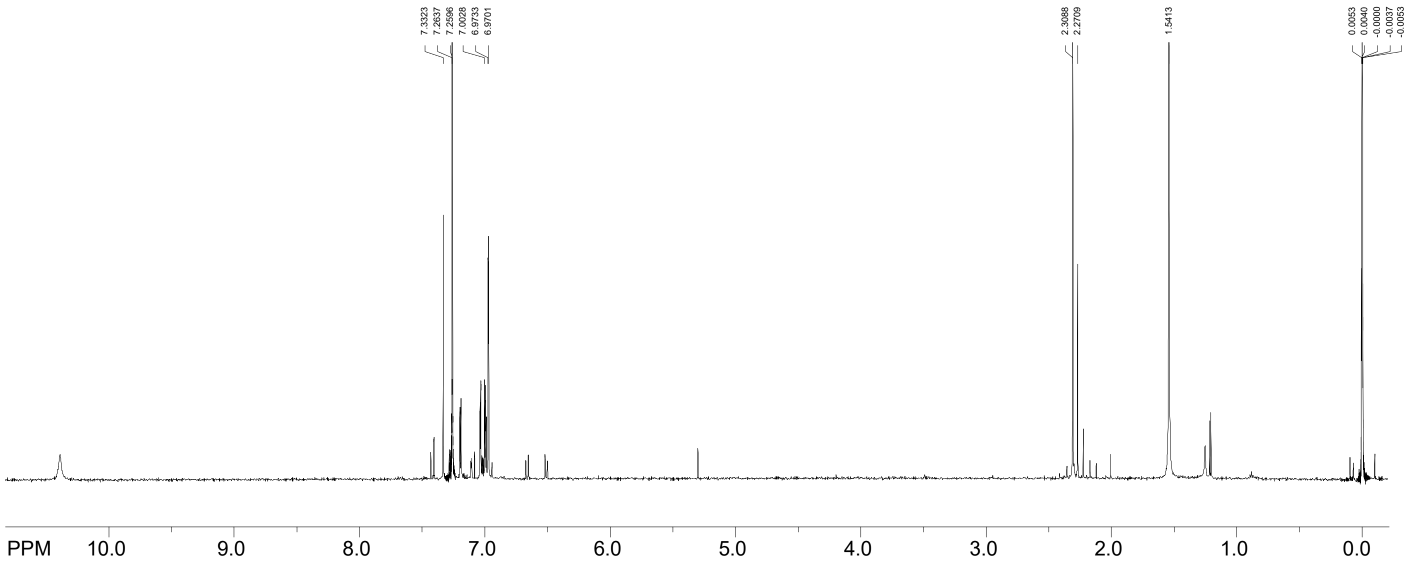

file: C:\Users\Irena\Desktop\Molecules2023 thiazoles\TIAZOLI SPEKTRI CISTI ZA ESI\ED-43(1) amidni smjesa pocetnih\fid expt: <zg30>
transmitter freq.: 600.135401 MHz
time domain size: 32768 points
width: 12019.23 Hz = 20.027532 ppm = 0.366798 Hz/pt
number of scans: 64

freq. of 0 ppm: 600.130012 MHz
processed size: 32768 complex points
LB: 0.000 GB: 0.0000

Figure S25. ^{1}H NMR spectrum ($CDCl_3$) of the pure mixture of isomers of **5a**.

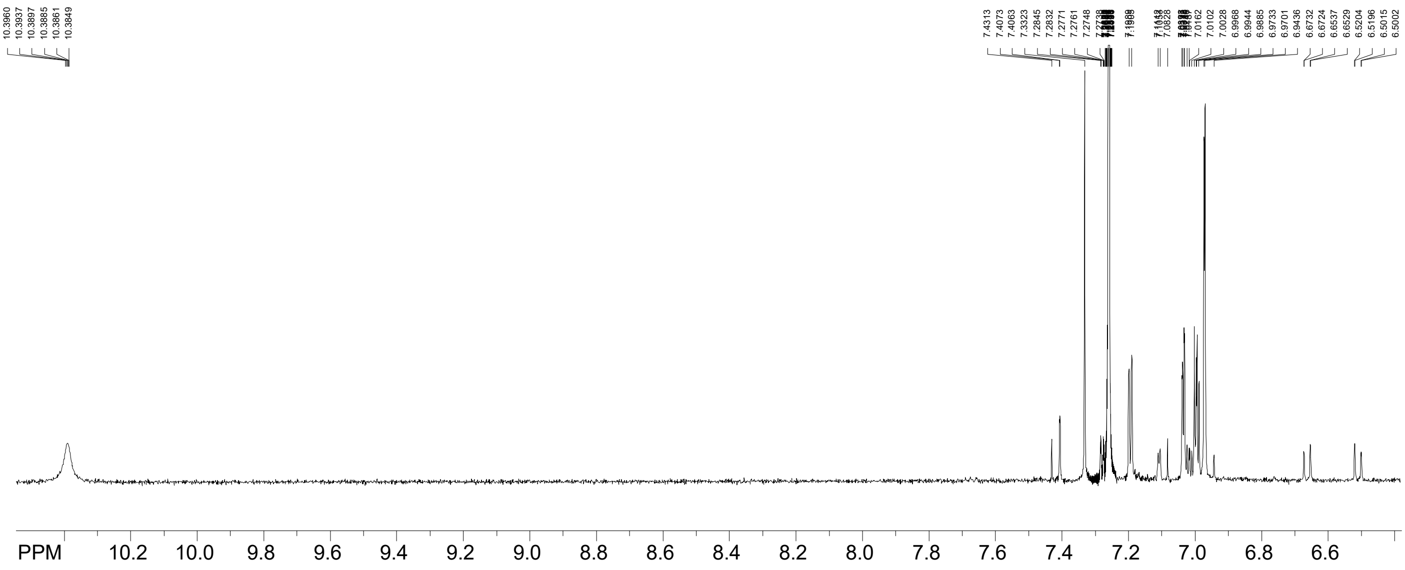

file: C:\Users\Irena\Desktop\Molecules2023 thiazoles\TIAZOLI SPEKTRI CISTI ZA ESI\ED-43(1) amidni smjesa pocetnih\fid expt: <zg30>
transmitter freq.: 600.135401 MHz
time domain size: 32768 points
width: 12019.23 Hz = 20.027532 ppm = 0.366798 Hz/pt
number of scans: 64

freq. of 0 ppm: 600.130012 MHz
processed size: 32768 complex points
LB: 0.000 GB: 0.0000

Figure S26. Aromatic part of the ^{1}H NMR spectrum ($CDCl_3$) of the pure mixture of isomers of **5a**.

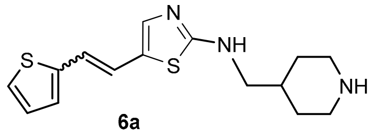

6a

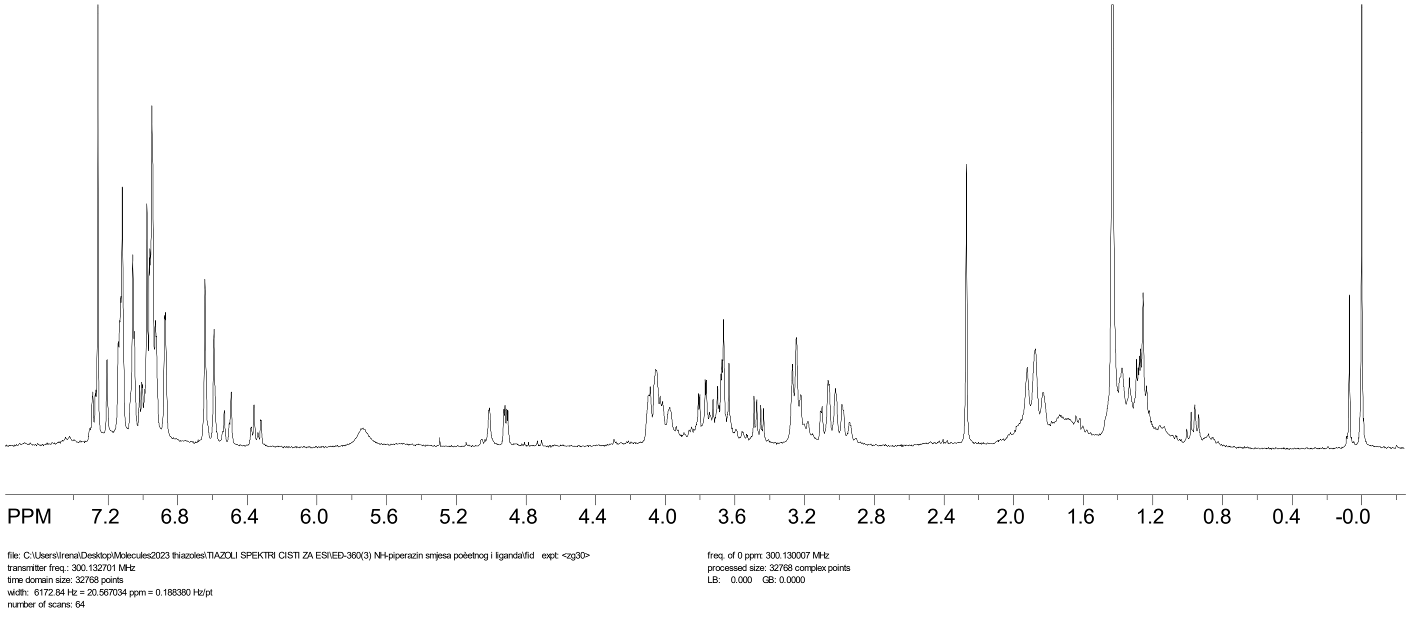

Figure S27. ^{1}H NMR spectrum ($CDCl_3$) of the mixture of isomers of **6a** and the catalyst.

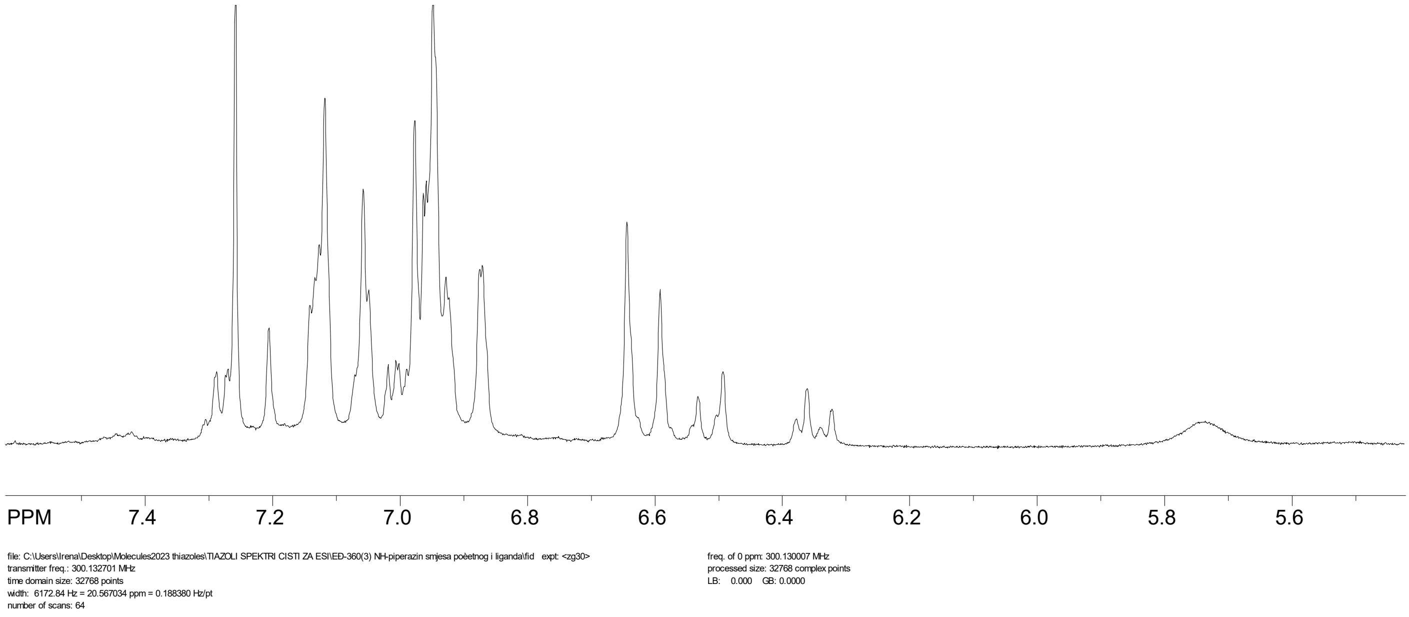

Figure S28. Aromatic part of the ^{1}H NMR spectrum ($CDCl_3$) of the mixture of isomers of **6a** and the catalyst.

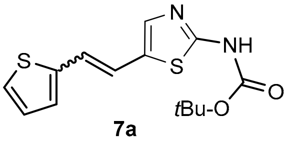

7a

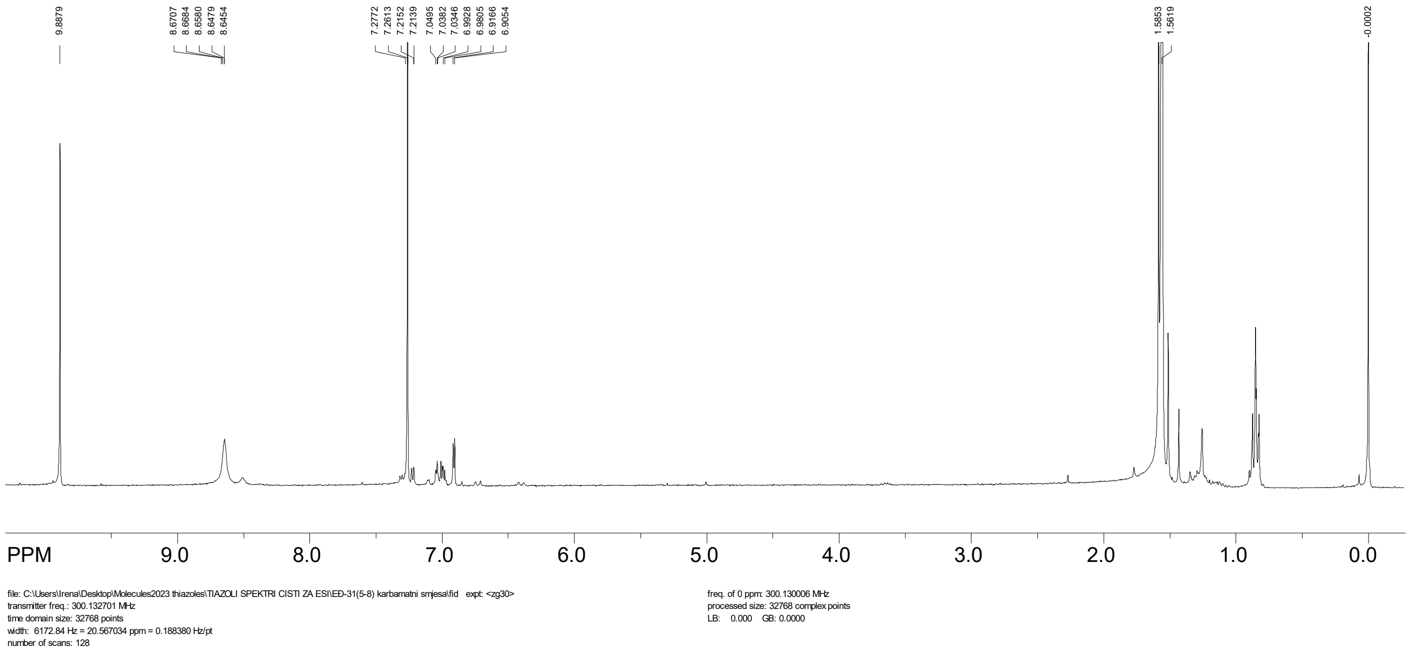

Figure S29. ^{1}H NMR spectrum ($CDCl_3$) of the mixture of isomers of **7a** and starting aldehyde.

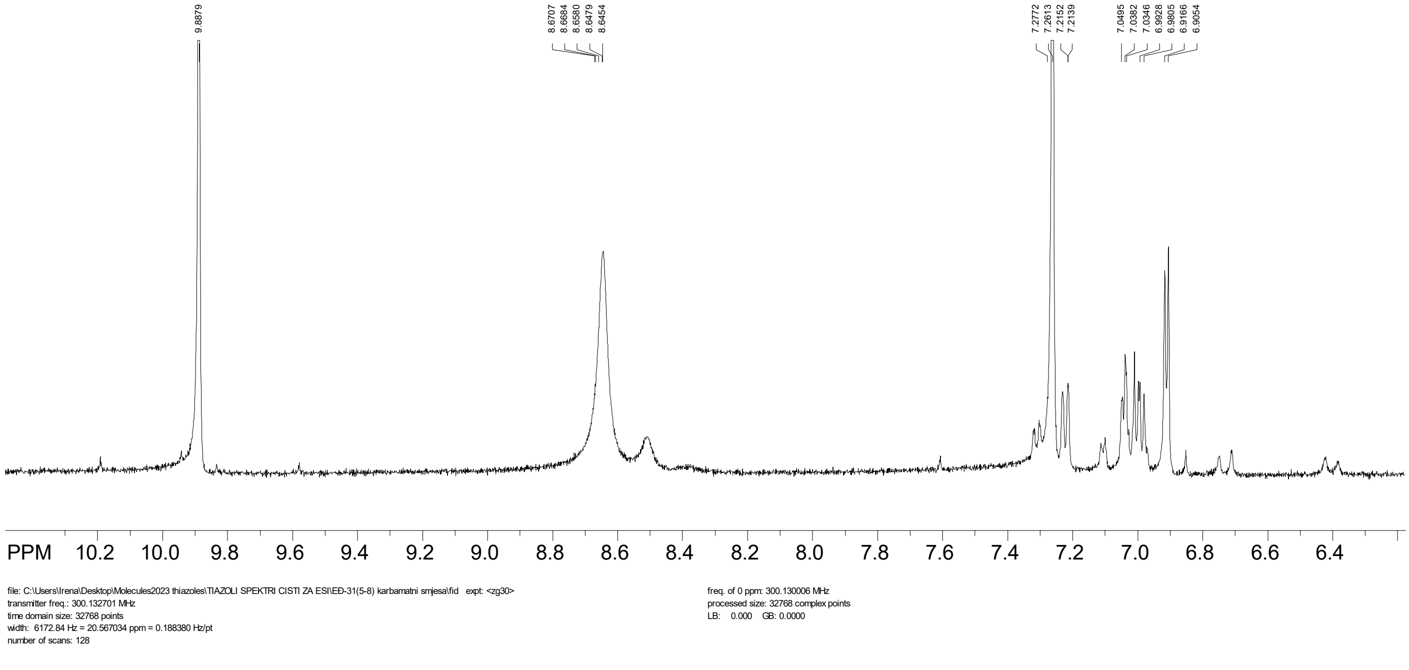

Figure S30. Aromatic part of the ^{1}H NMR spectrum ($CDCl_3$) of the mixture of isomers of **7a** and starting aldehyde.

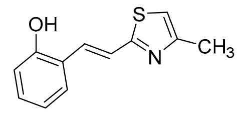

trans-**8a**

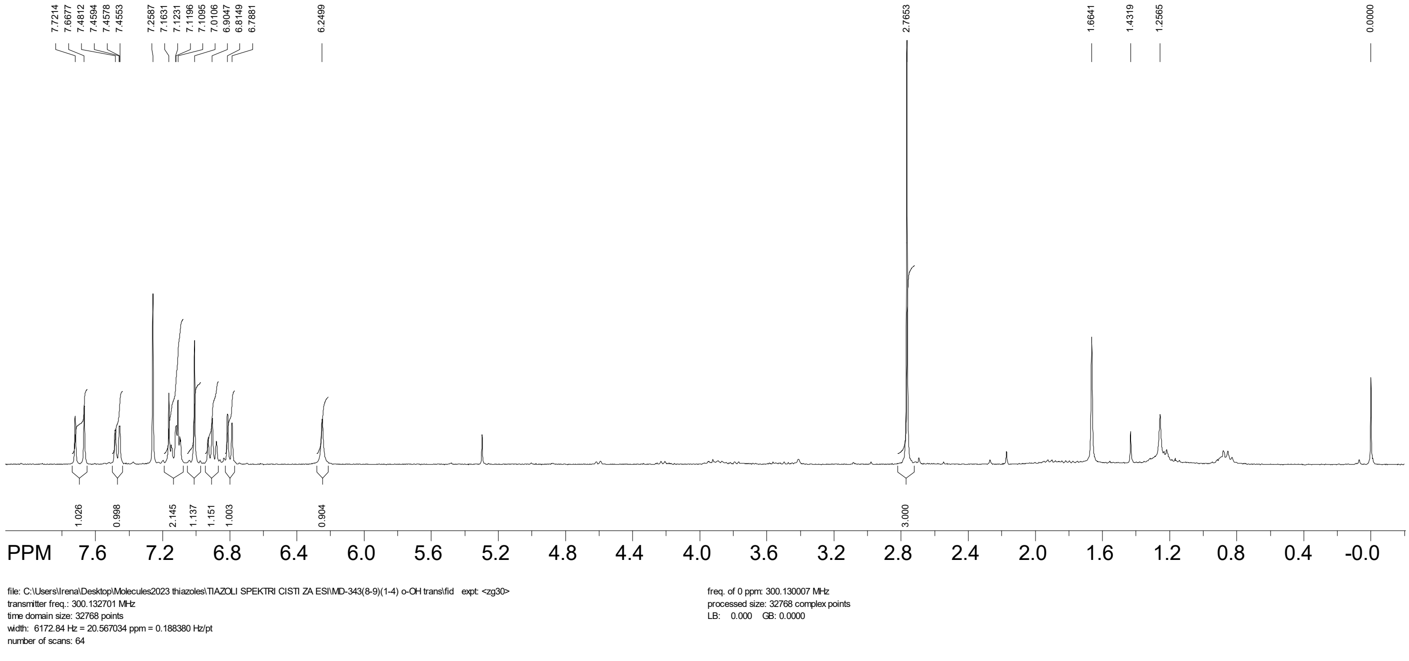

Figure S31. ^{1}H NMR spectrum ($CDCl_3$) of *trans*-**8a**.

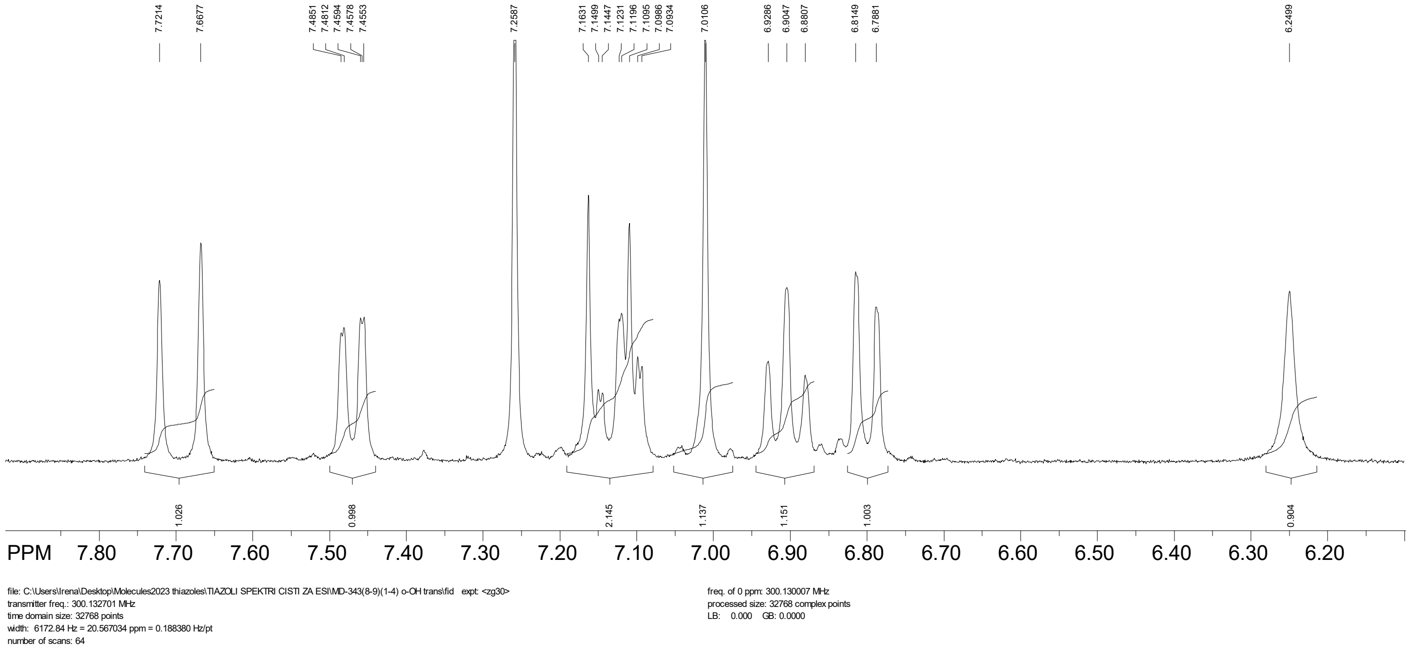

Figure S32. Aromatic part of the ^{1}H NMR spectrum ($CDCl_3$) of *trans*-**8a**.

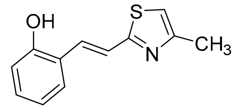

trans-**8a**

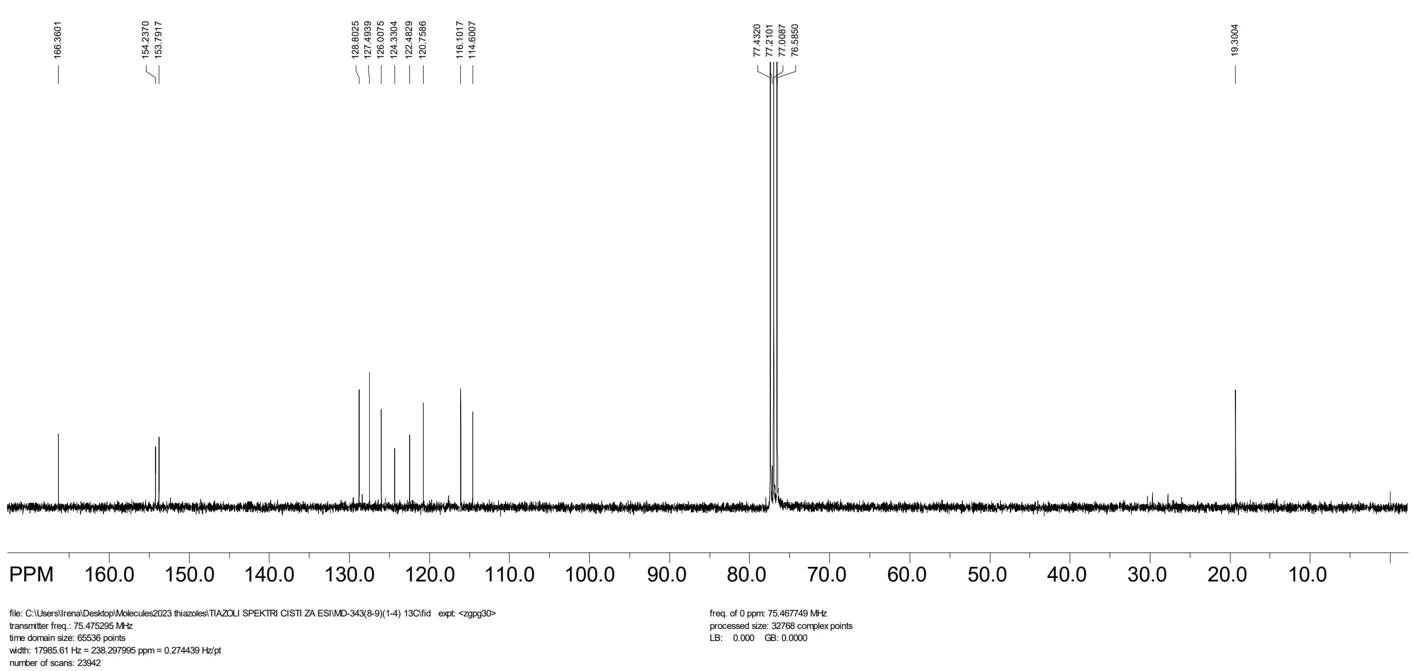

Figure S33. ^{13}C NMR spectrum ($CDCl_3$) of *trans*-**8a**.

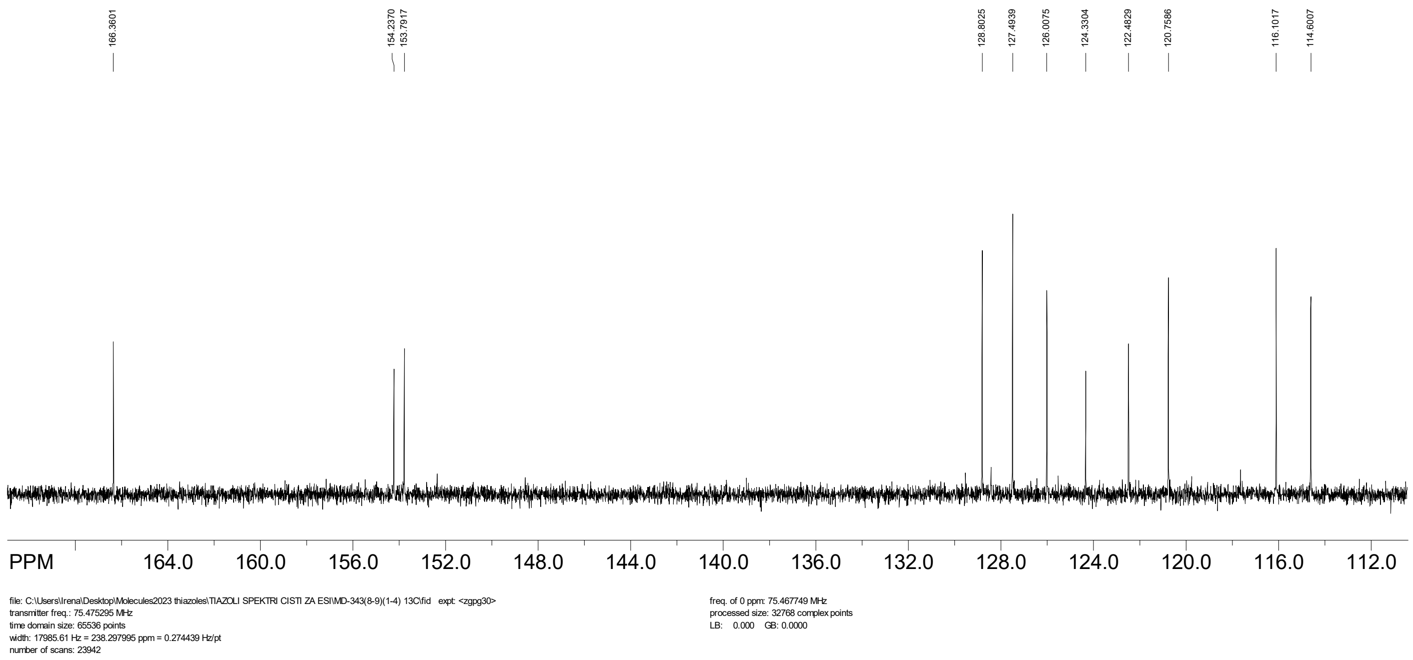

Figure S34. Aromatic part of the ^{13}C NMR spectrum ($CDCl_3$) of *trans*-**8a**.

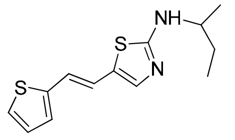

trans-**9a**

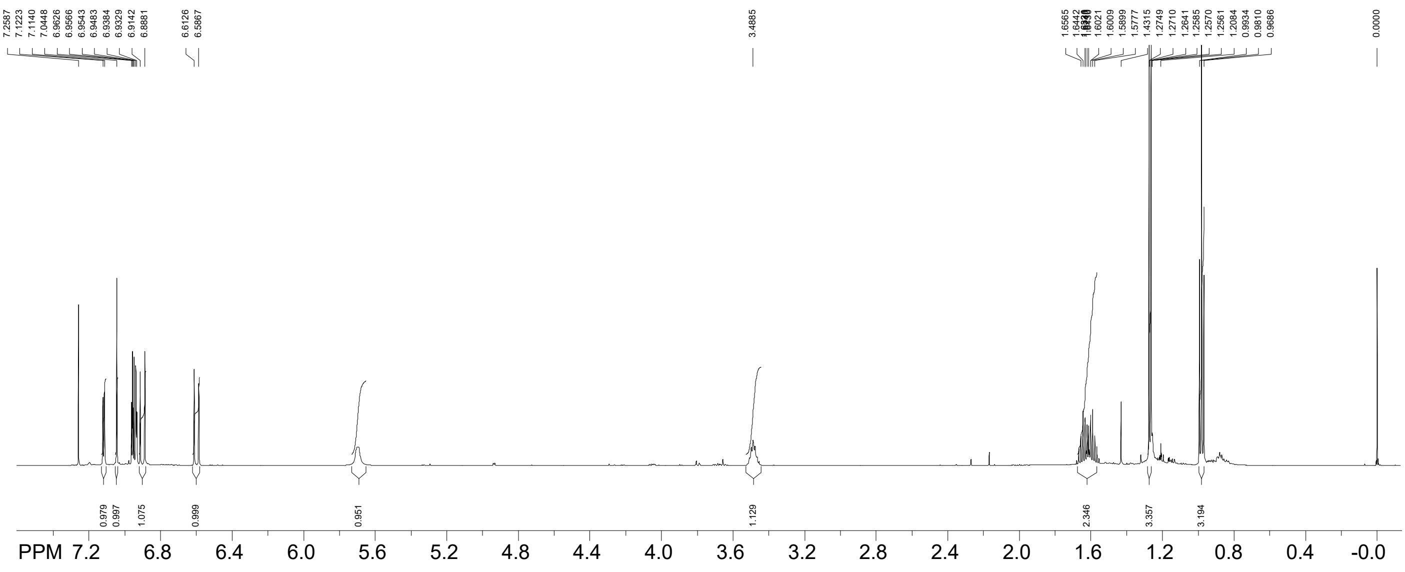

Figure S35. ^{1}H NMR spectrum ($CDCl_3$) of *trans*-**9a**.

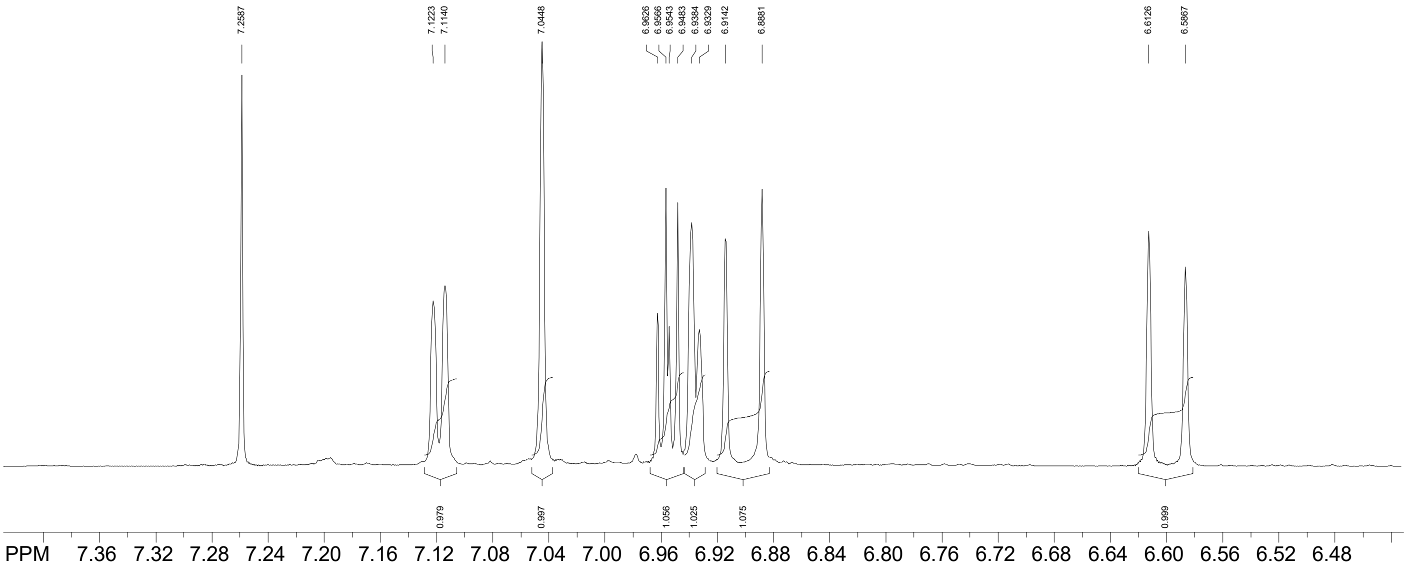

Figure S36. Aromatic part of the ^{1}H NMR spectrum ($CDCl_3$) of *trans*-**9a**.

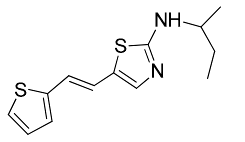

trans-**9a**

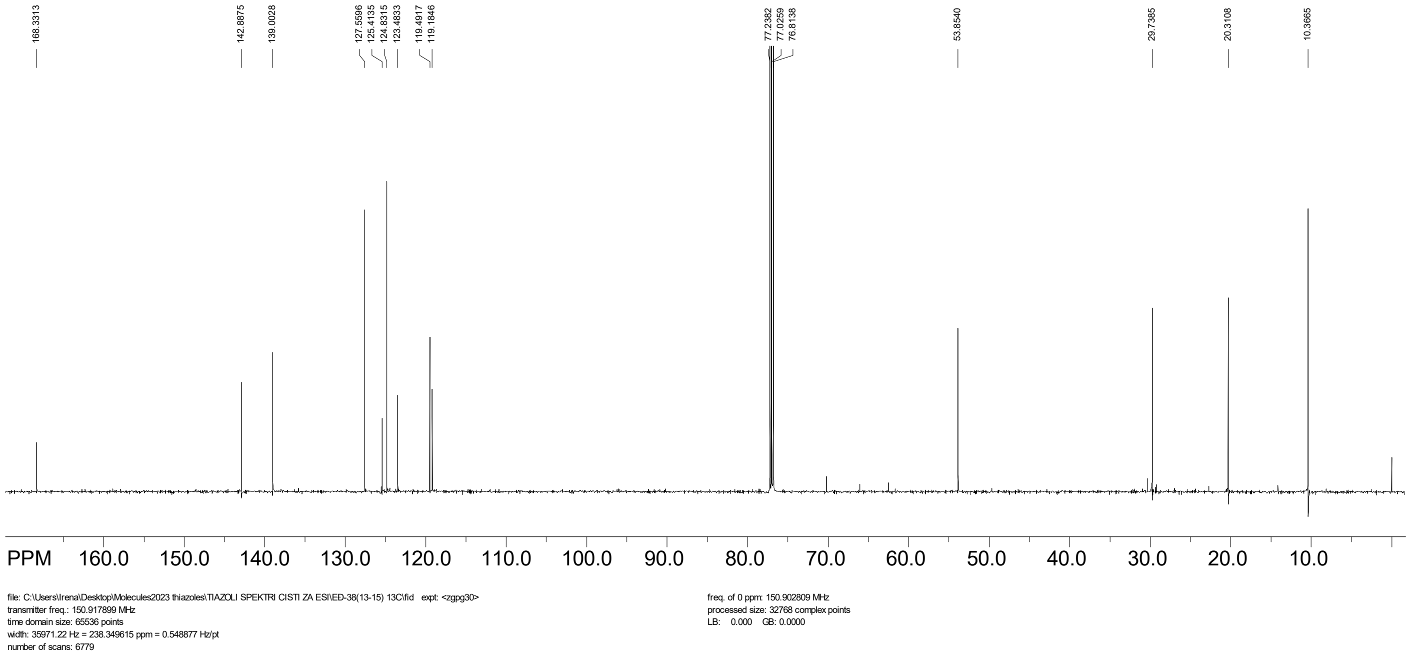

Figure S37. ^{13}C NMR spectrum ($CDCl_3$) of *trans*-**9a**.

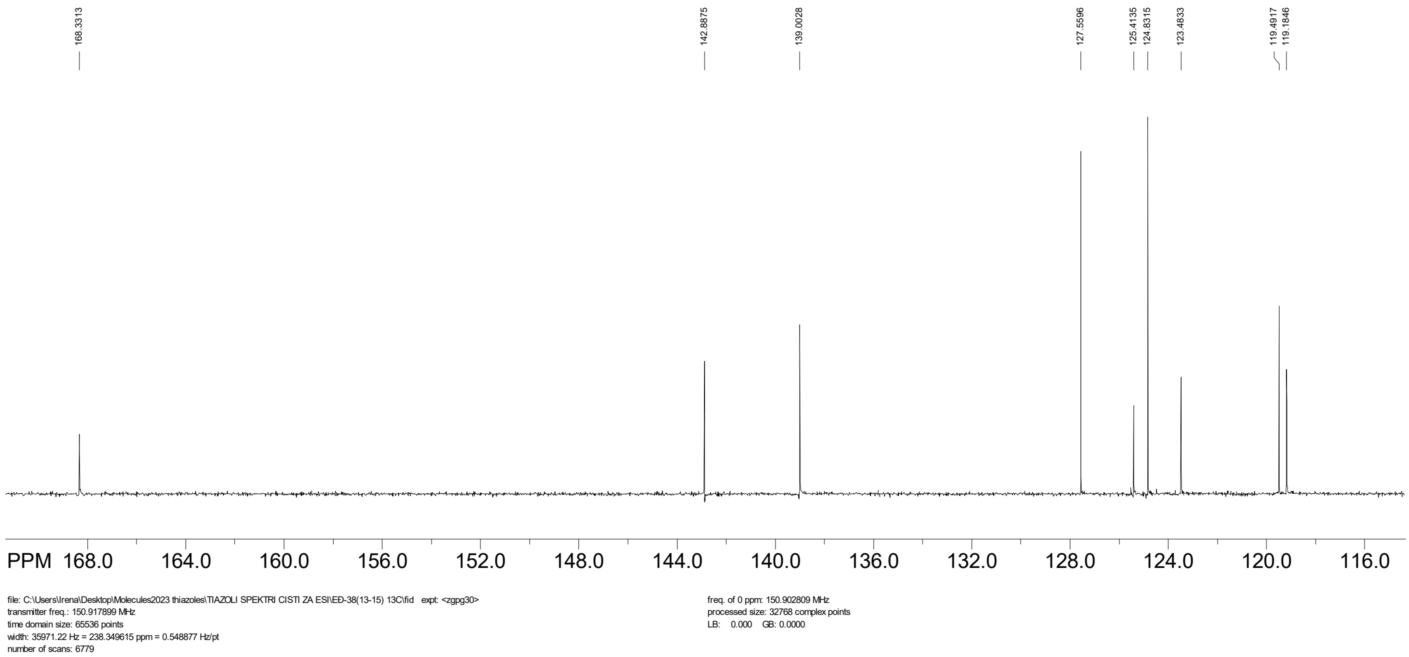

Figure S38. Aromatic part of the ^{13}C NMR spectrum ($CDCl_3$) of *trans*-**9a**.

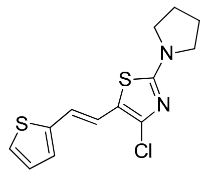

trans-**10a**

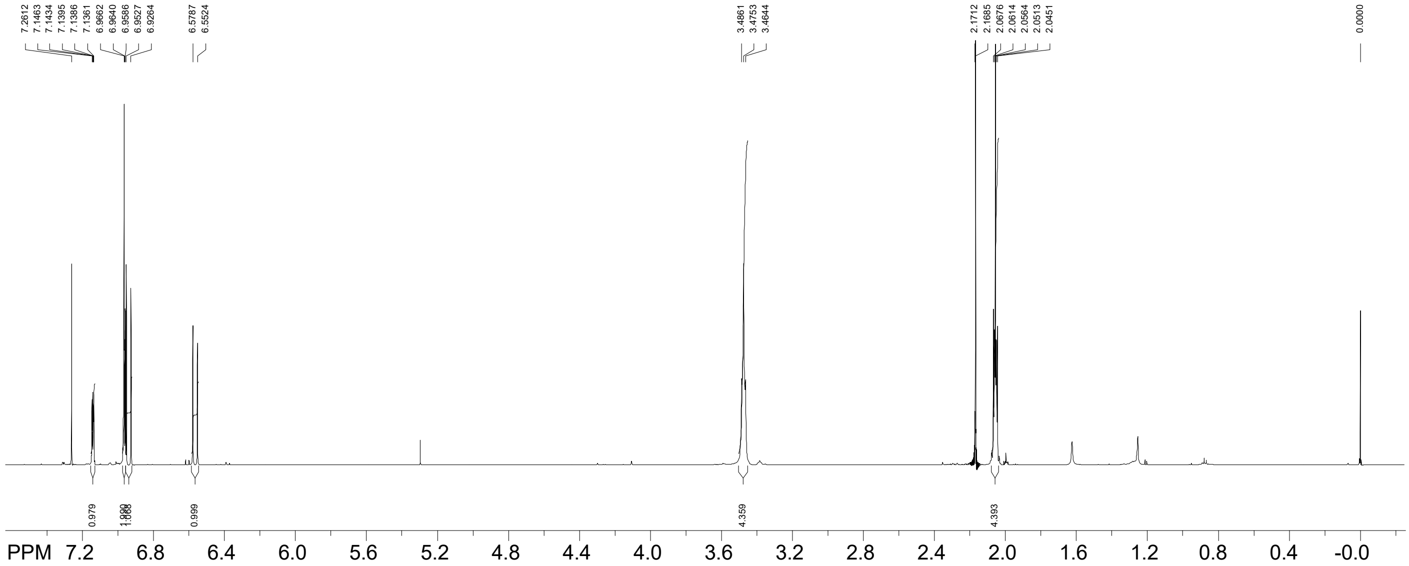

file: C:\Users\Irena\Desktop\Molecules2023 thiazoles\TIAZOLI SPEKTRI CISTI ZA ESI\ED-32(1-5)(7) pirolidin trans\fid expt: <zg30>
transmitter freq.: 600.135401 MHz
time domain size: 32768 points
width: 12019.23 Hz = 20.027532 ppm = 0.366798 Hz/pt
number of scans: 25

freq. of 0 ppm: 600.130010 MHz
processed size: 32768 complex points
LB: 0.000 GB: 0.0000

Figure S39. ^{1}H NMR spectrum ($CDCl_3$) of *trans*-**10a**.

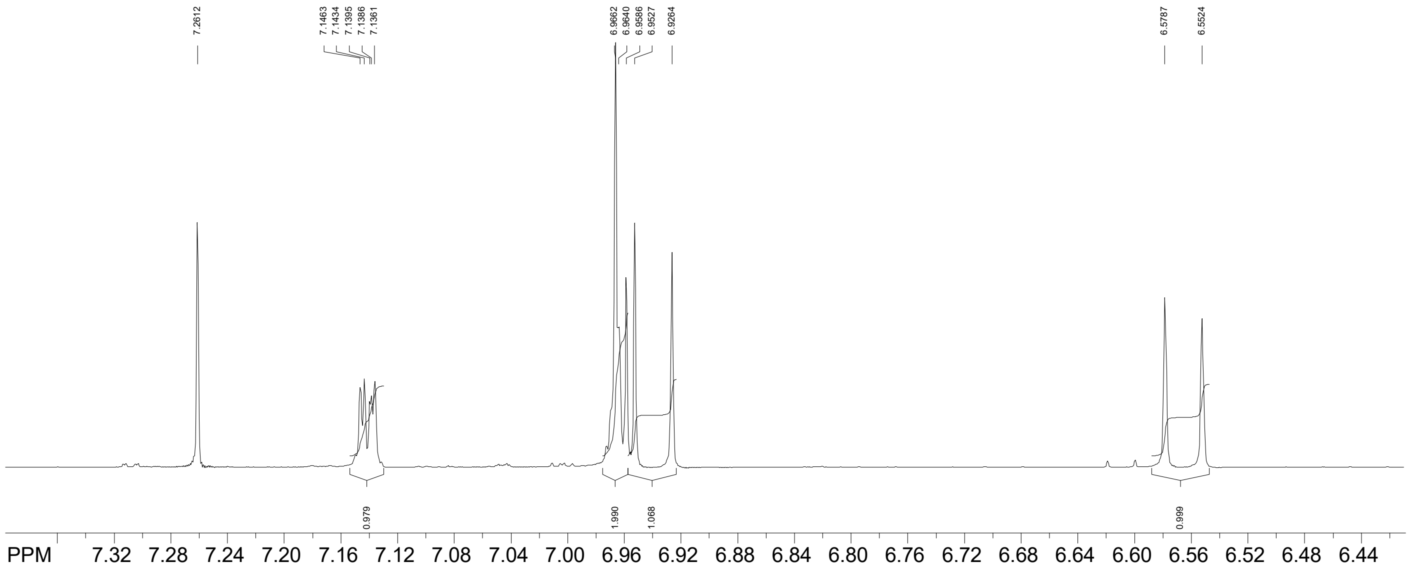

file: C:\Users\Irena\Desktop\Molecules2023 thiazoles\TIAZOLI SPEKTRI CISTI ZA ESI\ED-32(1-5)(7) pirolidin trans\fid expt: <zg30>
transmitter freq.: 600.135401 MHz
time domain size: 32768 points
width: 12019.23 Hz = 20.027532 ppm = 0.366798 Hz/pt
number of scans: 25

freq. of 0 ppm: 600.130010 MHz
processed size: 32768 complex points
LB: 0.000 GB: 0.0000

Figure S40. Aromatic part of the ^{1}H NMR spectrum ($CDCl_3$) of *trans*-**10a**.

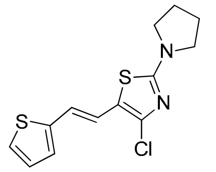

trans-**10a**

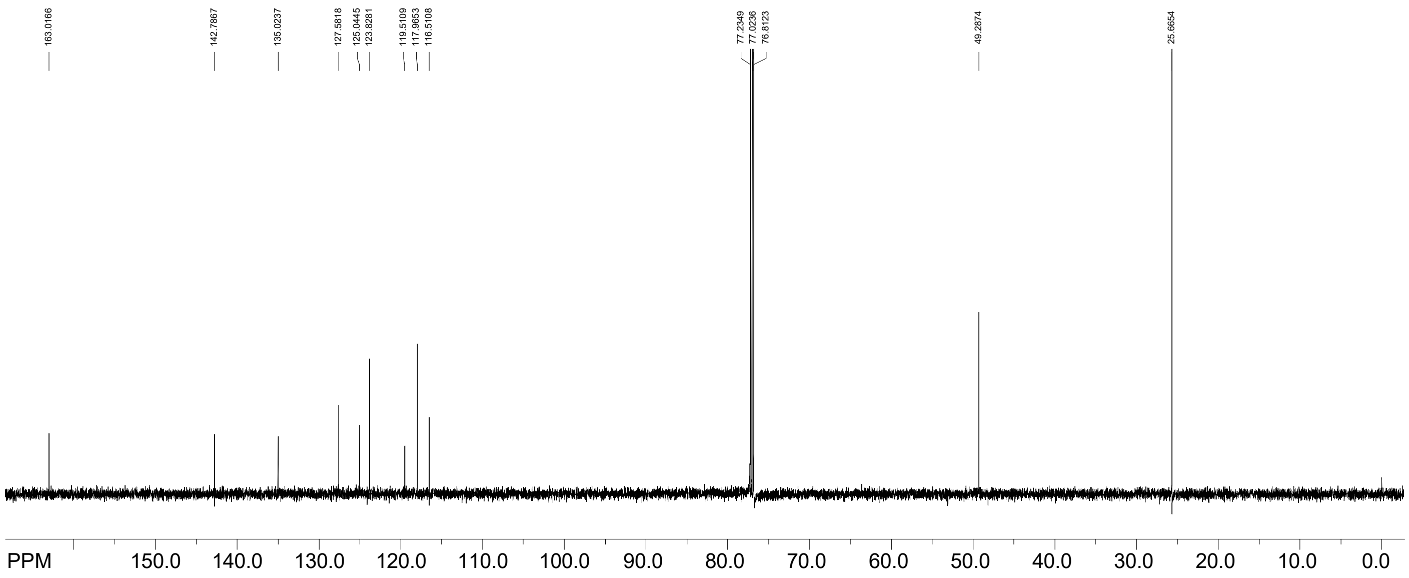

file: C:\Users\Irena\Desktop\Molecules2023 thiazoles\TIAZOLI SPEKTRI CISTI ZA ESI\ED-32(1-5)(7) 13C\fid expt: <zgpg30>
transmitter freq.: 150.917899 MHz
time domain size: 65536 points
width: 35971.22 Hz = 238.349615 ppm = 0.548877 Hz/pt
number of scans: 374

freq. of 0 ppm: 150.902809 MHz
processed size: 32768 complex points
LB: 0.000 GB: 0.0000

Figure S41. ^{13}C NMR spectrum ($CDCl_3$) of *trans*-**10a**.

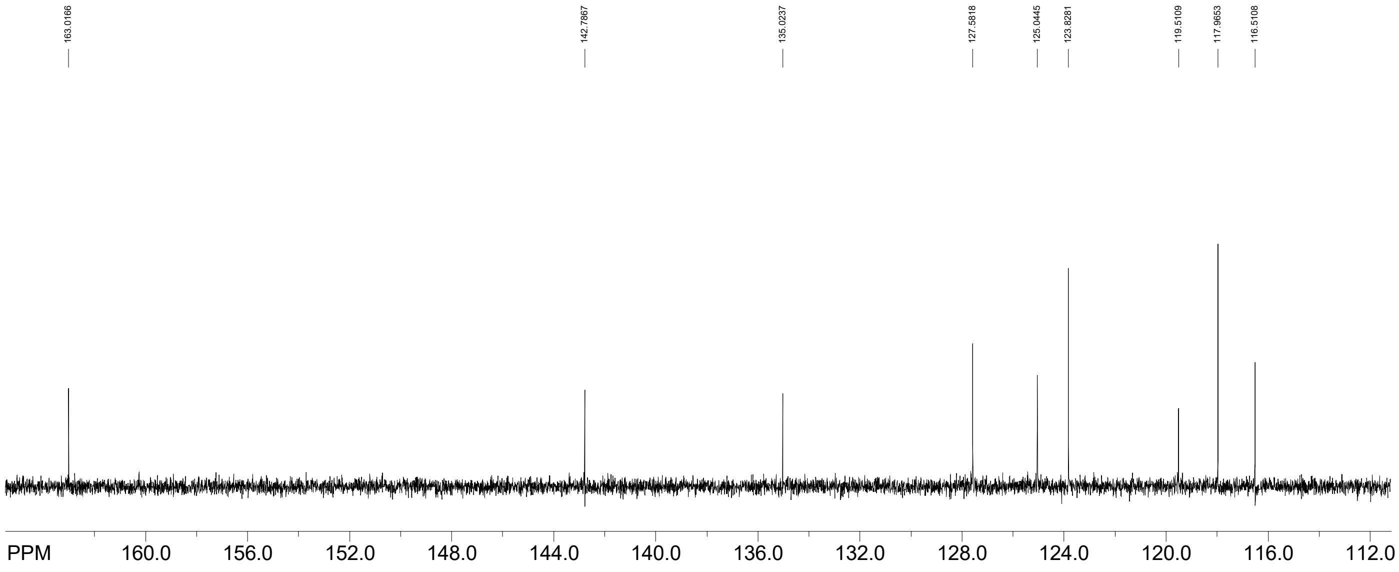

file: C:\Users\Irena\Desktop\Molecules2023 thiazoles\TIAZOLI SPEKTRI CISTI ZA ESI\ED-32(1-5)(7) 13C\fid expt: <zgpg30>
transmitter freq.: 150.917899 MHz
time domain size: 65536 points
width: 35971.22 Hz = 238.349615 ppm = 0.548877 Hz/pt
number of scans: 374

freq. of 0 ppm: 150.902809 MHz
processed size: 32768 complex points
LB: 0.000 GB: 0.0000

Figure S42. Aromatic part of the ^{13}C NMR spectrum ($CDCl_3$) of *trans*-**10a**.

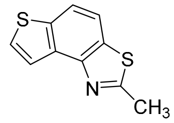

1

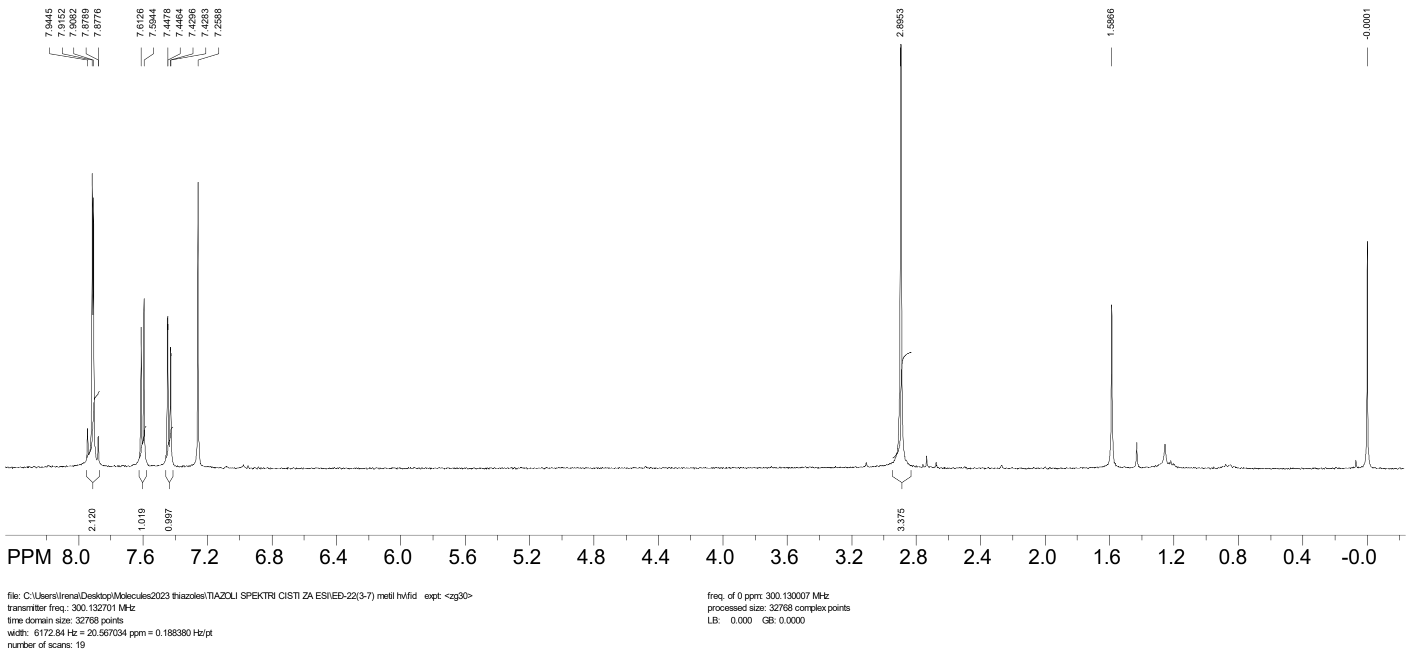

Figure S43. ^{1}H NMR spectrum ($CDCl_3$) of **1**.

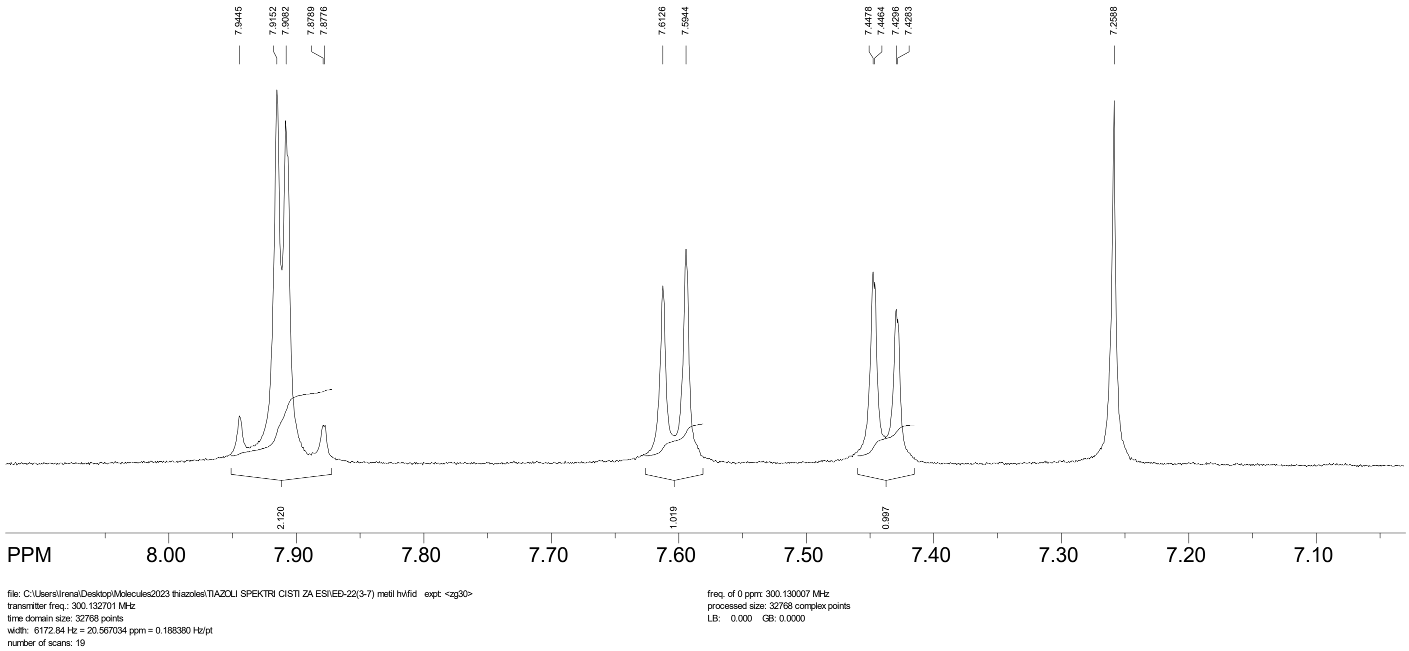

Figure S44. Aromatic part of the ^{1}H NMR spectrum ($CDCl_3$) of **1**.

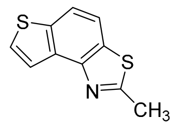

1

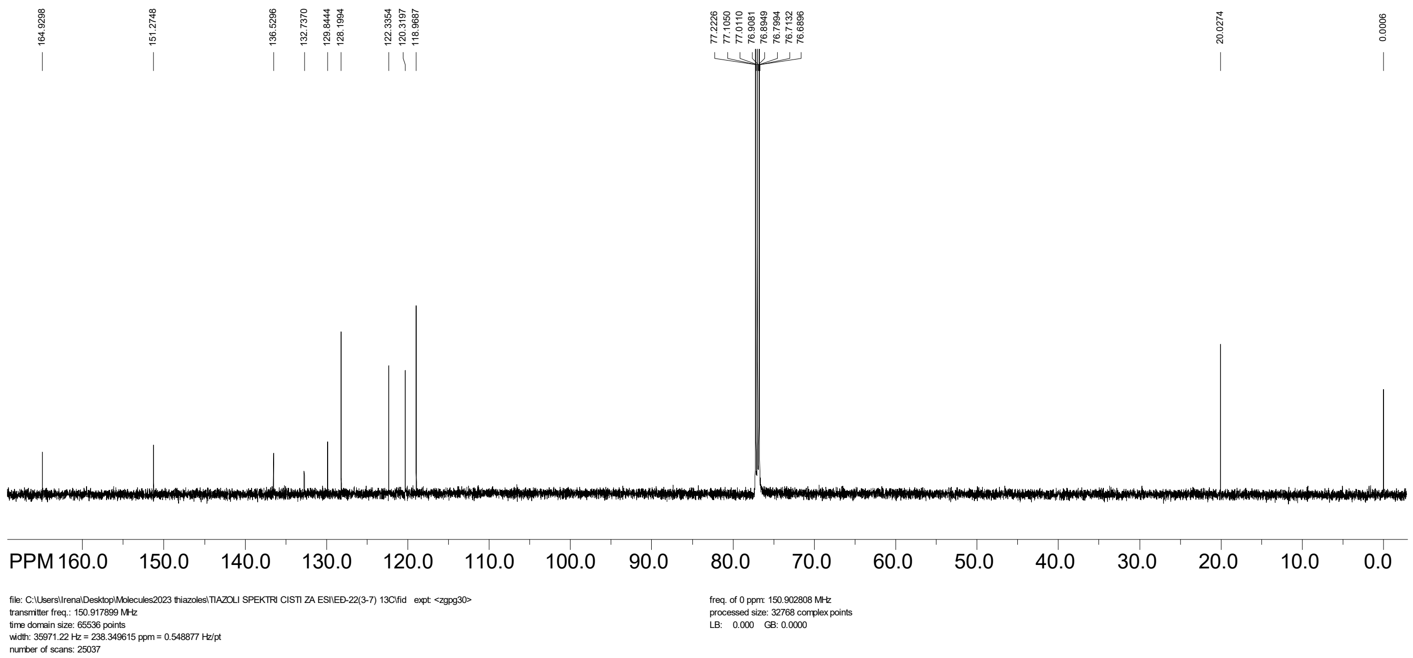

Figure S45. ^{13}C NMR spectrum ($CDCl_3$) of **1**.

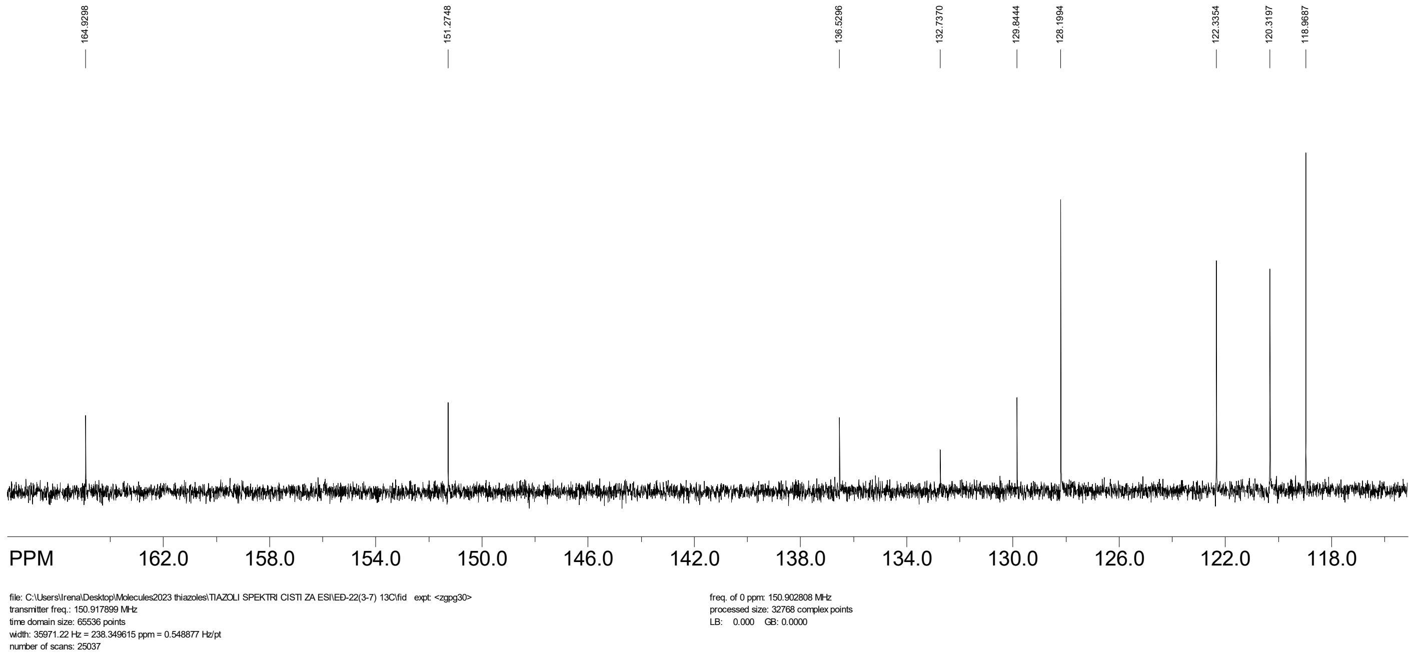

Figure S46. Aromatic part of the ^{13}C NMR spectrum ($CDCl_3$) of **1**.

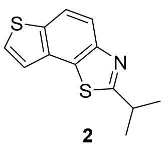

2

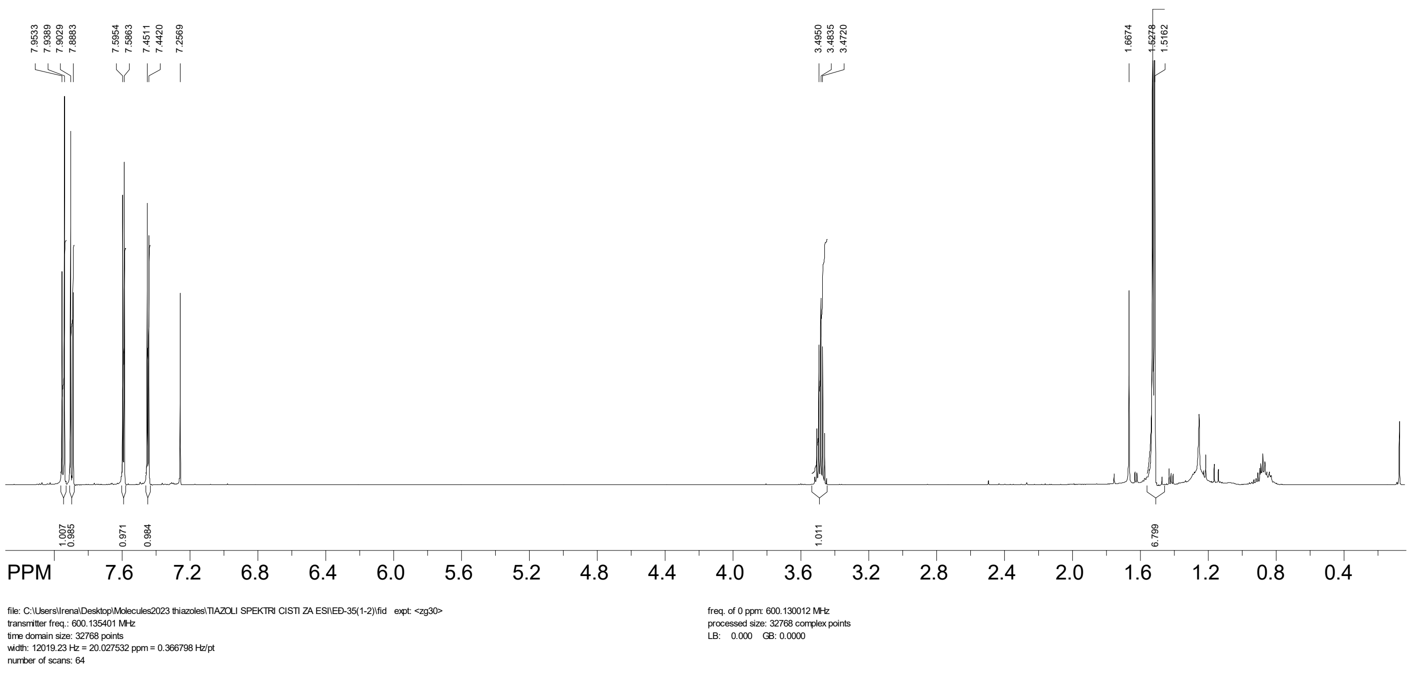

Figure S47. ^{1}H NMR spectrum ($CDCl_3$) of **2**.

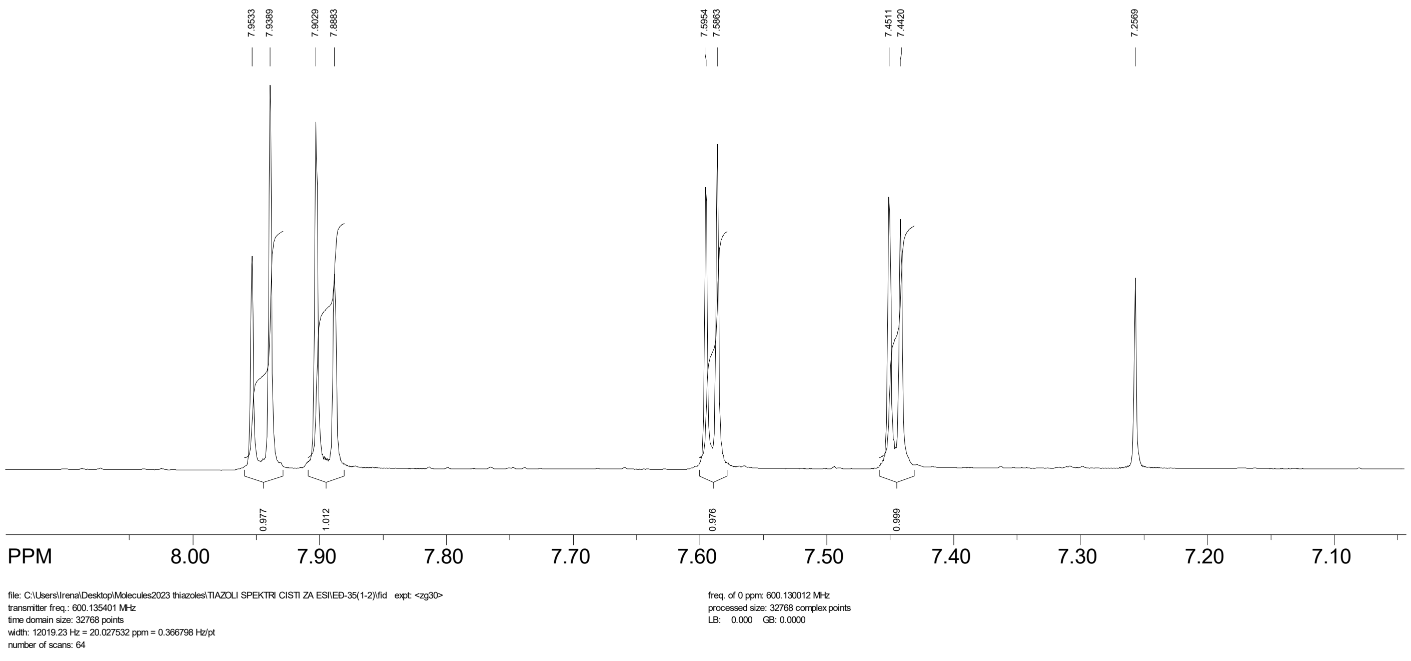

Figure S48. Aromatic part of the ^{1}H NMR spectrum ($CDCl_3$) of **2**.

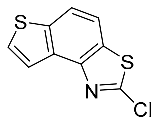

3

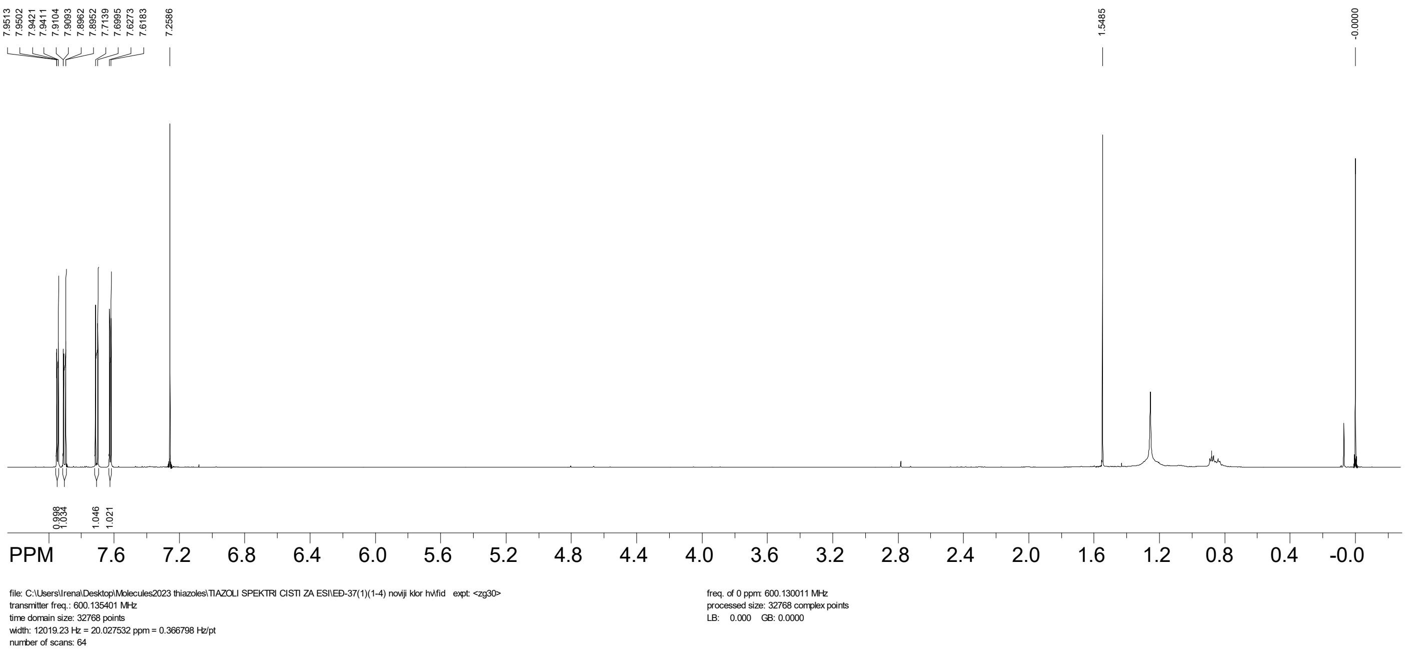

Figure S49. ^{1}H NMR spectrum ($CDCl_3$) of **3**.

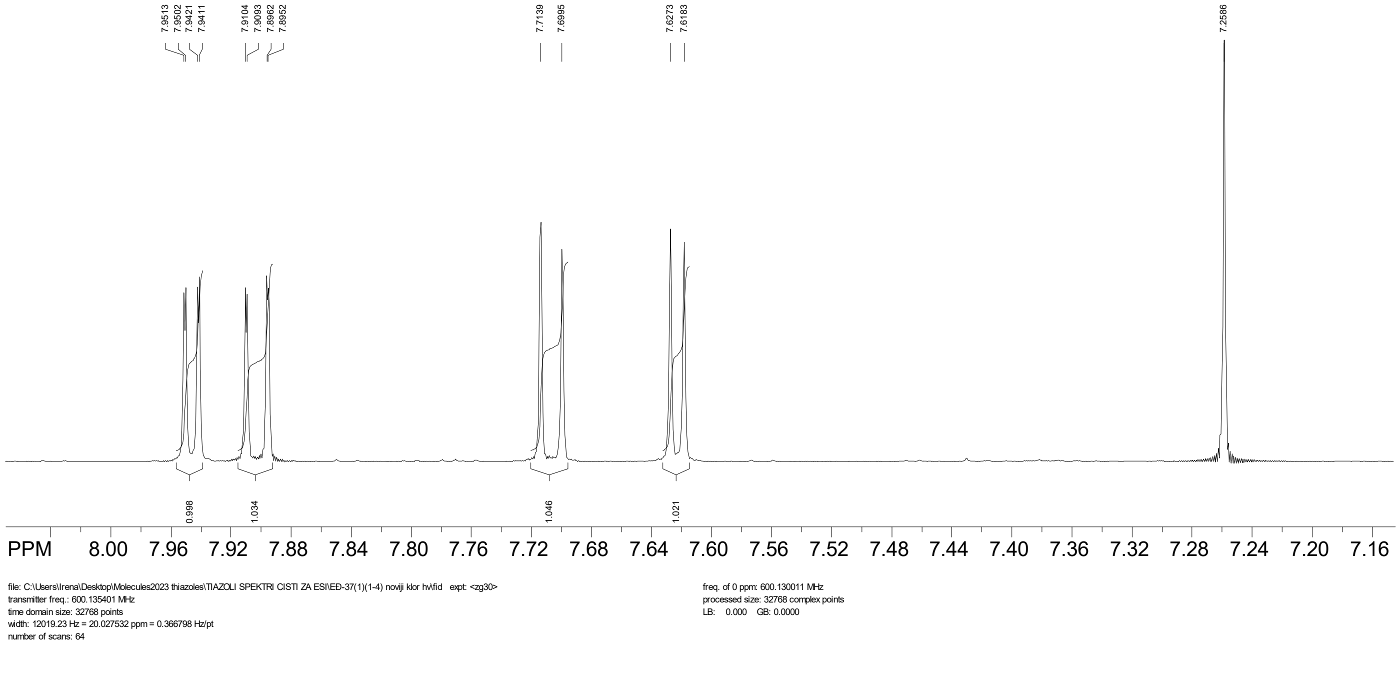

Figure S50. Aromatic part of the ^{1}H NMR spectrum ($CDCl_3$) of **3**.

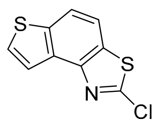

3

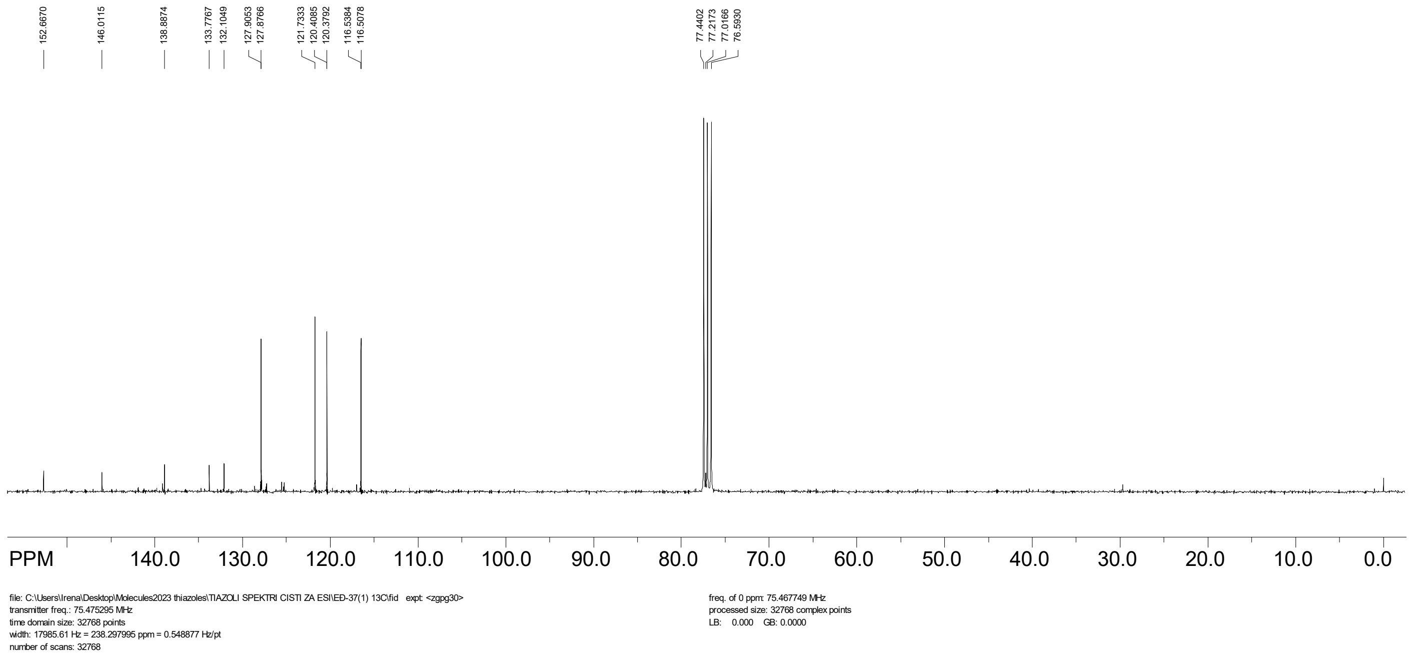

Figure S51. ^{13}C NMR spectrum ($CDCl_3$) of **3**.

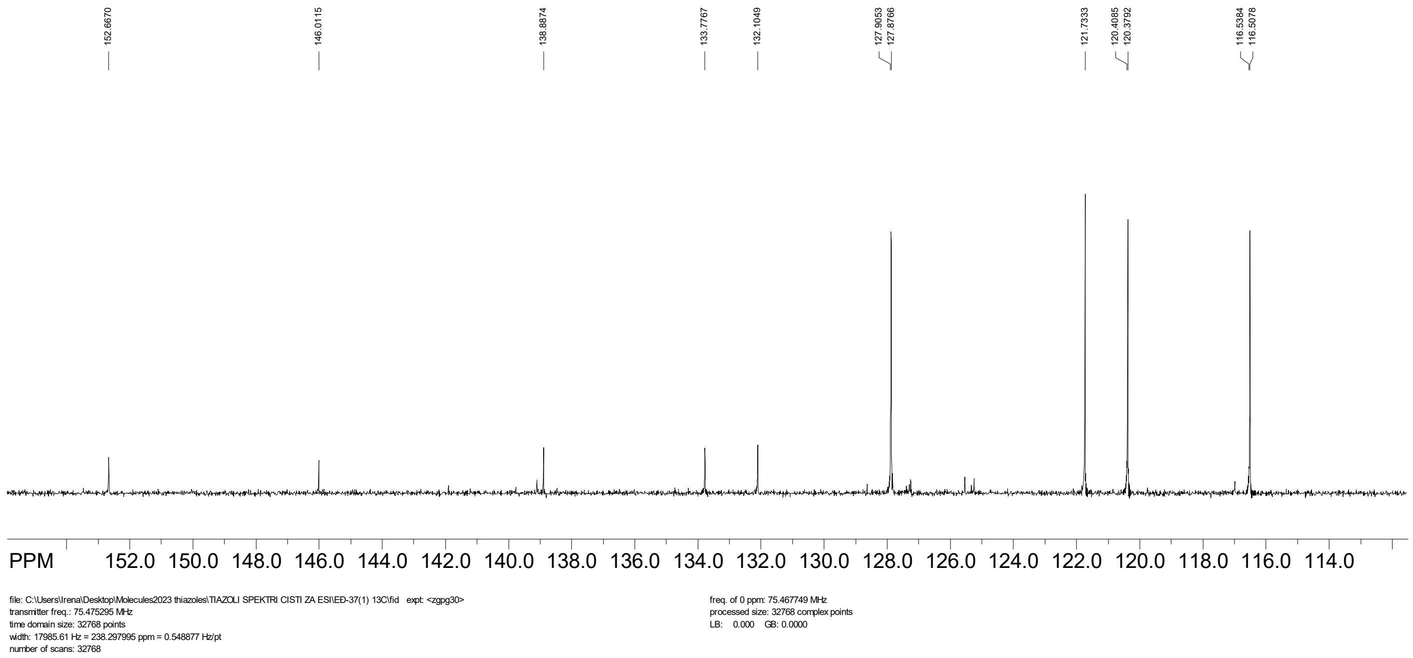

Figure S52. Aromatic part of the ^{13}C NMR spectrum ($CDCl_3$) of **3**.

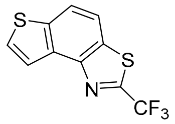

4

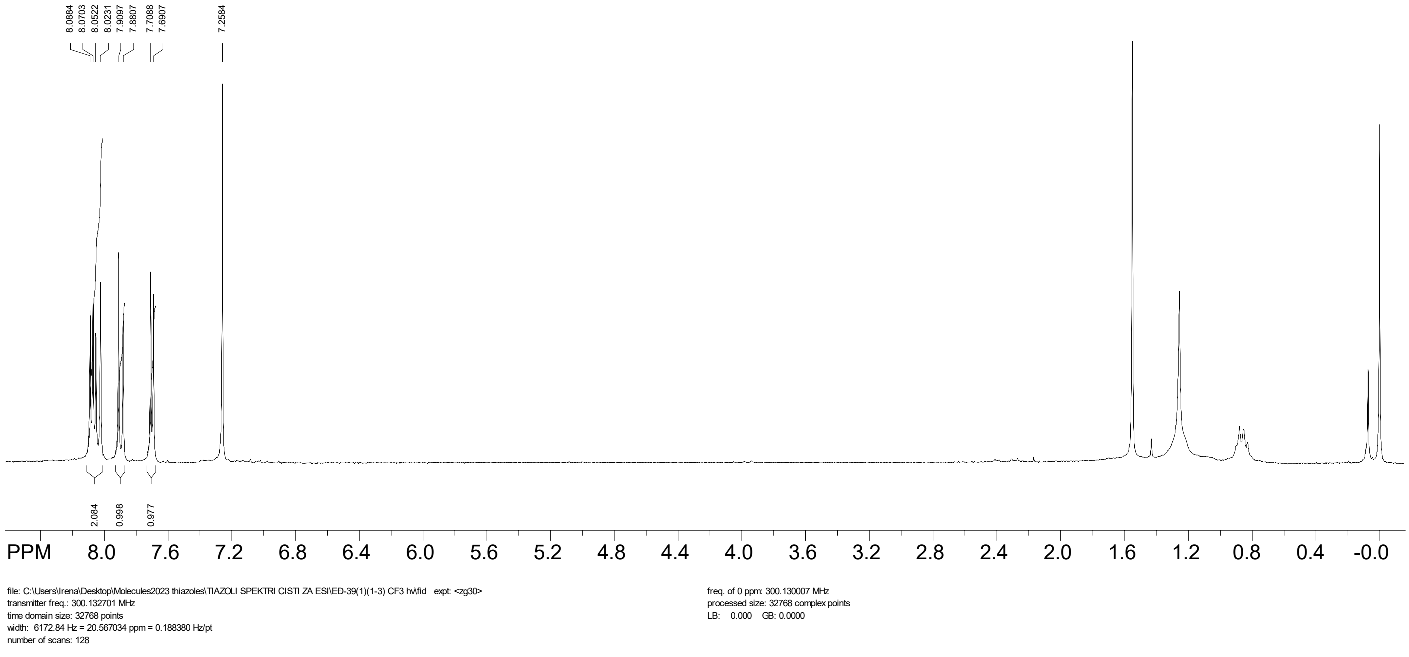

Figure S53. ^{1}H NMR spectrum ($CDCl_3$) of **4**.

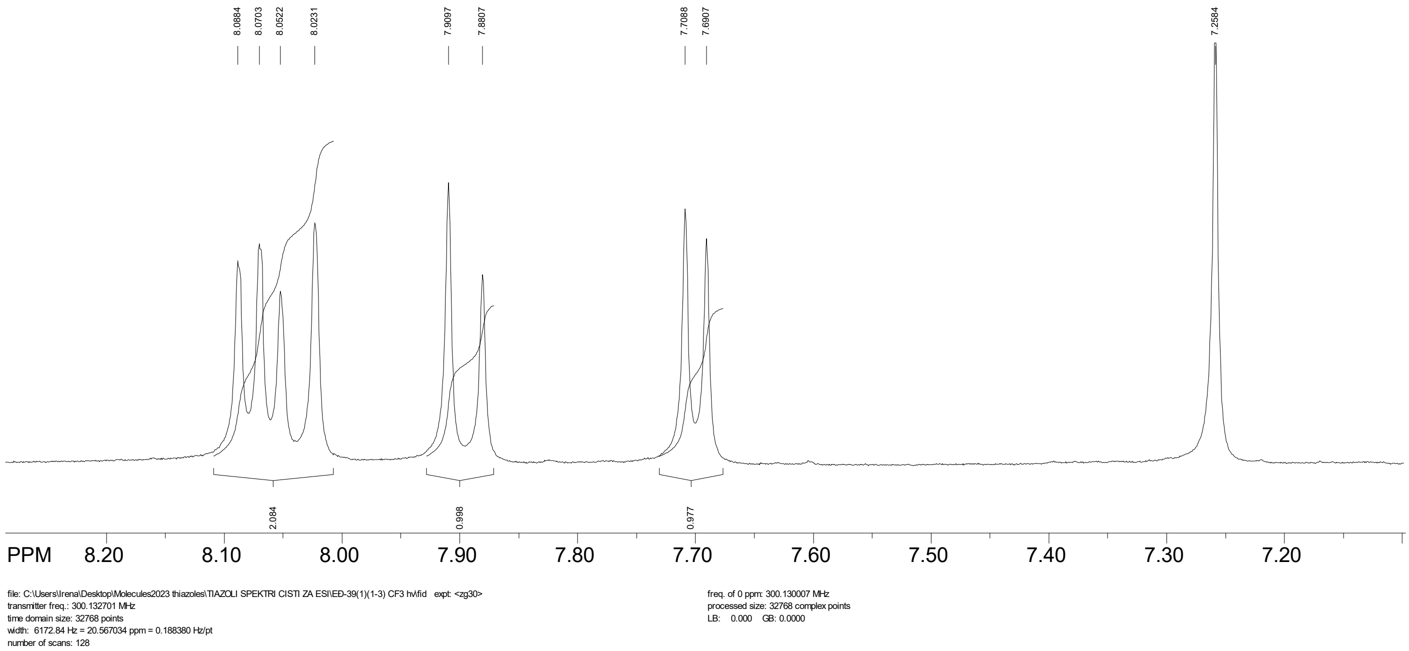

Figure S54. Aromatic part of the ^{1}H NMR spectrum ($CDCl_3$) of **4**.

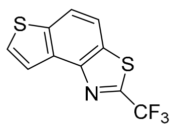

4

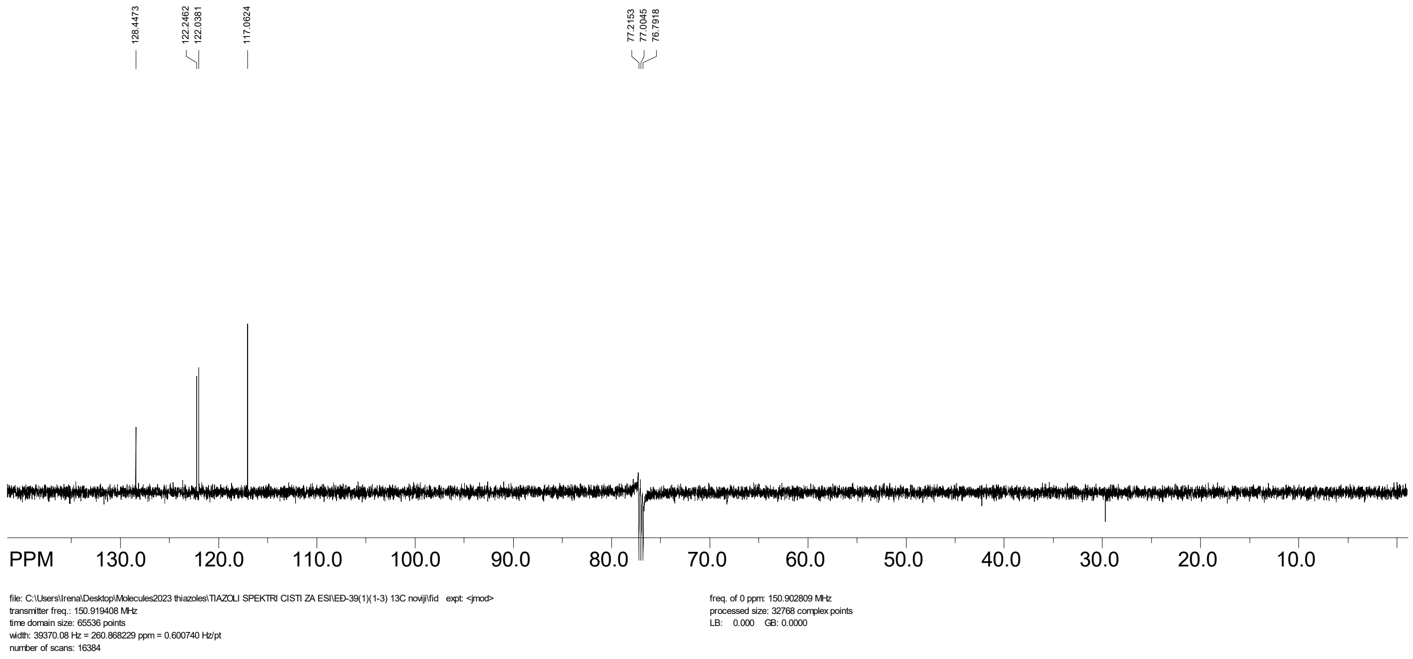

Figure S55. ^{13}C NMR (APT) spectrum ($CDCl_3$) of **4**.

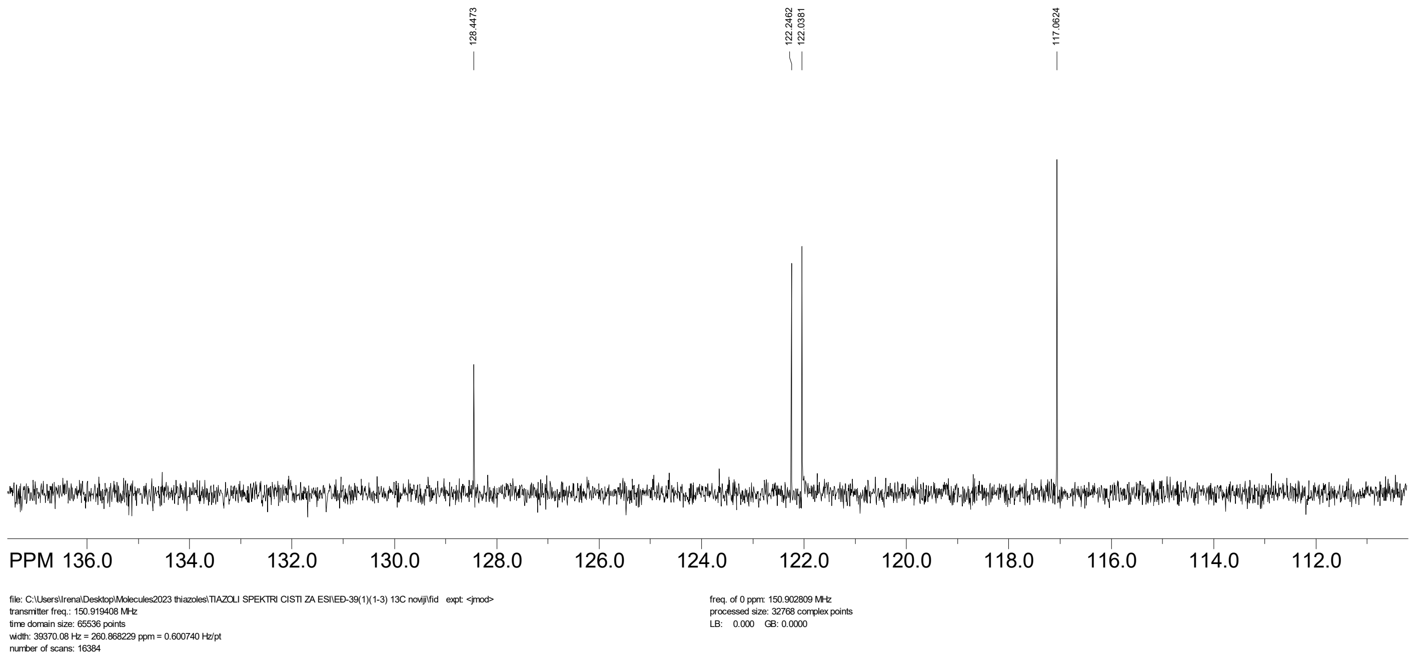

Figure S56. Aromatic part of the ^{13}C NMR (APT) spectrum ($CDCl_3$) of **4**.

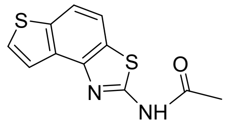

5

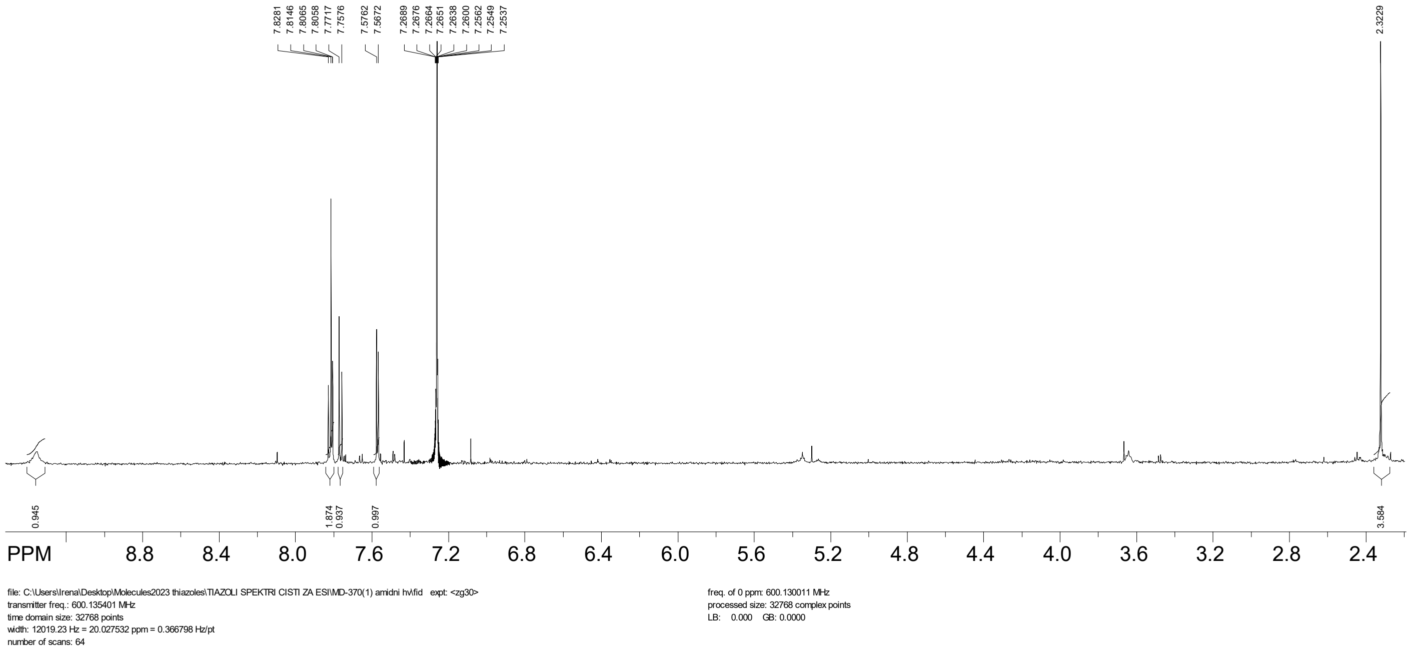

Figure S57. ¹H NMR spectrum ($CDCl_3$) of **5**.

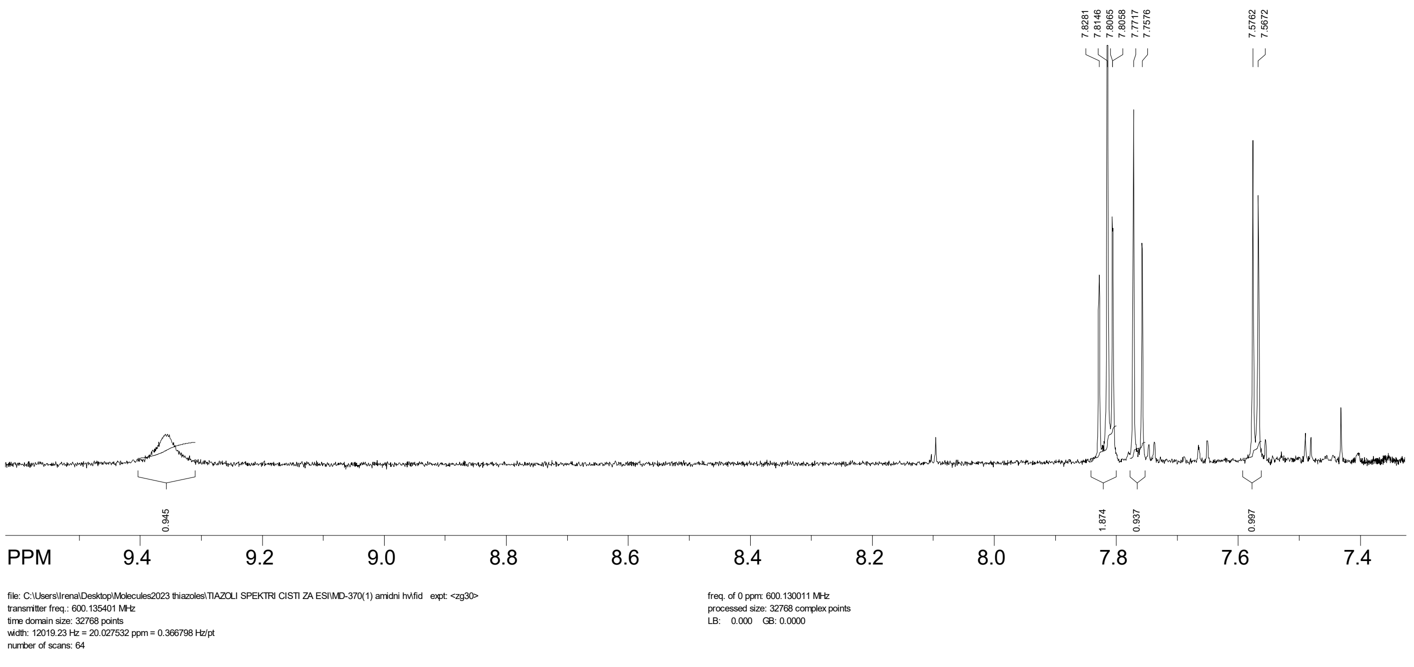

Figure S58. Aromatic part of the ¹H NMR spectrum ($CDCl_3$) of **5**.

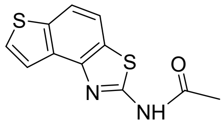

5

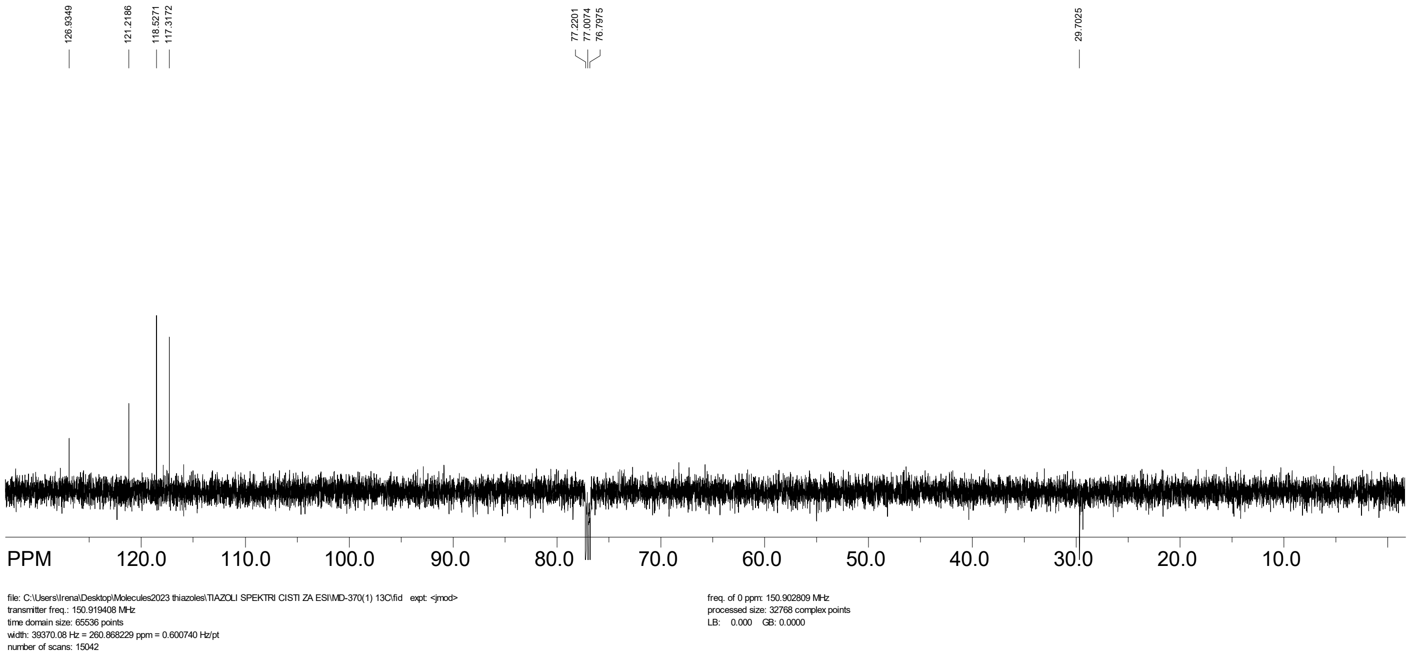

Figure S59. ^{13}C NMR spectrum ($CDCl_3$) of **5**.

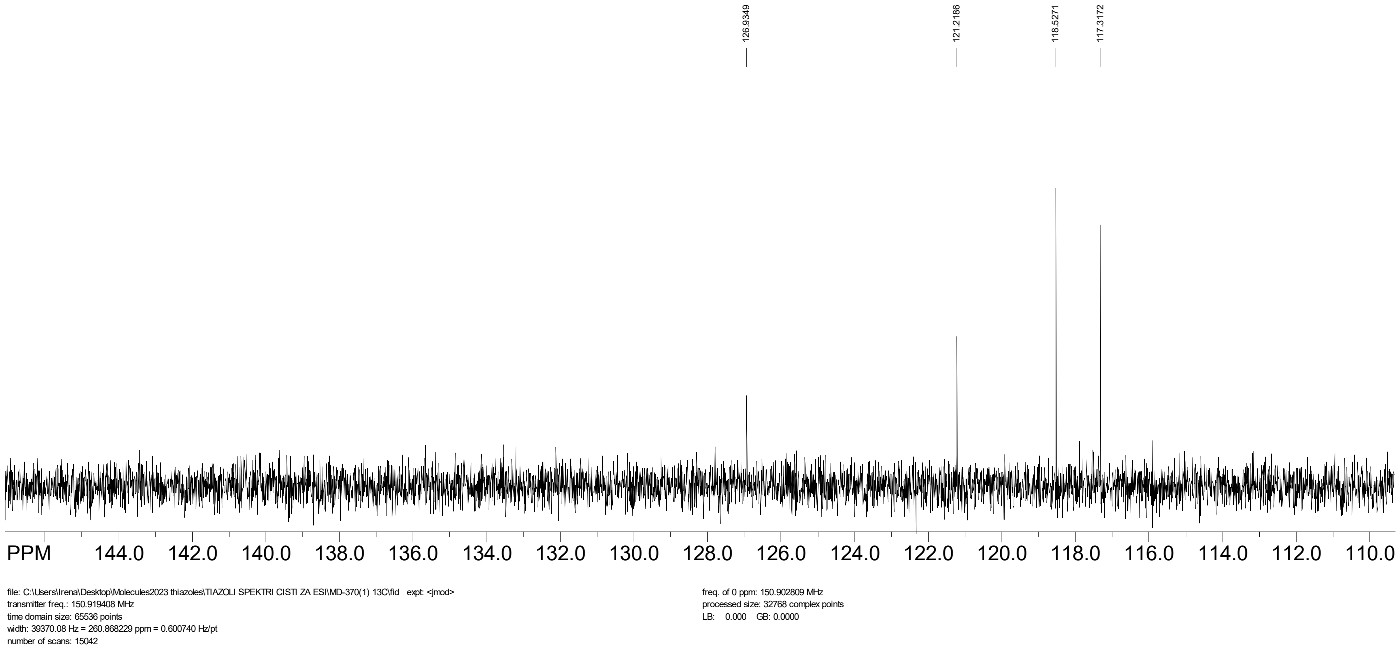

Figure S60. Aromatic part of the ^{13}C NMR spectrum ($CDCl_3$) of **5**.

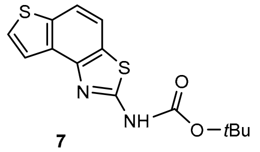

7

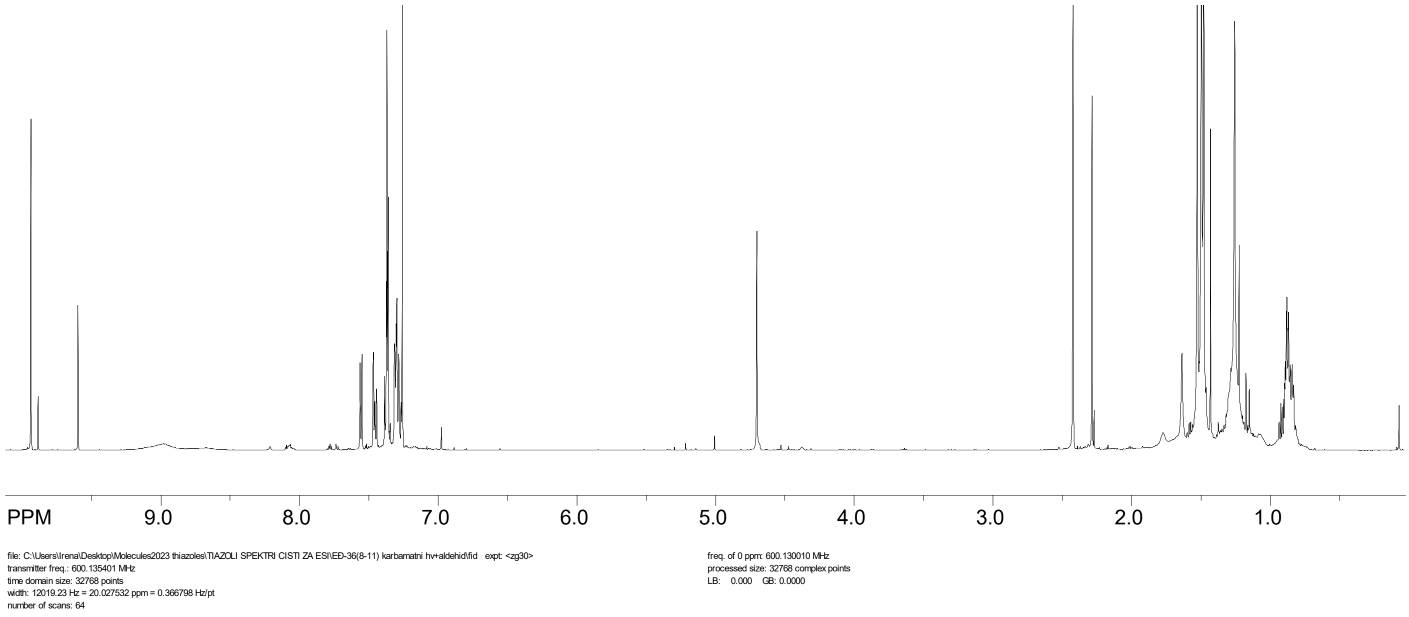

Figure S61. ^{1}H NMR spectrum ($CDCl_3$) of **7** and starting aldehyde from the Wittig reaction.

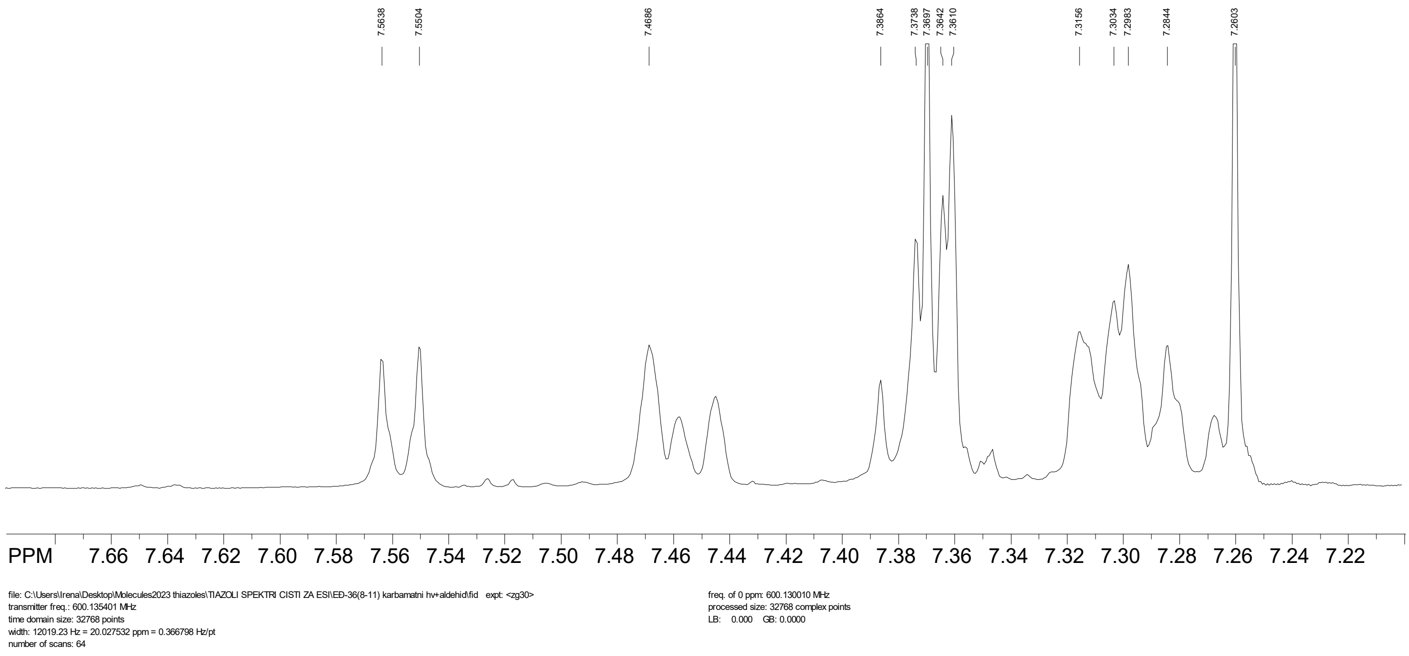

Figure S62. Aromatic part of the ^{1}H NMR spectrum ($CDCl_3$) of **7** and starting aldehyde from the Wittig reaction.

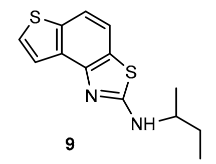

9

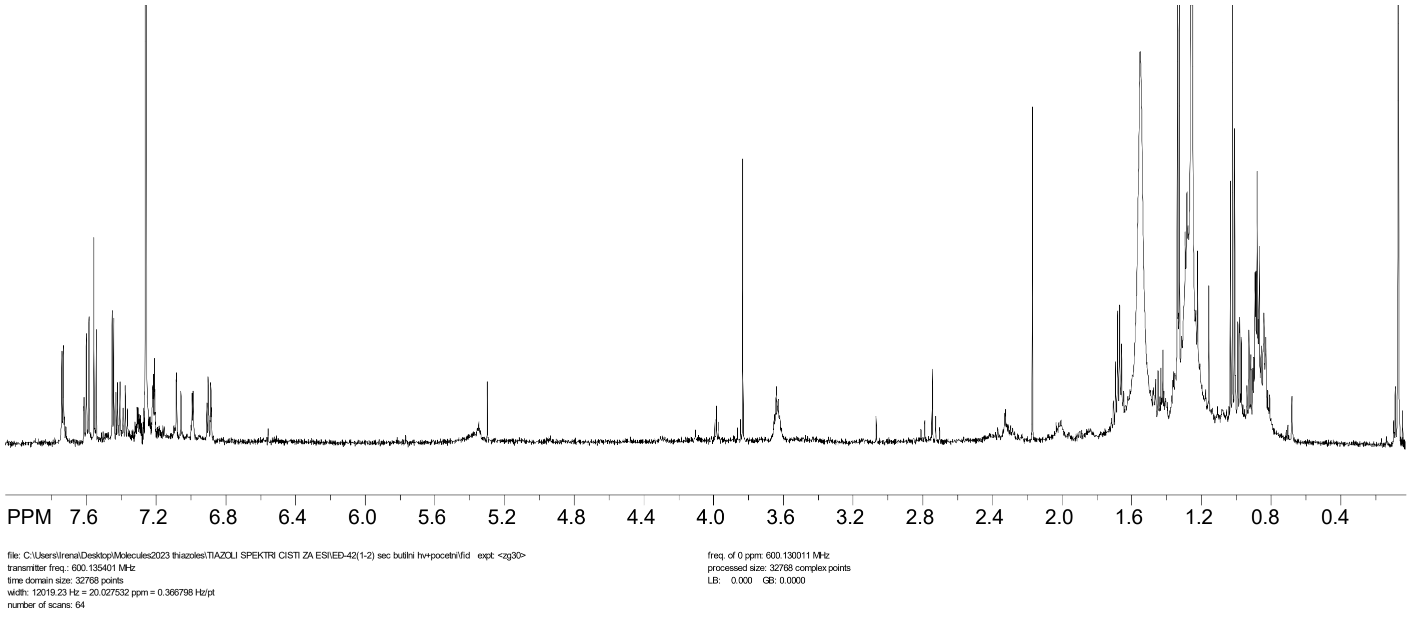

Figure S63. ^{1}H NMR spectrum ($CDCl_3$) of **9** and the starting **9a**.

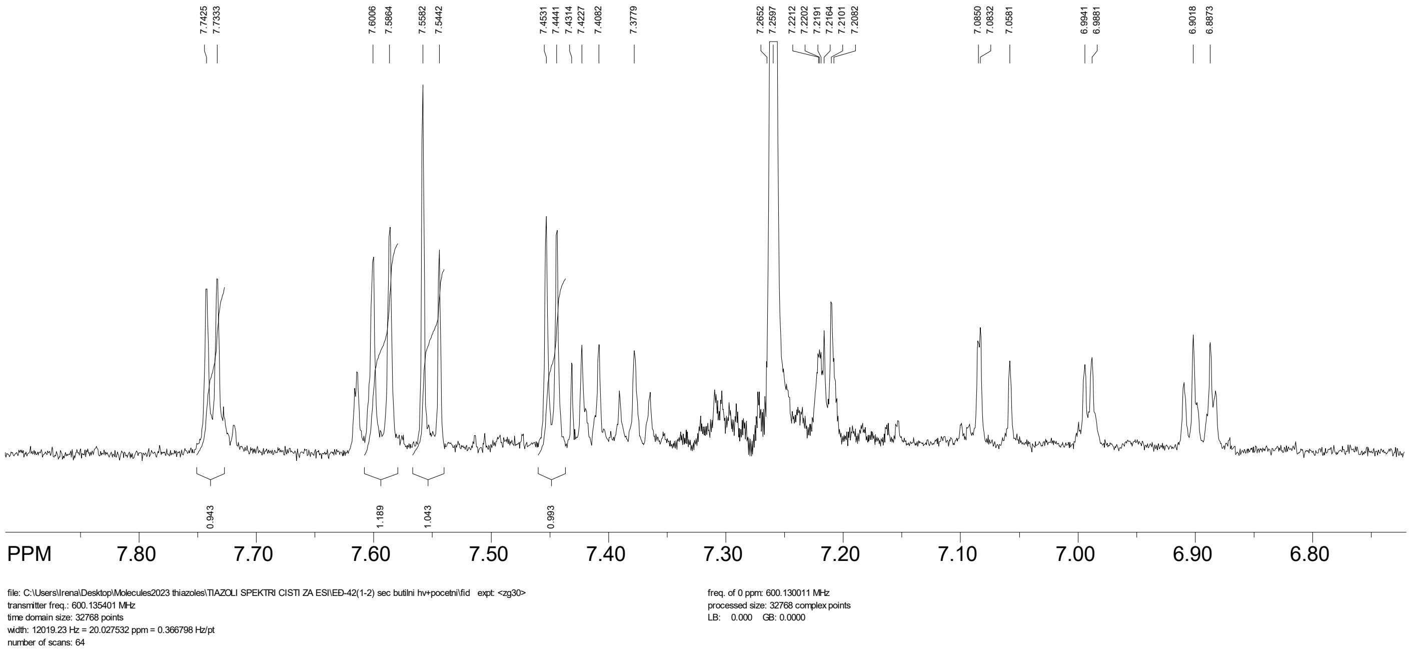

Figure S64. Aromatic part of the ^{1}H NMR spectrum ($CDCl_3$) of **9** as the major compound in the mixture (integrated) and the starting **9a**.

2. MS and HRMS analyses of compounds 1a–10a and 1–5, 7 and 9

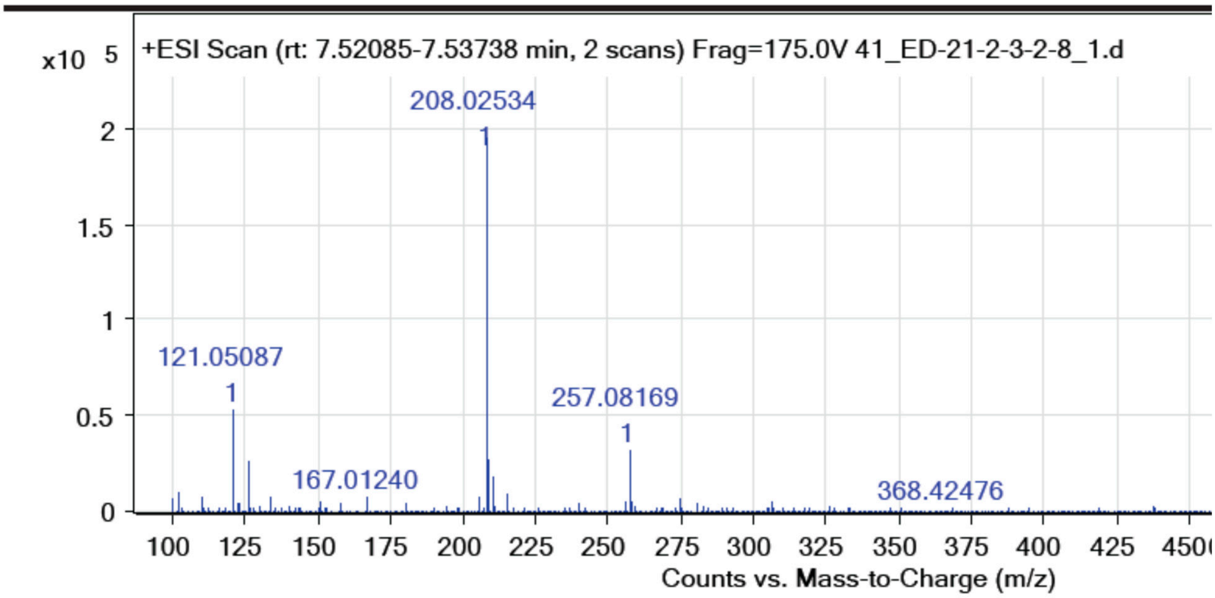

Formula Calculator Results

Formula	Best	Mass	Tgt Mass	Diff (ppm)	Ion Species	Score
C10 H9 N S2	True	207.01803	207.01764	-1.88	C10 H10 N S2	98.07

Figure S65. MS spectrum and HRMS analysis of **1a** as mixture of isomers.

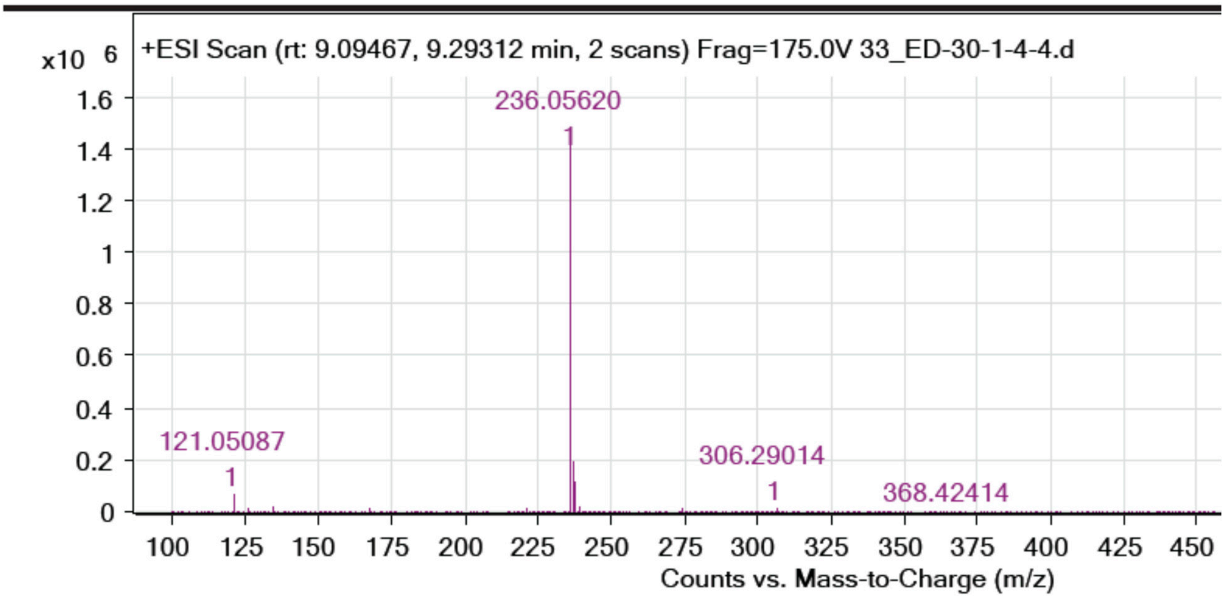

Formula Calculator Results

Formula	Best	Mass	Tgt Mass	Diff (ppm)	Ion Species	Score
C12 H13 N S2	True	235.04893	235.04894	0.04	C12 H14 N S2	98.01

Figure S66. MS spectrum and HRMS analysis of **2a** as mixture of isomers.

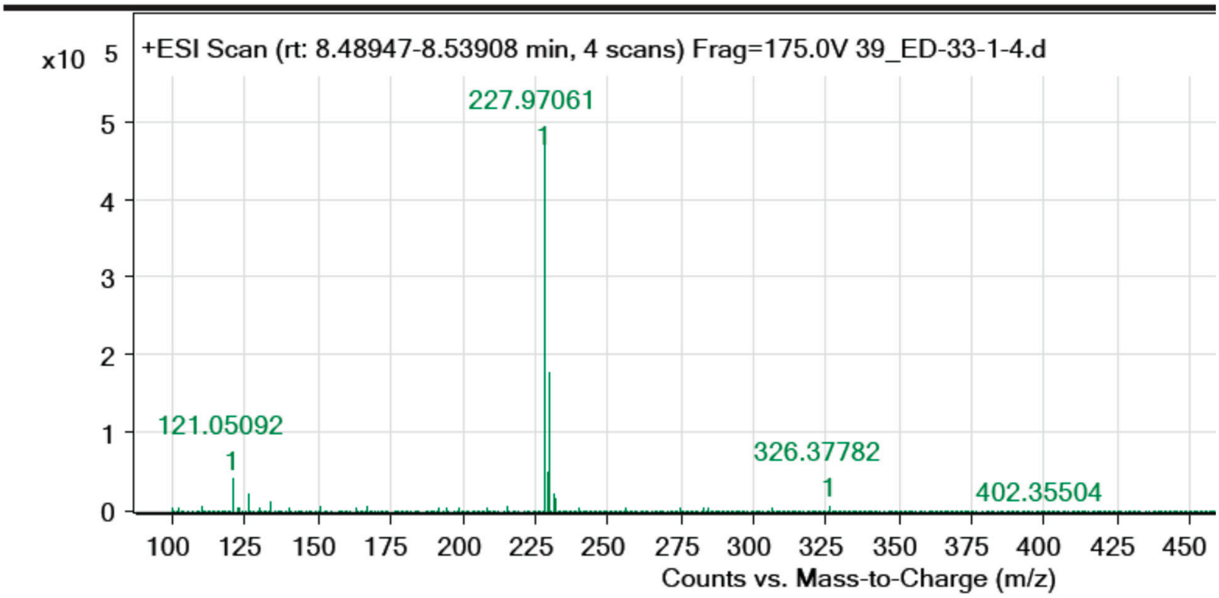

Formula Calculator Results

Formula	Best	Mass	Tgt Mass	Diff (ppm)	Ion Species	Score
C9 H6 Cl N S2	True	226.96332	226.96302	-1.31	C9 H7 Cl N S2	98.1

Figure S67. MS spectrum and HRMS analysis of *trans*-**3a** as mixture of isomers.

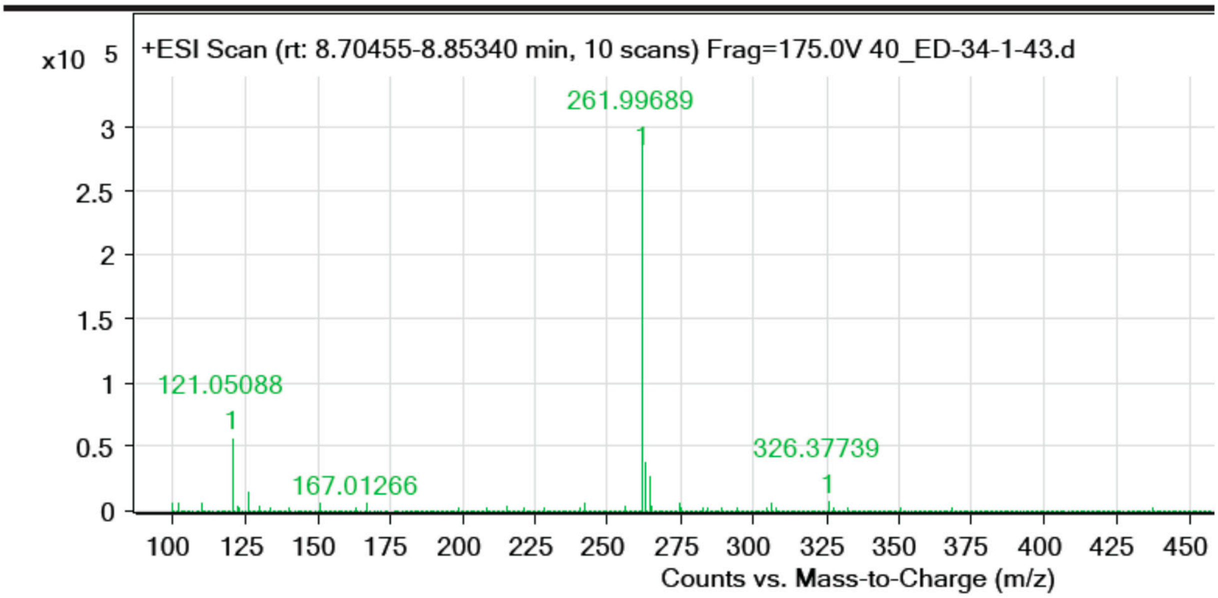

Formula Calculator Results

Formula	Best	Mass	Tgt Mass	Diff (ppm)	Ion Species	Score
C10 H6 F3 N S2	True	260.98957	260.98938	-0.75	C10 H7 F3 N S2	99.19

Figure S68. MS spectrum and HRMS analysis of **4a** as mixture of isomers.

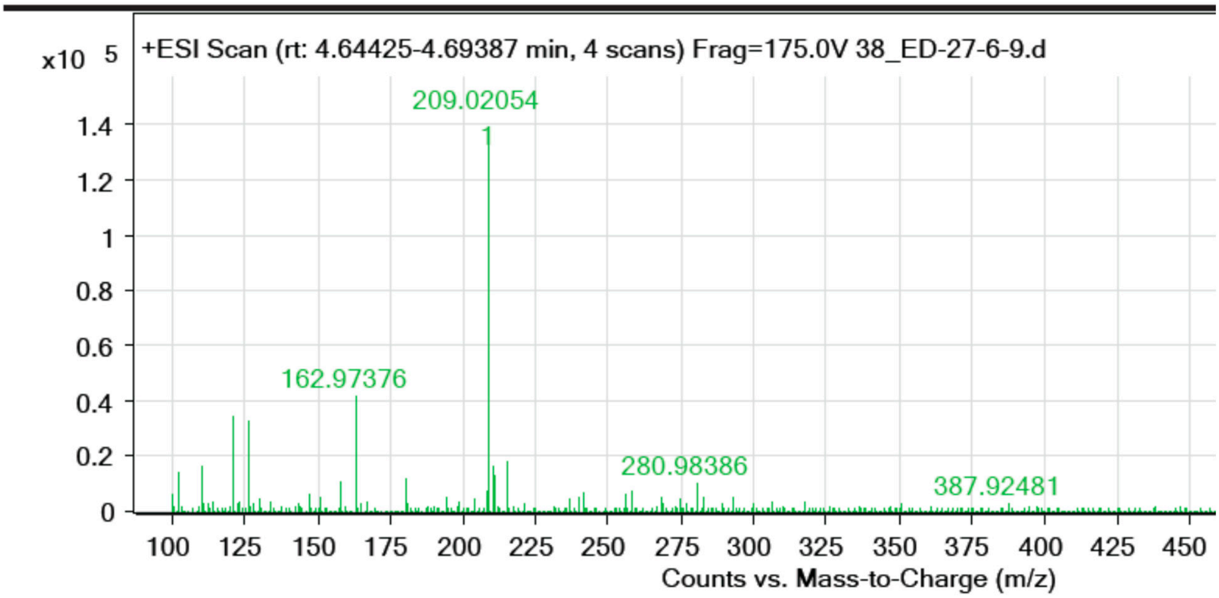

Formula Calculator Results

Formula	Best	Mass	Tgt Mass	Diff (ppm)	Ion Species	Score
C9 H8 N2 S2	True	208.01326	208.01289	-1.79	C9 H9 N2 S2	98.88

Figure S69. MS spectrum and HRMS analysis of **5a'** as mixture of isomers.

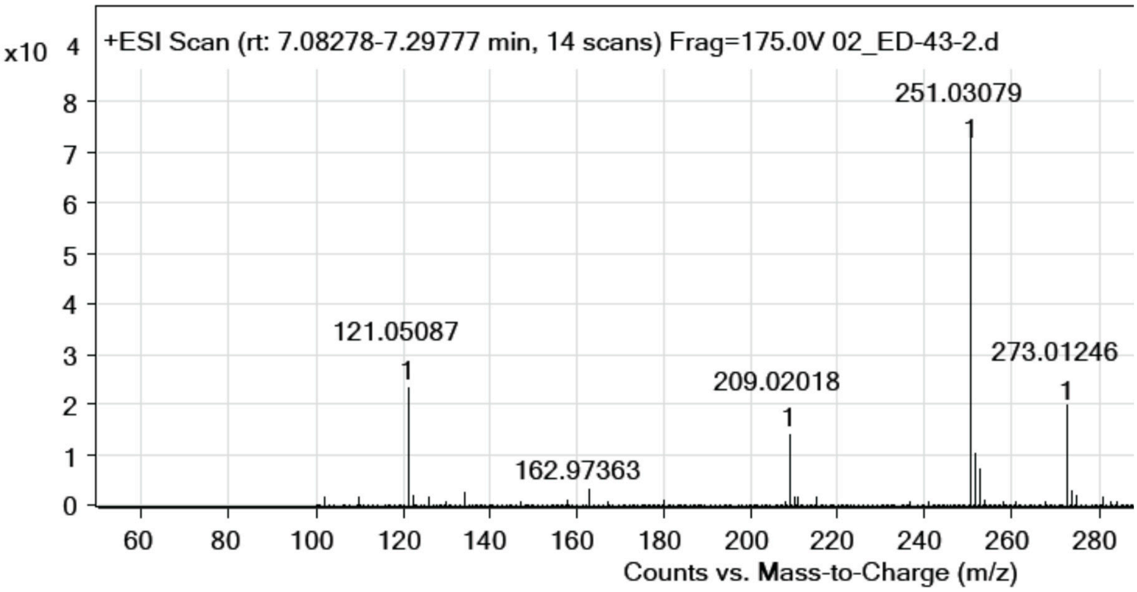

Formula Calculator Results

Formula	Best	Mass	Tgt Mass	Diff (ppm)	Ion Species	Score
C11 H10 N2 O S2	True	250.02351	250.02345	-0.2	C11 H11 N2 O S2	99.24

Figure S70. MS spectrum and HRMS analysis of **5a** as mixture of isomers.

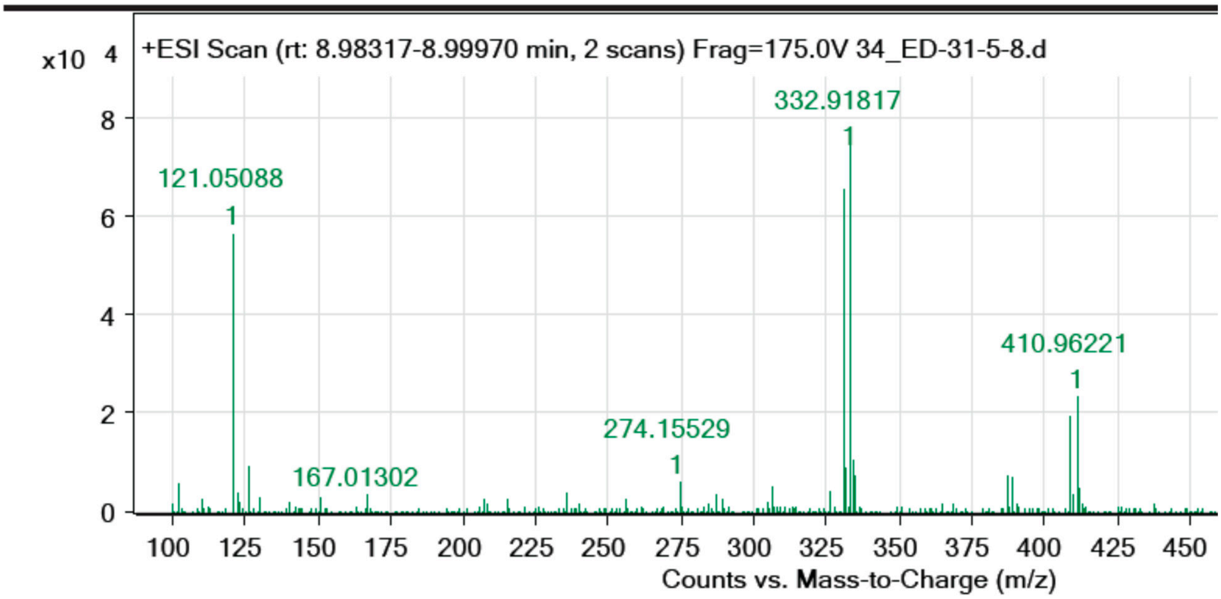

Formula Calculator Results

Formula	Best	Mass	Tgt Mass	Diff (ppm)	Ion Species	Score
C14 H15 Br N2 O2 S2	True	385.9751	385.97583	1.89	C14 H16 Br N2 O2 S2	92.91

Figure S71. MS spectrum and HRMS analysis of **7a** as mixture of isomers.

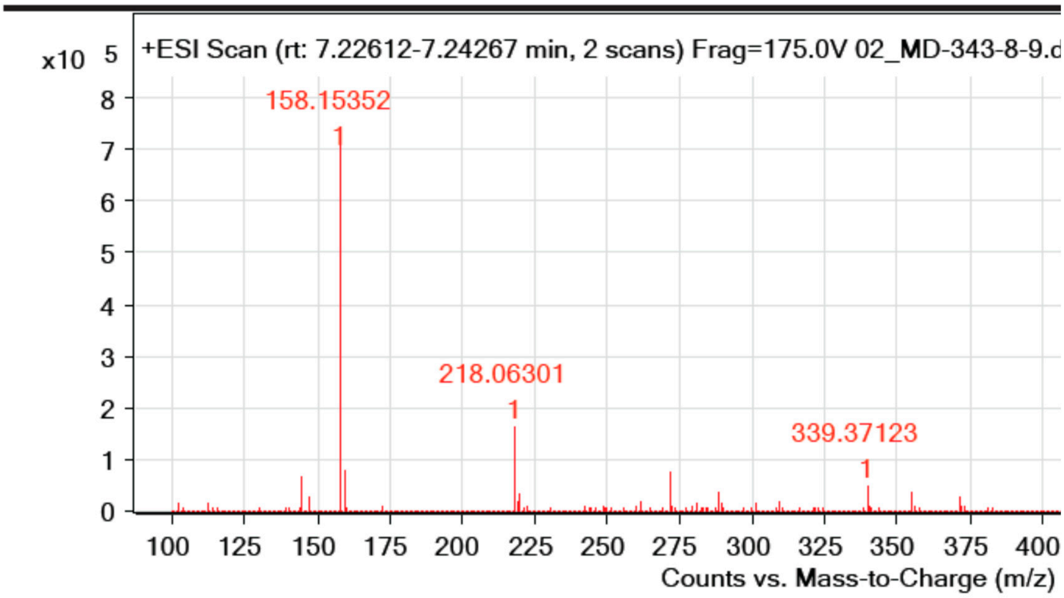

Formula Calculator Results

Formula	Best	Mass	Tgt Mass	Diff (ppm)	Ion Species	Score
C12 H11 N O S	True	217.05574	217.05613	1.8	C12 H12 N O S	63.05

Figure S72. MS spectrum and HRMS analysis of *trans*-**8a**.

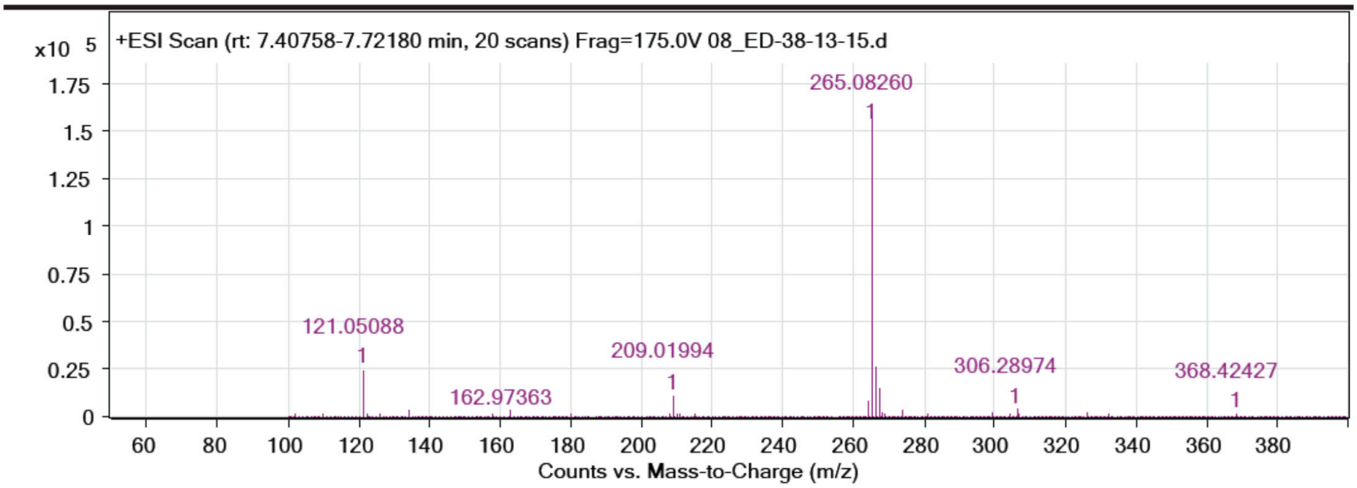

Formula Calculator Results

Formula	Best	Mass	Tgt Mass	Diff (ppm)	Ion Species	Score
C13 H16 N2 S2	True	264.0753	264.07549	0.72	C13 H17 N2 S2	98.71

Figure S73. MS spectrum and HRMS analysis of **9a** as mixture of isomers.

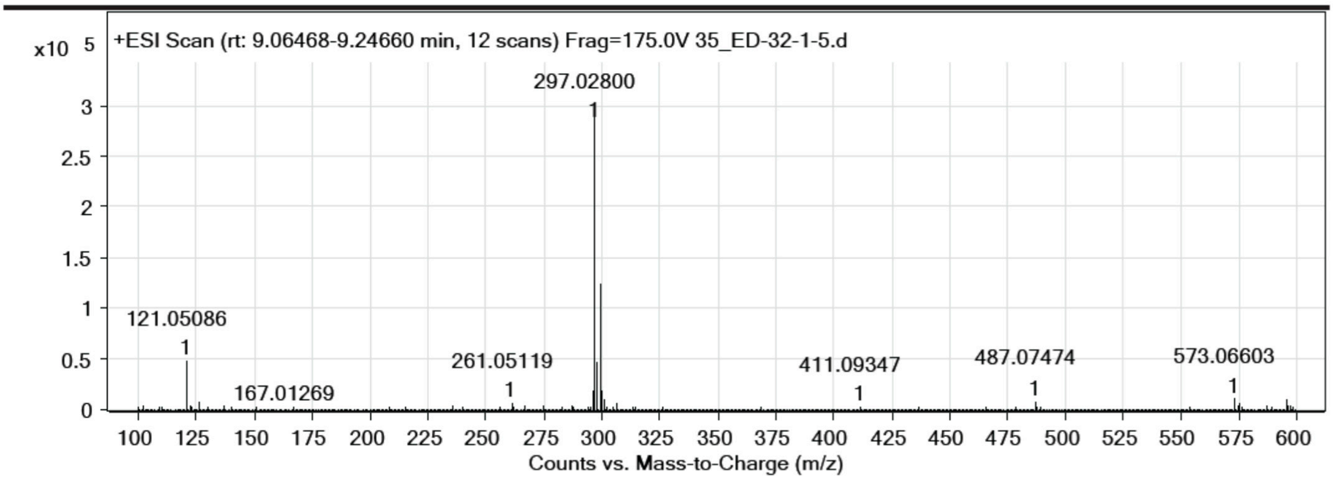

Formula Calculator Results

Formula	Best	Mass	Tgt Mass	Diff (ppm)	Ion Species	Score
C13 H13 Cl N2 S2	True	296.02065	296.02087	0.72	C13 H14 Cl N2 S2	99.56

Figure S74. MS spectrum and HRMS analysis of **10a** as mixture of isomers.

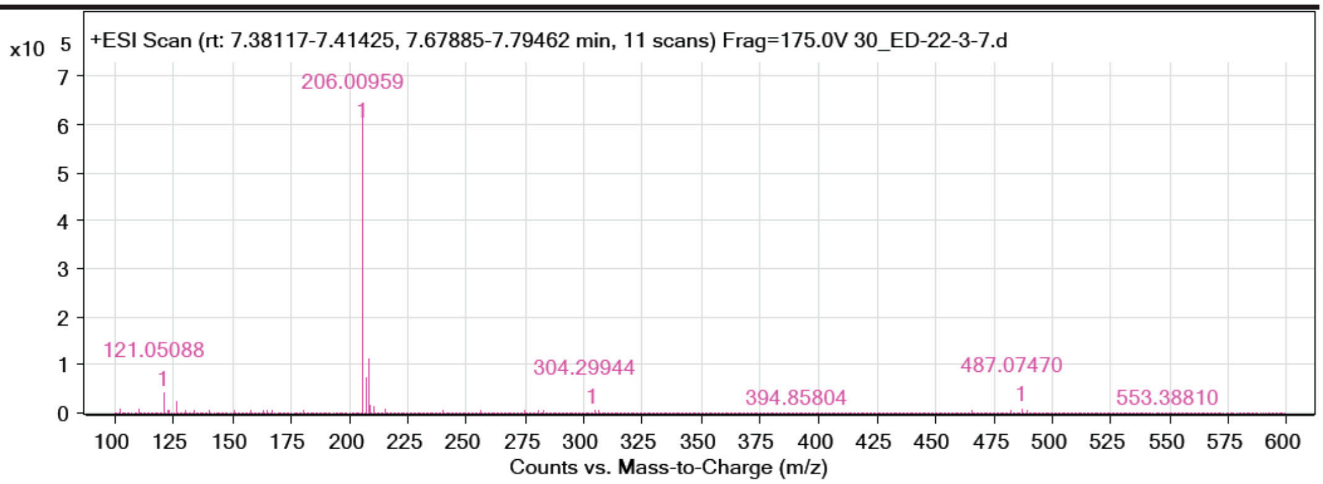

Formula Calculator Results

Formula	Best	Mass	Tgt Mass	Diff (ppm)	Ion Species	Score
C10 H7 N S2	True	205.00229	205.00199	-1.44	C10 H8 N S2	98.11

Figure S75. MS spectrum and HRMS analysis of **1**.

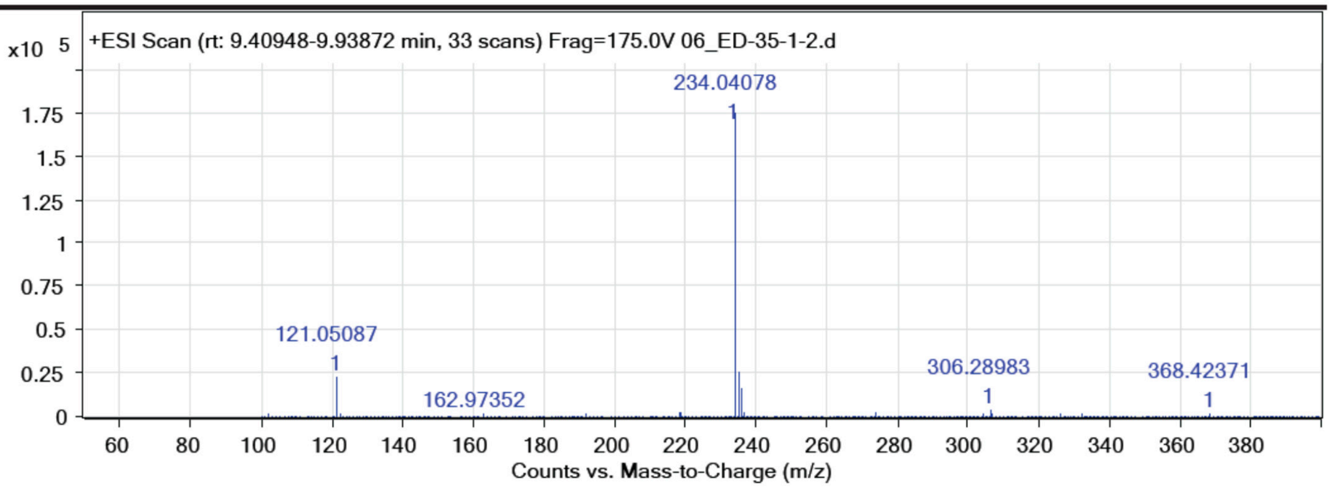

Formula Calculator Results

Formula	Best	Mass	Tgt Mass	Diff (ppm)	Ion Species	Score
C12 H11 N S2	True	233.03349	233.03329	-0.86	C12 H12 N S2	98.96

Figure S76. MS spectrum and HRMS analysis of **2**.

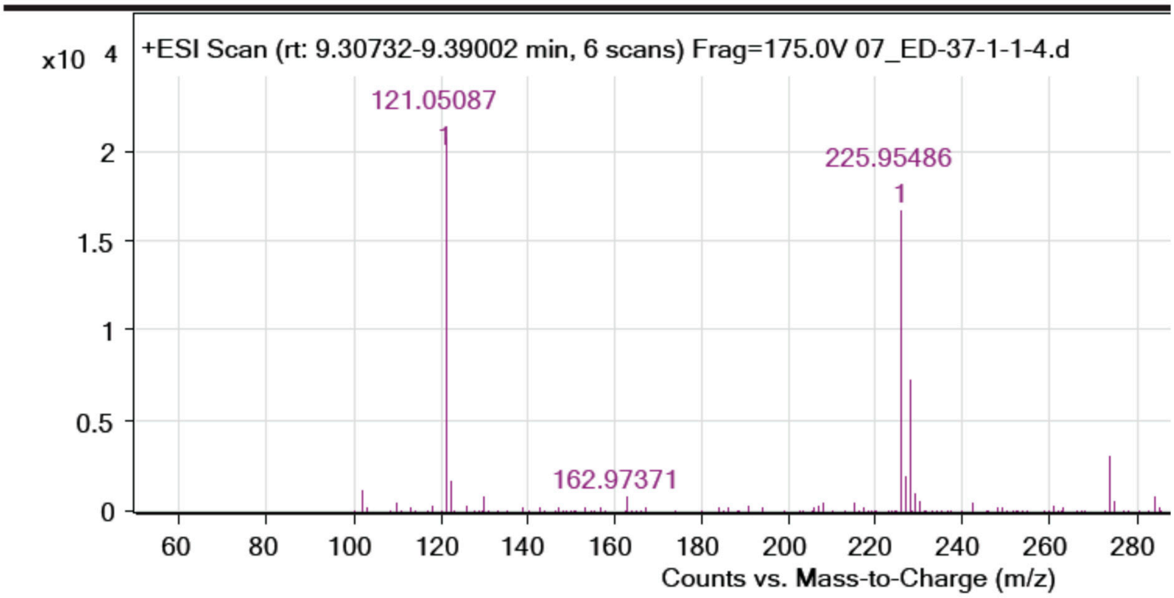

Formula Calculator Results

Formula	Best	Mass	Tgt Mass	Diff (ppm)	Ion Species	Score
C9 H4 Cl N S2	True	224.94752	224.94737	-0.69	C9 H5 Cl N S2	99.32

Figure S77. MS spectrum and HRMS analysis of **3**.

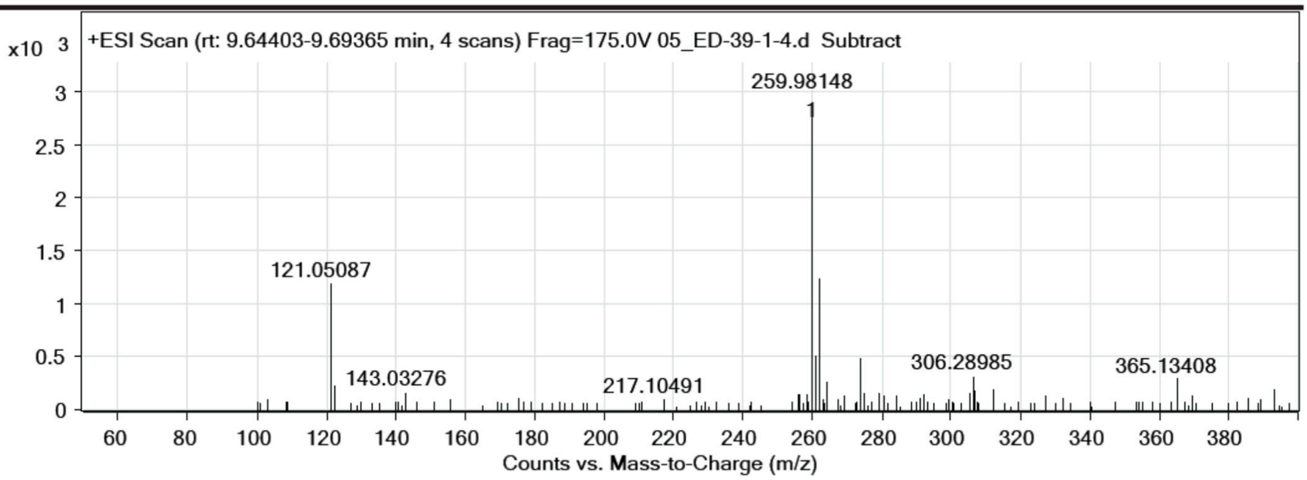

Formula Calculator Results

Formula	Best	Mass	Tgt Mass	Diff (ppm)	Ion Species	Score
C10 H4 F3 N S2	True	258.97404	258.97373	-1.21	C10 H5 F3 N S2	92.02

Figure S78. MS spectrum and HRMS analysis of **4**.

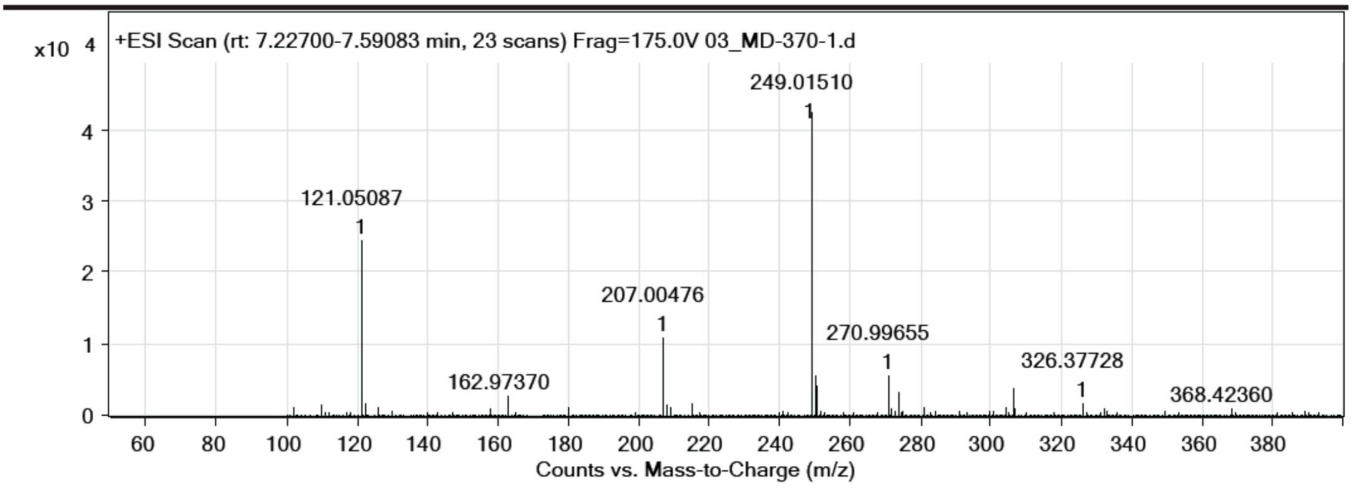

Formula Calculator Results

Formula	Best	Mass	Tgt Mass	Diff (ppm)	Ion Species	Score
C11 H8 N2 O S2	True	248.00789	248.0078	-0.34	C11 H9 N2 O S2	98.58

Figure S79. MS spectrum and HRMS analysis of **5**.

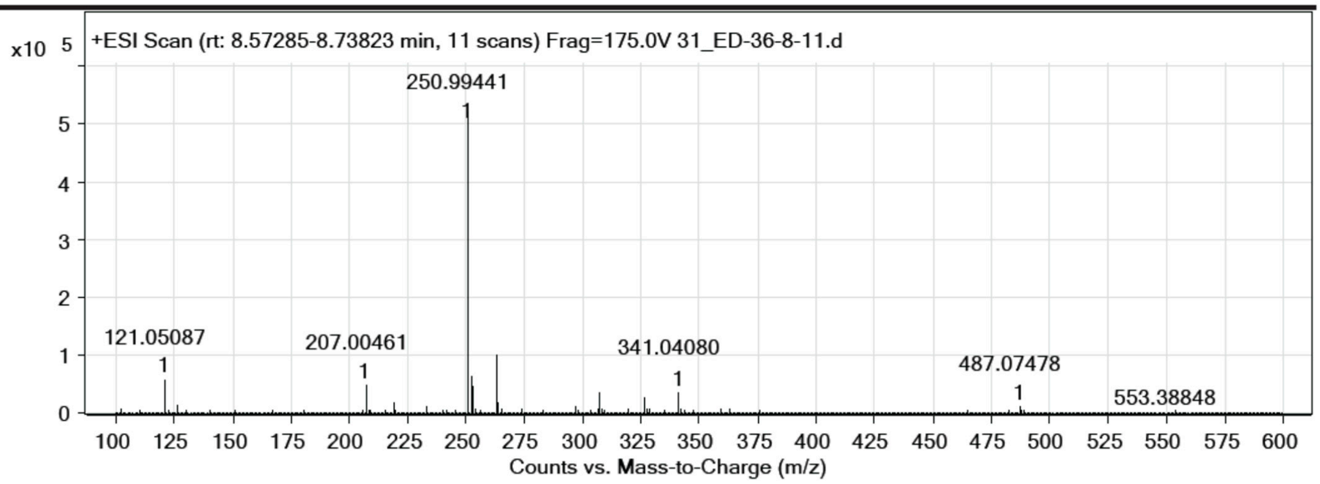

Formula Calculator Results

Formula	Best	Mass	Tgt Mass	Diff (ppm)	Ion Species	Score
C14 H14 N2 O2 S2	True	306.04924	306.04967	1.39	C14 H15 N2 O2 S2	98.08

Figure S80. MS spectrum and HRMS analysis of **7** with starting compound.

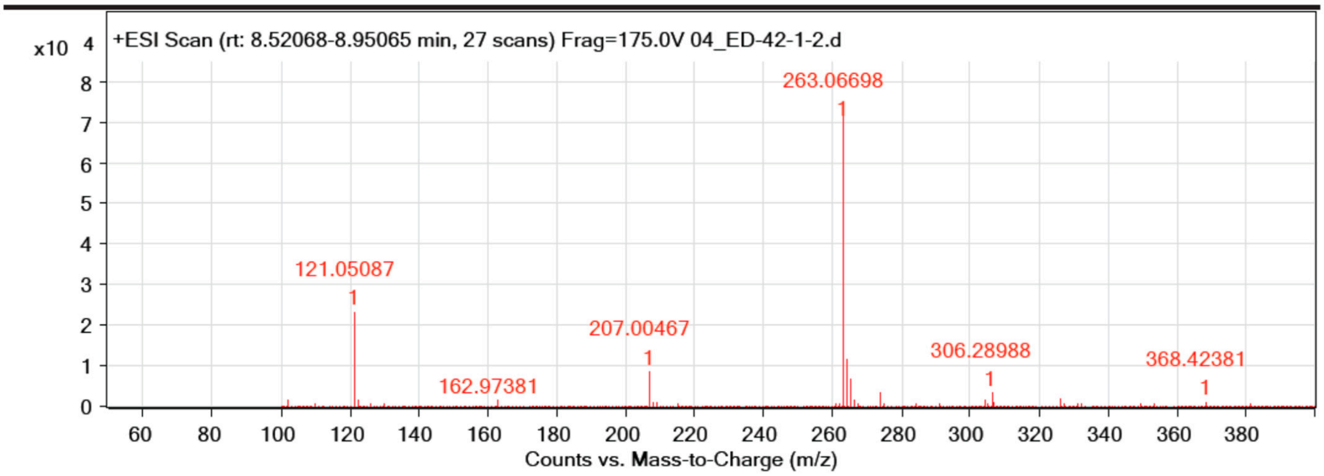

Formula Calculator Results

Formula	Best	Mass	Tgt Mass	Diff (ppm)	Ion Species	Score
C13 H14 N2 S2	True	262.05969	262.05984	0.57	C13 H15 N2 S2	98.99

Figure S81. MS spectrum and HRMS analysis of **9** with starting compound.

Geometries of studied thieno-thiazolostilbenes and thienobenzo-thiazoles

Thieno-thiazolostilbenes
cis-1a

S	1.222767	-1.817924	1.245989
C	0.182542	-0.489927	1.663143
C	0.516490	0.627016	0.944932
C	1.601604	0.413968	0.044966
C	2.083072	-0.859962	0.102003
C	-0.849641	-0.632564	2.695699
C	-2.160421	-0.385739	2.543176
C	-2.923889	-0.055930	1.350441
S	-2.362161	-0.010020	-0.296650
C	-3.994505	0.313928	-0.822689
N	-4.870604	0.390487	0.133954
C	-4.273940	0.178126	1.347321
C	-4.323189	0.494688	-2.271290
H	2.893687	-1.293792	-0.466762
H	2.006842	1.177864	-0.607531
H	-4.881389	0.194830	2.245907
H	-3.768375	1.332876	-2.703127
H	-4.081137	-0.402645	-2.848311
H	-5.392299	0.694378	-2.355909
H	-0.512018	-0.955595	3.678103

H	-2.788052	-0.480328	3.427824
H	0.001222	1.572543	1.071645

cis-2a

C	-0.125403	0.415262	-0.381751
C	-0.041833	0.475372	0.982057
N	1.238180	0.300104	1.484069
C	2.107068	0.100650	0.547147
S	1.423985	0.129879	-1.066198
C	-1.090761	0.782624	1.960482
C	-2.427535	0.771589	1.839708
C	-3.231854	0.310416	0.705278
S	-4.679052	1.168241	0.266552
C	-5.049646	0.035365	-0.974988
C	-4.116333	-0.956827	-1.045485
C	-3.079856	-0.802663	-0.080206
H	-4.167584	-1.775583	-1.753202
H	-5.932185	0.167445	-1.585019
H	-2.996928	1.126392	2.696486
H	-0.680305	1.085101	2.920362
H	-0.999905	0.541399	-1.002866
C	3.573925	-0.167657	0.780203
C	4.450631	0.537815	-0.258771
C	3.963971	0.231371	2.202935
H	3.722674	-1.251323	0.670318
H	5.505471	0.319373	-0.069233
H	4.221571	0.213075	-1.278960
H	4.309303	1.622255	-0.206414
H	5.014517	-0.014776	2.383132
H	3.828461	1.307950	2.345393
H	3.344248	-0.282404	2.939627
H	-2.253966	-1.493492	0.046636

trans-1a

S	-0.052475	-0.094820	0.001294
C	-0.026031	0.021018	1.736939
C	1.278148	0.100527	2.144580
N	2.236364	0.074296	1.164450
C	1.695972	-0.025333	-0.010439
C	-1.203418	0.029428	2.579580
C	-2.475181	-0.050182	2.152979
C	-3.658687	-0.042380	2.990968
C	-4.960144	-0.122603	2.562579
C	-5.907514	-0.090567	3.624868
C	-5.313384	0.013574	4.847100
S	-3.594387	0.074219	4.725119
C	2.467639	-0.079553	-1.290551
H	-2.679379	-0.129193	1.085356
H	-0.994400	0.108599	3.646253
H	-5.785177	0.058711	5.818631

H	-6.980307	-0.142632	3.484575
H	1.582659	0.181513	3.182033
H	2.201972	0.751434	-1.950592
H	3.529643	-0.016751	-1.049303
H	2.278932	-1.012874	-1.829192
H	-5.222373	-0.202055	1.513416

trans-2a

C	-0.482783	0.503264	0.664125
C	-0.370404	0.045169	1.952767
S	1.269040	-0.432081	2.286831
C	1.775594	-0.010975	0.692846
C	0.744686	0.471147	-0.057094
C	-1.434309	-0.041694	2.935652
C	-1.308413	-0.493509	4.192918
C	-2.414792	-0.551893	5.139001
C	-2.321668	-1.001103	6.426083
S	-3.851972	-0.912044	7.207032
C	-4.533103	-0.257006	5.726893
N	-3.680281	-0.133962	4.763771
H	-0.345094	-0.841693	4.562617
H	-2.413459	0.296633	2.602488
H	0.853436	0.792546	-1.086038
H	2.808108	-0.146461	0.402306
H	-1.423292	0.853712	0.253654
C	-5.989231	0.128304	5.644080
H	-1.445223	-1.376410	6.935063
C	-6.343187	0.566776	4.224524
C	-6.893494	-1.017041	6.115044
H	-6.137638	0.980296	6.322051
H	-7.389423	0.883769	4.184923
H	-5.708631	1.391692	3.894837
H	-6.201860	-0.262196	3.524724
H	-7.943011	-0.714307	6.055169
H	-6.752114	-1.897998	5.480555
H	-6.684969	-1.305435	7.150038

trans-3a

C	0.186517	-0.000080	0.349437
C	0.019727	-0.000025	1.713469
S	1.561503	-0.000024	2.512754
C	2.408222	0.000108	1.017211
C	1.551512	0.000216	-0.046103
C	-1.197590	-0.000046	2.507994
C	-2.443708	0.000598	2.004541
C	-3.666208	0.000500	2.780452
S	-3.727890	-0.000664	4.522035
C	-5.464030	0.000313	4.408437
N	-5.961790	0.000979	3.222519

C	-4.947612	0.001107	2.297526
Cl	-6.414984	-0.000175	5.839666
H	-1.064014	-0.000636	3.589519
H	-2.594893	0.001272	0.926703
H	1.882160	0.000351	-1.077675
H	3.488939	0.000169	1.004769
H	-5.198835	0.001757	1.243374
H	-0.640636	0.000040	-0.351015

trans-8a

C	0.027435	0.007836	-0.054621
N	-0.024604	0.156960	1.311936
C	1.142496	-0.024352	1.862396
S	2.418073	-0.399510	0.718558
C	1.259660	-0.293057	-0.553120
C	1.355369	0.078113	3.300136
C	2.546402	-0.103609	3.895489
C	2.898554	-0.031557	5.313263
C	1.993237	0.258196	6.354080
C	2.423108	0.305001	7.678683
C	3.754323	0.066634	8.000116
C	4.669268	-0.221761	6.991926
C	4.233498	-0.266577	5.674653
H	4.943148	-0.491266	4.882615
C	-1.227512	0.181792	-0.853425
H	1.701693	0.531178	8.461052
H	4.070461	0.107449	9.037536
H	5.710673	-0.409671	7.229141
O	0.693389	0.489904	6.029827
H	3.399447	-0.341644	3.257746
H	0.460104	0.316750	3.859149
H	-1.987085	-0.535751	-0.529699
H	-1.637741	1.184760	-0.703828
H	-1.039218	0.035687	-1.919226
H	1.540350	-0.454613	-1.584110
H	0.192600	0.673211	6.837579

trans-9a

S	0.095933	0.142108	0.138247
C	0.014502	0.101492	1.891967
C	1.285426	0.171299	2.382383
N	2.316049	0.227776	1.478254
C	1.848613	0.220170	0.261600
C	-1.248861	0.010169	2.601082
C	-1.376892	-0.526273	3.827876
C	-2.614693	-0.516410	4.593335
C	-3.726079	0.276636	4.447949
C	-4.759549	-0.030555	5.376716
C	-4.420214	-1.043526	6.225549

S	-2.839617	-1.641234	5.899150
N	2.622975	0.296751	-0.854831
C	4.079911	0.170207	-0.752857
H	4.302825	-0.606579	-0.007862
C	4.687535	1.488585	-0.280635
H	-2.130698	0.408723	2.102031
H	-0.514171	-1.005777	4.286789
H	-5.006902	-1.468705	7.027537
H	-5.708823	0.489906	5.419869
H	-3.787026	1.073446	3.715579
H	1.516102	0.211603	3.441214
H	2.198058	0.009964	-1.725638
C	4.608629	-0.275736	-2.116550
H	5.756650	1.374097	-0.080146
H	4.551523	2.262505	-1.043866
H	4.200366	1.809296	0.642281
C	6.121590	-0.473897	-2.142496
H	4.113312	-1.215691	-2.392449
H	4.319113	0.473263	-2.866672
H	6.439502	-0.899631	-3.097710
H	6.652379	0.472130	-2.006967
H	6.439428	-1.156599	-1.347421

trans-10a

C	0.131685	-0.020841	0.372963
C	-0.011760	0.005931	1.737963
S	1.546105	0.043523	2.511519
C	2.363096	0.022763	0.992486
C	1.490305	-0.011165	-0.053551
C	-1.260796	0.004183	2.474104
C	-1.387794	0.030493	3.813626
C	-2.633581	0.028521	4.537076
C	-2.845167	0.051822	5.888286
N	-4.118947	0.038679	6.364270
C	-4.952182	0.004758	5.354737
S	-4.209334	-0.010243	3.759252
N	-6.288096	-0.011742	5.491075
C	-6.926859	0.066741	6.804444
C	-8.379868	0.390257	6.446060
C	-8.569508	-0.292540	5.085427
C	-7.230644	-0.046957	4.381432
Cl	-1.553991	0.094433	7.040348
H	-0.500124	0.056078	4.444134
H	-2.151569	-0.021507	1.846467
H	1.801304	-0.028671	-1.091094
H	3.443549	0.036972	0.962982
H	-7.224102	0.908315	3.836650
H	-6.970242	-0.843709	3.674381
H	-8.719035	-1.368170	5.223518
H	-9.414767	0.100538	4.517242
H	-9.083802	0.038852	7.203070

H	-8.502661	1.473207	6.341040	
H	-6.830771	-0.892292	7.328760	
H	-6.438779	0.832217	7.412990	
H	-0.718361	-0.046662	-0.299841	

Thienobenzo-thiazoles

1

C	-0.921114	-0.523620	0.000060	-0.336276
C	0.468697	-0.280019	0.000039	-0.369874
C	0.928175	1.042913	0.000032	1.426548
C	0.052474	2.143012	0.000022	-0.513438
C	-1.308507	1.910764	0.000022	0.231021
C	-1.781710	0.585696	0.000037	-0.972895
S	2.669611	1.030483	-0.000024	0.096595
C	2.623486	-0.733374	-0.000039	0.339665
N	1.445425	-1.258957	-0.000032	-0.146248
H	-2.005103	2.742700	-0.000013	0.246323
H	0.437256	3.157035	0.000003	0.249316
C	-1.627619	-1.773490	0.000059	-0.642906
C	-2.970625	-1.598065	0.000035	-0.198123
S	-3.448791	0.073802	-0.000056	-0.107136
C	3.894639	-1.521068	0.000005	-0.618932
H	3.642535	-2.582212	-0.000520	0.261102
H	4.497110	-1.294649	-0.884542	0.264163
H	4.496541	-1.295417	0.885142	0.264191
H	-1.132239	-2.736139	-0.000191	0.253347
H	-3.734580	-2.363672	-0.000013	0.273469

2

S	-4.093695	0.189810	0.107581	-0.131384
C	-2.416407	0.663723	0.068854	-1.141945
C	-1.582136	-0.462584	-0.049456	0.974046
C	-2.325704	-1.692001	-0.106404	-0.479546
C	-3.662220	-1.486973	-0.033279	-0.121724
C	-1.916343	1.977728	0.142498	0.361327
C	-0.554466	2.180547	0.097225	-1.428231
C	0.313656	1.075397	-0.021529	0.221502
C	-0.196477	-0.226460	-0.093811	0.590467
N	1.696029	1.170349	-0.076898	-0.139108
C	2.244098	0.011106	-0.184469	-0.151141
S	1.114096	-1.357355	-0.227930	0.028781
C	3.727888	-0.237401	-0.276113	0.270567
H	-2.597749	2.817137	0.233248	0.246570
H	-0.127901	3.176050	0.150483	0.241893
H	3.915145	-0.721529	-1.244671	0.257380
C	4.493472	1.083283	-0.228830	-0.822035
C	4.194154	-1.192017	0.830933	-0.740954
H	-1.868558	-2.670438	-0.197771	0.253502

H	-4.443892	-2.234056	-0.053773	0.274863
H	5.270590	-1.366522	0.745014	0.240246
H	3.993188	-0.757136	1.815328	0.244320
H	3.688502	-2.160816	0.777080	0.235625
H	5.564774	0.894161	-0.342832	0.230772
H	4.164630	1.759926	-1.020067	0.239336
H	4.325570	1.585403	0.728125	0.244754

3

S	3.739325	-0.143251	0.000209	-0.094631
C	2.124452	0.511830	-0.000125	-0.904300
C	1.170631	-0.518669	0.000083	-0.488524
C	1.763592	-1.825394	0.000077	-0.696267
C	3.116986	-1.766183	-0.000449	-0.174687
C	1.769469	1.873346	-0.000299	0.215354
C	0.435100	2.227221	-0.000271	-0.435395
C	-0.533181	1.207832	0.000014	1.421935
C	-0.190940	-0.150755	0.000192	-0.425108
S	-2.271337	1.355328	0.000127	0.111673
C	-2.353496	-0.395339	0.000564	0.164370
N	-1.249844	-1.043161	0.000322	-0.056381
Cl	-3.895988	-1.155825	-0.000293	0.326178
H	2.537015	2.640182	-0.000372	0.250097
H	0.141946	3.271232	-0.000370	0.254032
H	1.186775	-2.741535	0.000135	0.255574
H	3.811498	-2.595304	-0.000765	0.275983

4

S	-4.245845	-0.350669	-0.000004	-0.090979
C	-2.692800	0.438154	0.000056	-0.508394
C	-1.655658	-0.506920	-0.000037	0.183445
C	-2.134662	-1.858646	-0.000079	-0.956282
C	-3.488860	-1.914111	0.000030	-0.060057
C	-2.454062	1.826431	0.000127	-0.099059
C	-1.156918	2.295365	0.000097	0.209870
C	-0.103282	1.361919	0.000005	0.785667
C	-0.329386	-0.021599	-0.000060	-0.312683
S	1.610157	1.663189	-0.000148	0.265298
C	1.851997	-0.067472	-0.000112	-1.057053
N	0.803057	-0.811469	-0.000121	-0.054383
C	3.251935	-0.626235	0.000041	1.502565
H	-3.285116	2.524147	0.000176	0.252339
H	-0.955066	3.360671	0.000123	0.256421
F	3.241472	-1.951523	-0.000941	-0.307427
F	3.927087	-0.203666	-1.076592	-0.271431
F	3.926243	-0.205271	1.077827	-0.271318
H	-1.482633	-2.722866	-0.000133	0.256399
H	-4.110599	-2.799142	0.000057	0.276846

5

```
S    4.404961   -0.248277   -0.000147 -0.113053
C    2.805701    0.448171   -0.000059 -0.799261
C    1.826299   -0.558200    0.000093 -0.399445
C    2.388944   -1.879352    0.000134 -0.703183
C    3.743018   -1.856500   -0.000078 -0.196462
C    2.485986    1.818031   -0.000127  0.402257
C    1.158424    2.199140   -0.000056 -0.744600
C    0.165356    1.205066    0.000115  1.235545
C    0.473026   -0.159572    0.000155 -0.007743
S   -1.570949    1.414763    0.000056  0.383315
C   -1.706599   -0.335775    0.000156  0.186986
N   -0.608080   -1.018898    0.000194 -0.252824
N   -2.929021   -0.983470    0.000299 -0.455509
C   -4.167408   -0.372953    0.000138  0.752849
H    3.271364    2.566462   -0.000246  0.245176
H    0.888833    3.250021   -0.000040  0.247965
H    1.789473   -2.780949    0.000255  0.251832
H    4.416485   -2.702781   -0.000106  0.273391
H   -2.855998   -1.993745    0.000506  0.492555
C   -5.333916   -1.334111   -0.000460 -1.083554
O   -4.282750    0.834445    0.000008 -0.526112
H   -6.256464   -0.755913    0.005539  0.266185
H   -5.307499   -1.969993   -0.890412  0.272178
H   -5.301659   -1.979541    0.882373  0.271735
```

Table S1. Results obtained by molecular docking.

Molecule	DG_{bind}/kcal mol^{-1}	Number of distinctive conformational clusters	Distribution of conformations within clusters
**4**	−4.63	1	25
**15**	−5.14	1	25
trans-**8**	−4.65	5	11 + 2 + 3 + 8 + 1

```
Optimized geometry of complex of compound 4 and the active site of BChE
scf done: -5807.637725
 C   -23.474002   -17.457004    35.214004
 C   -19.479004   -10.103009    34.396997
 C   -10.028029    -9.157991    38.895017
 C   -14.647972   -13.805000    40.847934
 C   -14.757009   -15.940995    43.993045
 C   -11.676990    -3.152990    33.612995
 C   -11.608993    -6.215980    43.983025
 C   -13.279928    -9.172078    45.734874
 C   -11.233025   -12.314952    45.157053
 C   -20.581771   -17.597280    48.058060
```

C	-15.416002	-20.836019	46.740998
C	-22.439003	-5.657016	49.617966
C	-25.971455	-12.807736	46.656949
C	-28.989823	-16.761971	44.199033
C	-20.920995	-5.424979	45.684050
H	-22.560511	-16.887701	34.999640
H	-23.860282	-17.850255	34.267111
H	-23.204538	-18.318544	35.833842
C	-24.518856	-16.588996	35.913760
H	-24.780911	-15.720201	35.294464
H	-24.107913	-16.167797	36.842323
C	-25.831293	-17.320581	36.278687
O	-26.727323	-16.558250	36.792677
O	-25.929605	-18.543850	36.063950
H	-18.715493	-10.889644	34.379827
H	-18.972392	-9.139455	34.272740
H	-20.136784	-10.247259	33.532046
C	-20.281091	-10.147034	35.708937
H	-20.755399	-11.131747	35.806974
H	-19.585229	-10.059538	36.554101
C	-21.337151	-9.080731	35.810554
C	-22.696577	-9.260319	35.717839
H	-23.261110	-10.172899	35.579497
C	-21.133202	-7.661085	35.995246
N	-23.350009	-8.045928	35.823313
H	-24.351218	-7.923095	35.823367
C	-22.418610	-7.045724	36.002258
C	-19.995647	-6.849785	36.173171
H	-18.997339	-7.278806	36.182162
C	-22.587581	-5.666577	36.175065
H	-23.577429	-5.217545	36.179348
C	-20.159318	-5.480015	36.349203
H	-19.281288	-4.857436	36.494432
C	-21.443317	-4.894239	36.347312
H	-21.541356	-3.820985	36.488028
H	-10.291607	-8.143903	38.571968
H	-8.937756	-9.260066	38.841090
H	-10.322133	-9.264070	39.945367
C	-10.723512	-10.212235	38.015364
H	-11.809252	-10.060894	38.071547
H	-10.440964	-10.049665	36.967269
C	-10.388899	-11.635509	38.412235
C	-11.062103	-12.269990	39.468472
H	-11.854274	-11.737818	39.991127
C	-9.378537	-12.355037	37.763819
H	-8.839056	-11.896238	36.937537
C	-10.748592	-13.566646	39.870405
H	-11.290134	-14.040599	40.685198
C	-9.046356	-13.655883	38.149914
H	-8.259640	-14.198110	37.627984
C	-9.732038	-14.263073	39.206051
O	-9.454318	-15.539265	39.628336
H	-8.729134	-15.895513	39.090762
H	-15.723771	-13.913505	40.687041
H	-14.112855	-14.149805	39.957931
H	-14.417791	-12.742383	40.977367
C	-14.142060	-14.566962	42.057172
O	-12.943048	-14.601370	42.358238
N	-15.085862	-15.215221	42.780868
H	-16.059292	-15.095082	42.540623

H	-15.581474	-16.614568	44.238366
H	-14.588284	-15.266550	44.841555
H	-13.847734	-16.526281	43.835622
H	-10.768379	-2.985561	33.022567
H	-11.570071	-2.615834	34.562432
H	-12.520204	-2.708197	33.071821
C	-11.906340	-4.656733	33.853762
H	-11.030702	-5.073728	34.366816
H	-11.967047	-5.165383	32.882495
C	-13.152871	-4.962376	34.661186
C	-13.092098	-5.183962	36.042754
H	-12.129341	-5.153243	36.548488
C	-14.415915	-5.019334	34.051103
H	-14.498384	-4.858988	32.977858
C	-14.238170	-5.454499	36.794083
H	-14.161349	-5.630349	37.864810
C	-15.570620	-5.287026	34.782622
H	-16.542343	-5.337241	34.300138
C	-15.484343	-5.508891	36.162275
O	-16.649524	-5.777900	36.831000
H	-16.463028	-5.934399	37.771790
H	-11.548467	-5.624907	44.902783
H	-10.607160	-6.259179	43.543415
C	-11.969849	-7.665758	44.301736
O	-11.604596	-8.588010	43.559154
C	-12.578465	-5.523309	42.985560
H	-13.070382	-6.282292	42.364244
H	-11.987644	-4.900552	42.301795
C	-13.647650	-4.639024	43.659207
H	-14.278353	-4.193209	42.880109
H	-13.152165	-3.829259	44.208814
C	-14.522316	-5.464647	44.626572
O	-15.475278	-6.109279	44.117275
O	-14.152935	-5.491731	45.841630
N	-12.685816	-7.864663	45.436670
H	-13.167995	-7.041791	45.823366
H	-13.743217	-9.072840	46.724503
C	-12.151098	-10.189710	45.896596
O	-11.150562	-9.916622	46.562065
C	-14.425367	-9.458056	44.728042
H	-14.902700	-8.493271	44.522349
H	-14.004829	-9.825511	43.784810
O	-15.382151	-10.398454	45.199882
H	-16.107952	-9.863479	45.614563
N	-12.338762	-11.393991	45.305399
H	-13.167951	-11.537725	44.745930
H	-10.414395	-11.902152	45.748275
H	-10.909450	-12.332127	44.106483
C	-11.566195	-13.736866	45.612668
H	-10.676964	-14.374298	45.536344
H	-12.343265	-14.178678	44.980718
H	-11.907830	-13.743549	46.653851
H	-19.538631	-17.923554	47.970858
H	-20.741315	-16.796677	47.324933
H	-21.215346	-18.443656	47.769855
C	-20.885090	-17.108256	49.479174
H	-20.154847	-16.332352	49.748082
H	-20.730985	-17.931758	50.193336
C	-22.301297	-16.532825	49.680276
H	-22.442609	-15.733985	48.935942

C	-23.399425	-17.584077	49.457731
H	-24.394791	-17.148768	49.608974
H	-23.290264	-18.417399	50.165419
H	-23.372218	-18.000349	48.444956
C	-22.433908	-15.903428	51.075127
H	-23.424604	-15.454916	51.217454
H	-21.684102	-15.118647	51.231632
H	-22.295171	-16.658890	51.860463
H	-16.374022	-21.225260	47.106715
H	-14.692910	-21.660871	46.746870
H	-15.559227	-20.526022	45.697320
C	-14.927758	-19.658593	47.596983
H	-14.800882	-20.022261	48.628065
C	-13.564674	-19.147729	47.108669
H	-12.812027	-19.945668	47.119313
H	-13.196463	-18.328953	47.738345
H	-13.635146	-18.770133	46.079845
C	-15.964750	-18.526669	47.623238
H	-16.931240	-18.879458	48.004033
H	-16.130902	-18.126430	46.614165
H	-15.634729	-17.696924	48.259548
H	-22.618000	-6.697257	49.918743
H	-22.844224	-5.527527	48.606312
H	-23.022353	-5.012693	50.289011
C	-20.948592	-5.302994	49.661931
H	-20.392338	-5.968003	48.992556
H	-20.556360	-5.488960	50.672425
C	-20.654723	-3.850493	49.262133
H	-21.047794	-3.161382	50.019869
H	-21.181012	-3.632648	48.320669
C	-19.146808	-3.558593	49.042186
O	-18.557275	-4.268155	48.161086
O	-18.627265	-2.645634	49.721141
H	-26.941694	-12.977544	47.138343
H	-25.994653	-13.281526	45.669011
H	-25.849789	-11.728895	46.506966
C	-24.826055	-13.376574	47.514692
H	-24.997151	-14.449224	47.672408
H	-24.852593	-12.905893	48.505489
C	-23.459717	-13.167603	46.892623
C	-22.693334	-12.033477	47.198091
H	-23.073563	-11.311553	47.918167
C	-22.944576	-14.085645	45.964827
H	-23.523630	-14.972407	45.714176
C	-21.451573	-11.818578	46.595372
H	-20.872310	-10.933259	46.845730
C	-21.703940	-13.876239	45.358395
H	-21.325105	-14.594823	44.636913
C	-20.952742	-12.740453	45.672197
H	-19.988161	-12.578247	45.200614
H	-29.163834	-16.397361	45.219079
H	-27.907030	-16.836629	44.045173
H	-29.405876	-17.773636	44.123979
C	-29.628926	-15.826212	43.156437
H	-29.214838	-14.817420	43.286094
H	-30.704650	-15.747019	43.364175
C	-29.419796	-16.280767	41.725947
C	-28.297815	-15.877645	40.987025
H	-27.577293	-15.199788	41.443428
C	-30.324643	-17.147334	41.094613

H	-31.211407	-17.477216	41.634872
C	-28.076135	-16.312240	39.680821
H	-27.206546	-15.983739	39.119680
C	-30.122751	-17.593598	39.790155
H	-30.836644	-18.260165	39.312741
C	-28.992576	-17.183262	39.057288
O	-28.842776	-17.631767	37.798906
H	-27.979035	-17.258588	37.371896
H	-21.970887	-5.109734	45.682360
H	-20.525304	-5.302196	44.669056
H	-20.359122	-4.762252	46.349625
C	-20.801101	-6.891933	46.145946
H	-21.348378	-7.547964	45.458012
H	-21.301751	-6.991515	47.119568
C	-19.390579	-7.401050	46.242993
N	-18.373914	-6.698674	46.863412
H	-18.472045	-5.782785	47.351746
C	-18.800183	-8.567696	45.807031
H	-19.260029	-9.379785	45.258327
C	-17.244000	-7.442491	46.768144
H	-16.282220	-7.077201	47.100339
N	-17.457900	-8.591190	46.140007
N	-18.582499	-11.883650	42.022766
C	-18.949388	-10.792527	41.261184
C	-19.976265	-11.031718	40.319018
S	-20.490877	-12.702109	40.432625
C	-19.290024	-12.918512	41.699865
C	-18.380220	-9.498063	41.357361
C	-18.870762	-8.512813	40.470304
C	-19.891286	-8.765245	39.536082
H	-20.238421	-7.979311	38.872838
C	-20.458791	-10.029856	39.459042
H	-21.249657	-10.231215	38.744789
C	-17.363222	-8.999846	42.238652
H	-16.889183	-9.595443	43.011378
C	-17.083387	-7.683485	42.026215
H	-16.391966	-7.046693	42.577827
S	-18.043795	-6.987101	40.737272
C	-19.051560	-14.267888	42.311711
F	-18.055590	-14.938325	41.662589
F	-20.150522	-15.045156	42.235203
F	-18.683770	-14.178478	43.599337

Optimized geomtry of complex of compound 15 and the active site of BChE

scf done: -4905.780538

C	-23.474014	-17.456998	35.214014
C	-19.478984	-10.102998	34.397006
C	-10.028065	-9.158031	38.895111
C	-14.648190	-13.805081	40.847882
C	-14.756787	-15.940957	43.993040
C	-11.677014	-3.153012	33.613000
C	-11.608914	-6.215998	43.983082
C	-13.280143	-9.172032	45.734910
C	-11.232937	-12.314897	45.157010
C	-20.582035	-17.596945	48.058034
C	-15.416029	-20.836040	46.740965
C	-22.438986	-5.657014	49.617946
C	-25.970752	-12.807793	46.657064

C	-28.990149	-16.762195	44.198886
C	-20.921000	-5.425010	45.684047
H	-22.852421	-16.747437	34.653182
H	-23.461714	-18.417641	34.687629
H	-23.012734	-17.622829	36.193078
C	-24.904409	-16.945110	35.373320
H	-25.368752	-16.765675	34.393763
H	-24.913396	-15.970742	35.881653
C	-25.833286	-17.889644	36.168037
O	-27.041998	-17.467454	36.274287
O	-25.372537	-18.947109	36.637774
H	-18.855476	-10.368353	33.536473
H	-20.374654	-9.591257	34.025634
H	-19.801266	-11.034305	34.877503
C	-18.705017	-9.213300	35.378998
H	-17.793484	-9.737657	35.704116
H	-18.345866	-8.317495	34.851392
C	-19.503547	-8.787202	36.580340
C	-20.762708	-9.203491	36.940262
H	-21.422674	-9.909543	36.456441
C	-19.091343	-7.834987	37.585349
N	-21.162106	-8.564884	38.102086
H	-22.033607	-8.723753	38.583661
C	-20.157401	-7.719974	38.522866
C	-17.926103	-7.072917	37.788340
H	-17.080744	-7.157167	37.110052
C	-20.091926	-6.866748	39.631007
H	-20.916124	-6.796677	40.336315
C	-17.856392	-6.220561	38.884787
H	-16.946386	-5.658808	39.063197
C	-18.929750	-6.118835	39.796022
H	-18.836962	-5.456944	40.652826
H	-9.483206	-8.707284	39.732581
H	-9.298496	-9.577525	38.192257
H	-10.626423	-9.987753	39.283937
C	-10.930775	-8.125072	38.195330
H	-11.571436	-7.634066	38.938536
H	-10.309447	-7.331675	37.761529
C	-11.806415	-8.726562	37.115470
C	-13.020712	-9.350248	37.441544
H	-13.328217	-9.409865	38.480710
C	-11.454320	-8.656605	35.764071
H	-10.525384	-8.168229	35.477794
C	-13.865610	-9.861165	36.459851
H	-14.814060	-10.322002	36.720487
C	-12.286883	-9.165587	34.764257
H	-11.999611	-9.086249	33.717336
C	-13.502893	-9.763006	35.111978
O	-14.376980	-10.269765	34.182825
H	-14.016173	-10.118913	33.294812
H	-15.534520	-13.233629	41.113788
H	-14.890981	-14.413518	39.966746
H	-13.839805	-13.133532	40.561552
C	-14.175972	-14.766071	41.922415
O	-13.122314	-15.400944	41.816161
N	-15.015466	-14.935147	42.975920
H	-15.787483	-14.292530	43.093029
H	-15.644820	-16.046245	44.620186
H	-13.904006	-15.672671	44.626371
H	-14.537580	-16.901148	43.516749

H	-10.934120	-2.691317	32.951324
H	-12.528290	-2.468154	33.703707
H	-12.037420	-4.066369	33.126607
C	-11.072000	-3.458504	34.994756
H	-10.686129	-2.529116	35.432873
H	-10.198160	-4.112439	34.854858
C	-12.039087	-4.106612	35.967669
C	-12.330568	-3.534999	37.212968
H	-11.875644	-2.582990	37.482057
C	-12.658300	-5.329485	35.661425
H	-12.449744	-5.817108	34.711511
C	-13.183856	-4.156732	38.129729
H	-13.399056	-3.696990	39.090058
C	-13.514838	-5.959926	36.556046
H	-13.961591	-6.919067	36.316282
C	-13.772280	-5.393515	37.816925
O	-14.577198	-6.073201	38.658769
H	-14.615745	-5.687430	39.604749
H	-12.325406	-5.537781	44.449451
H	-10.625782	-6.021371	44.435381
C	-11.927903	-7.662837	44.328669
O	-11.425339	-8.604008	43.709473
C	-11.535828	-5.977231	42.455897
H	-11.510342	-6.952178	41.958392
H	-10.583241	-5.490253	42.211111
C	-12.664708	-5.123125	41.859179
H	-12.486341	-5.001737	40.784923
H	-12.623241	-4.111778	42.293264
C	-14.106379	-5.635596	42.078110
O	-14.388167	-6.160238	43.175379
O	-14.930610	-5.429261	41.116265
N	-12.711671	-7.851117	45.428021
H	-13.296996	-7.065067	45.681648
H	-13.668699	-9.105722	46.760413
C	-12.144377	-10.183503	45.834568
O	-11.162349	-9.943760	46.539844
C	-14.477950	-9.379061	44.803202
H	-15.058381	-8.449785	44.812732
H	-14.104021	-9.494669	43.787312
O	-15.292805	-10.476455	45.186695
H	-16.197205	-10.094131	45.306980
N	-12.320886	-11.361754	45.200091
H	-13.098337	-11.461676	44.562048
H	-10.724655	-12.267128	46.123252
H	-10.494753	-12.022264	44.398260
C	-11.737384	-13.733295	44.894788
H	-10.901876	-14.441447	44.914693
H	-12.223756	-13.831985	43.917939
H	-12.459249	-14.030236	45.664197
H	-20.024950	-18.532773	48.186966
H	-21.156089	-17.675870	47.125877
H	-21.298013	-17.521628	48.883644
C	-19.631121	-16.394507	48.016426
H	-18.854736	-16.579499	47.259295
H	-19.103482	-16.311900	48.979030
C	-20.305025	-15.043894	47.704367
H	-20.778042	-15.124316	46.712953
C	-21.400369	-14.687417	48.720573
H	-21.818061	-13.698643	48.504759
H	-20.989624	-14.668047	49.739465

H	-22.226909	-15.406437	48.704602
C	-19.258247	-13.921867	47.639391
H	-19.707650	-12.980457	47.302460
H	-18.440468	-14.175798	46.953827
H	-18.813812	-13.747770	48.628528
H	-15.746862	-21.607719	46.035315
H	-15.211113	-21.320945	47.703331
H	-14.470464	-20.422398	46.365974
C	-16.469530	-19.726010	46.888539
H	-17.395995	-20.185618	47.265486
C	-16.020398	-18.668079	47.906169
H	-15.817931	-19.116655	48.886489
H	-16.785887	-17.893504	48.040138
H	-15.100177	-18.172788	47.568311
C	-16.782005	-19.077963	45.532730
H	-17.140286	-19.817872	44.806800
H	-15.883459	-18.606528	45.112834
H	-17.551271	-18.301593	45.631464
H	-22.818423	-6.676845	49.760562
H	-22.891852	-5.257796	48.700824
H	-22.798671	-5.045512	50.455602
C	-20.910691	-5.626870	49.525980
H	-20.575459	-6.254113	48.693005
H	-20.476345	-6.070126	50.433693
C	-20.358173	-4.211279	49.339147
H	-20.634488	-3.579198	50.191972
H	-20.827403	-3.762927	48.449403
C	-18.823442	-4.141160	49.147314
O	-18.307783	-4.979903	48.335590
O	-18.204354	-3.255145	49.774807
H	-26.785694	-13.261221	47.233527
H	-26.028543	-13.190475	45.631695
H	-26.138216	-11.725362	46.618685
C	-24.603334	-13.125200	47.287575
H	-24.475174	-14.214845	47.339786
H	-24.582273	-12.760046	48.321644
C	-23.457445	-12.517952	46.507122
C	-22.718088	-11.437892	47.007847
H	-22.955453	-11.041650	47.993123
C	-23.137011	-13.013739	45.234109
H	-23.700892	-13.852572	44.830766
C	-21.679167	-10.869760	46.263236
H	-21.105774	-10.037239	46.663550
C	-22.112234	-12.440752	44.482826
H	-21.884390	-12.824873	43.492356
C	-21.374811	-11.370217	44.996381
H	-20.569058	-10.946854	44.408338
H	-28.879357	-16.162396	45.109187
H	-28.554813	-17.749858	44.391366
H	-30.062609	-16.900432	44.017943
C	-28.305679	-16.071495	43.015731
H	-27.253076	-15.886214	43.275850
H	-28.748869	-15.072436	42.887761
C	-28.361203	-16.812941	41.688875
C	-27.692243	-16.281267	40.573720
H	-27.141304	-15.347638	40.685185
C	-29.056097	-18.014376	41.500263
H	-29.589835	-18.470582	42.329823
C	-27.708237	-16.904687	39.329891
H	-27.189484	-16.475524	38.478632

```
 C   -29.082190   -18.658013    40.261104
 H   -29.622323   -19.592702    40.132939
 C   -28.408820   -18.117536    39.152721
 O   -28.463704   -18.768866    37.977882
 H   -27.864465   -18.299791    37.277900
 H   -21.955279    -5.069680    45.749279
 H   -20.496800    -5.088085    44.731396
 H   -20.342161    -4.961775    46.489971
 C   -20.870427    -6.964060    45.792802
 H   -21.396124    -7.419216    44.945054
 H   -21.428313    -7.267849    46.691199
 C   -19.490332    -7.557480    45.851144
 N   -18.535536    -7.152928    46.765061
 H   -18.590140    -6.333162    47.416882
 C   -18.904848    -8.599262    45.159287
 H   -19.317016    -9.172457    44.341459
 C   -17.446391    -7.941587    46.591774
 H   -16.544706    -7.823475    47.177345
 N   -17.627519    -8.840721    45.631994
 C   -14.279665    -8.717234    40.929349
 H   -14.322985    -7.741093    40.463135
 C   -15.492273    -9.313817    41.365034
 C   -15.408383   -10.597949    41.992510
 C   -14.147473   -11.210910    42.188686
 H   -14.112498   -12.153809    42.722678
 C   -12.987346   -10.630391    41.722068
 C   -13.074852    -9.358760    41.095891
 H   -12.159832    -8.879481    40.767306
 C   -16.646322   -11.131489    42.389906
 C   -17.870741   -10.516597    42.188295
 C   -17.936406    -9.234174    41.593548
 H   -18.884861    -8.727629    41.451643
 C   -16.749899    -8.654623    41.202442
 H   -16.744152    -7.659976    40.772412
 N   -18.882919   -11.361760    42.661502
 C   -18.261921   -12.407277    43.112522
 O   -16.890336   -12.351403    42.988169
 C   -11.643556   -11.283935    41.897650
 H   -11.736599   -12.309762    42.265181
 H   -11.045119   -10.703002    42.605714
 H   -11.095448   -11.322446    40.949400
 C   -18.816853   -13.626499    43.757984
 H   -19.811792   -13.839277    43.364758
 H   -18.905759   -13.484956    44.840267
 H   -18.169079   -14.488191    43.574334
```

Optimized geometry of complex of trans-8 with the BChE active site
(starting ligand's geometry from cluster of 11 poses obtained by docking)

```
scf done: -5265.578177
 C   -23.473396   -17.461234    35.206725
 C   -19.478996   -10.102991    34.397006
 C   -10.028031    -9.157979    38.895006
 C   -14.648000   -13.805002    40.848001
 C   -14.757012   -15.941033    43.993019
 C   -11.677001    -3.153008    33.613004
 C   -11.608992    -6.216008    43.983002
 C   -13.280021    -9.172025    45.734987
 C   -11.232998   -12.314982    45.157009
 C   -20.582068   -17.597050    48.057520
```

C	-15.415992	-20.835962	46.740978
C	-22.438905	-5.657141	49.617831
C	-25.969807	-12.798756	46.671023
C	-28.991777	-16.767038	44.192812
C	-20.921003	-5.424793	45.684078
H	-22.771231	-16.690374	34.864461
H	-23.718247	-18.105888	34.356252
H	-22.959283	-18.085451	35.945981
C	-24.736770	-16.839485	35.799401
H	-25.225586	-16.186617	35.061774
H	-24.487903	-16.183885	36.644584
C	-25.792048	-17.858222	36.288056
O	-26.792972	-17.322682	36.888841
O	-25.618418	-19.072236	36.069116
H	-19.254035	-11.166476	34.540475
H	-18.540276	-9.544711	34.482810
H	-19.854557	-9.968037	33.376080
C	-20.513165	-9.615977	35.428772
H	-21.430491	-10.209535	35.326179
H	-20.129109	-9.820343	36.438039
C	-20.851454	-8.156084	35.303175
C	-22.013851	-7.619913	34.805363
H	-22.898883	-8.120546	34.435404
C	-20.012247	-7.034307	35.658374
N	-21.950428	-6.237499	34.822378
H	-22.691698	-5.617297	34.534544
C	-20.735519	-5.847621	35.346475
C	-18.727712	-6.913472	36.221232
H	-18.147764	-7.796532	36.477352
C	-20.212331	-4.569607	35.578529
H	-20.781408	-3.675872	35.335063
C	-18.203800	-5.647501	36.455052
H	-17.215836	-5.555362	36.894954
C	-18.939836	-4.486270	36.135065
H	-18.504701	-3.509736	36.330813
H	-10.825157	-9.316275	39.630377
H	-9.629065	-8.147919	39.042277
H	-9.222032	-9.870438	39.107103
C	-10.558561	-9.334681	37.460711
H	-10.978652	-10.343448	37.356078
H	-11.385343	-8.633161	37.295525
C	-9.492836	-9.117487	36.406136
C	-8.646175	-10.160859	36.001855
H	-8.781327	-11.152679	36.428643
C	-9.292300	-7.857520	35.827527
H	-9.938505	-7.029506	36.110010
C	-7.636098	-9.962503	35.062174
H	-6.988682	-10.777821	34.752394
C	-8.285336	-7.639803	34.885113
H	-8.152153	-6.652373	34.446696
C	-7.452118	-8.694670	34.500707
O	-6.446155	-8.547153	33.578138
H	-6.432922	-7.624965	33.275867
H	-14.840234	-12.883678	41.405023
H	-15.465535	-13.963164	40.136589
H	-13.729269	-13.682142	40.266521
C	-14.515129	-15.033613	41.726715
O	-14.205500	-16.135482	41.270584
N	-14.753342	-14.836964	43.051520
H	-15.099942	-13.939852	43.360375

H	-14.641009	-15.550043	45.006208
H	-13.924423	-16.610554	43.766590
H	-15.687381	-16.522169	43.942079
H	-10.905926	-2.660461	33.008080
H	-12.160901	-2.391589	34.235299
H	-12.434992	-3.557150	32.931980
C	-11.066697	-4.267143	34.484915
H	-10.293123	-3.831921	35.130094
H	-10.555007	-4.986066	33.829705
C	-12.083118	-4.996049	35.343214
C	-12.212041	-4.735123	36.713993
H	-11.557373	-4.004973	37.185134
C	-12.953478	-5.945190	34.782728
H	-12.875650	-6.183368	33.723171
C	-13.168899	-5.377780	37.505022
H	-13.224672	-5.157839	38.568498
C	-13.922937	-6.586297	35.548850
H	-14.601335	-7.308502	35.103211
C	-14.046622	-6.298990	36.917549
O	-15.031167	-6.944891	37.603213
H	-15.159872	-6.585864	38.518687
H	-11.697197	-5.608527	44.892650
H	-10.559221	-6.537176	43.923090
C	-12.407891	-7.510387	44.121485
O	-12.595144	-8.262406	43.162473
C	-11.954305	-5.368850	42.748548
H	-11.941891	-6.000796	41.855663
H	-11.155554	-4.628177	42.616833
C	-13.301213	-4.643663	42.862146
H	-13.395848	-4.160996	43.842188
H	-14.119995	-5.375422	42.795327
C	-13.550840	-3.563892	41.789788
O	-14.167576	-2.535414	42.119595
O	-13.113595	-3.829122	40.606755
N	-12.877676	-7.803042	45.375779
H	-12.502885	-7.244403	46.130889
H	-13.601350	-9.137696	46.781789
C	-12.044297	-10.077301	45.635649
O	-10.903620	-9.614812	45.678783
C	-14.515850	-9.621829	44.908069
H	-15.096022	-8.724491	44.670341
H	-14.187478	-10.064834	43.963718
O	-15.315493	-10.557500	45.617416
H	-16.129628	-10.061836	45.890794
N	-12.301165	-11.400145	45.512488
H	-13.265881	-11.704373	45.449015
H	-11.368905	-13.250700	45.712984
H	-10.303299	-11.857324	45.498203
C	-11.173003	-12.601171	43.652887
H	-10.342829	-13.280417	43.425908
H	-11.025070	-11.673154	43.090578
H	-12.099812	-13.071708	43.304281
H	-20.000903	-18.509898	47.880642
H	-21.030324	-17.299187	47.101257
H	-21.399096	-17.856017	48.740361
C	-19.692691	-16.481798	48.620158
H	-18.840011	-16.335695	47.942180
H	-19.264027	-16.803371	49.581670
C	-20.395745	-15.124009	48.817999
H	-20.849237	-14.840294	47.855397

C	-21.518847	-15.190787	49.864292
H	-22.006242	-14.215105	49.974670
H	-21.118914	-15.478910	50.846236
H	-22.292503	-15.917534	49.592652
C	-19.377364	-14.036520	49.191581
H	-19.858620	-13.055801	49.281644
H	-18.586784	-13.953564	48.435940
H	-18.896904	-14.263454	50.152965
H	-16.384296	-21.161941	47.139786
H	-14.707346	-21.665060	46.857597
H	-15.542835	-20.654768	45.665373
C	-14.916792	-19.569892	47.451884
H	-14.806659	-19.805701	48.521311
C	-13.540307	-19.144089	46.921483
H	-12.801555	-19.946443	47.038496
H	-13.164452	-18.260615	47.451589
H	-13.594005	-18.894288	45.853396
C	-15.935239	-18.427725	47.326006
H	-16.910424	-18.714996	47.737860
H	-16.086943	-18.154007	46.273150
H	-15.596725	-17.530540	47.858175
H	-22.891323	-6.644556	49.775051
H	-22.640889	-5.359365	48.581076
H	-22.960400	-4.944866	50.270378
C	-20.934779	-5.673252	49.902285
H	-20.446056	-6.410750	49.254960
H	-20.759234	-6.010423	50.934182
C	-20.261531	-4.308706	49.696583
H	-20.652906	-3.582153	50.418904
H	-20.512723	-3.937597	48.691543
C	-18.719663	-4.371150	49.819978
O	-18.122327	-5.144618	48.996876
O	-18.170850	-3.677821	50.702432
H	-26.717217	-13.578425	46.483210
H	-25.682633	-12.364641	45.706758
H	-26.443343	-12.008916	47.265406
C	-24.744535	-13.378413	47.400948
H	-24.316656	-14.186395	46.794417
H	-25.071372	-13.833837	48.344272
C	-23.678264	-12.339410	47.683668
C	-23.687107	-11.603998	48.878203
H	-24.455523	-11.805693	49.622054
C	-22.675160	-12.063256	46.742595
H	-22.649408	-12.625003	45.810950
C	-22.725054	-10.621900	49.126574
H	-22.748492	-10.066229	50.060687
C	-21.711138	-11.082175	46.985148
H	-20.939406	-10.882256	46.246580
C	-21.732629	-10.356930	48.179423
H	-20.980106	-9.595974	48.367475
H	-28.930019	-16.626962	45.279297
H	-30.050097	-16.859571	43.921243
H	-28.604399	-15.861343	43.711832
C	-28.199466	-18.008599	43.744523
H	-28.592222	-18.890219	44.268339
H	-27.155885	-17.896986	44.069004
C	-28.246778	-18.245227	42.248701
C	-27.389781	-17.557591	41.376296
H	-26.657074	-16.864338	41.788169
C	-29.168761	-19.131818	41.673855

```
 H   -29.847427   -19.687328    42.320219
 C   -27.441880   -17.735468    39.995285
 H   -26.772458   -17.193838    39.334198
 C   -29.236484   -19.326446    40.295169
 H   -29.952253   -20.022869    39.865590
 C   -28.372561   -18.630645    39.428565
 O   -28.469004   -18.850324    38.106013
 H   -27.769943   -18.292882    37.586676
 H   -21.974320    -5.135603    45.601140
 H   -20.425933    -5.198325    44.732928
 H   -20.458480    -4.803625    46.458534
 C   -20.807843    -6.925982    46.025327
 H   -21.293386    -7.512902    45.236574
 H   -21.386819    -7.118254    46.939980
 C   -19.417124    -7.476577    46.198226
 N   -18.501559    -7.004936    47.120122
 H   -18.570179    -6.202021    47.800545
 C   -18.793634    -8.556393    45.602406
 H   -19.185042    -9.199600    44.824307
 C   -17.403420    -7.793234    47.042814
 H   -16.534464    -7.627676    47.664843
 N   -17.532658    -8.753839    46.136670
 C   -16.137143    -7.599562    40.896545
 N   -16.003034    -6.412264    40.201093
 C   -16.949391    -5.543795    40.496818
 S   -18.110641    -6.166961    41.668735
 C   -17.219346    -7.654915    41.730941
 H   -17.533169    -8.468884    42.369163
 C   -15.120925    -8.686179    40.693473
 H   -14.177442    -8.433977    41.192239
 H   -14.915332    -8.821188    39.626141
 H   -15.476653    -9.634821    41.106183
 C   -17.070367    -4.191244    39.992765
 H   -18.033615    -3.709846    40.140595
 C   -16.027571    -3.528579    39.436215
 H   -15.070795    -4.037736    39.433529
 C   -16.036794    -2.182602    38.882053
 C   -14.804061    -1.505571    38.632770
 C   -14.842729    -0.227256    38.035002
 H   -13.892775     0.267065    37.852182
 C   -16.044057     0.382762    37.702555
 H   -16.037553     1.368937    37.243859
 C   -17.260508    -0.267790    37.957909
 H   -18.205120     0.200697    37.696162
 C   -17.240416    -1.529661    38.535681
 H   -18.179315    -2.050287    38.704533
 O   -13.591184    -2.002173    38.895742
 H   -13.513871    -2.733009    39.636795
```

Optimized geometry of complex of trans-8 with the BChE active site
(starting ligand's geometry from cluster of 8 poses obtained by docking)

```
scf done: -5265.587355
 C   -23.474008   -17.457002    35.214000
 C   -19.479000   -10.102999    34.396998
 C   -10.027999    -9.158001    38.894999
 C   -14.648015   -13.805001    40.848013
 C   -14.756974   -15.941003    43.992989
 C   -11.677000    -3.153000    33.613000
 C   -11.609016    -6.216000    43.983004
```

C	-13.280011	-9.172041	45.735014
C	-11.233001	-12.315001	45.156998
C	-20.582008	-17.596911	48.057981
C	-15.416005	-20.835992	46.741001
C	-22.439004	-5.656997	49.618003
C	-25.970991	-12.808080	46.656992
C	-28.989982	-16.761970	44.199011
C	-20.920989	-5.425003	45.683998
H	-22.870359	-16.642876	34.793252
H	-23.634702	-18.207214	34.433114
H	-22.889381	-17.937015	36.006847
C	-24.807366	-16.940401	35.752344
H	-25.362410	-16.418124	34.959179
H	-24.646879	-16.188467	36.535975
C	-25.740704	-18.031655	36.325595
O	-26.775648	-17.567688	36.928483
O	-25.452635	-19.232301	36.159505
H	-19.193611	-10.858890	35.137744
H	-18.761216	-9.278866	34.469324
H	-19.379007	-10.546796	33.399327
C	-20.919284	-9.614882	34.639636
H	-21.603166	-10.471267	34.575017
H	-21.002075	-9.232944	35.665979
C	-21.373925	-8.554534	33.673619
C	-22.303018	-8.700321	32.672889
H	-22.881022	-9.571506	32.394127
C	-20.920681	-7.182865	33.600756
N	-22.455661	-7.511236	31.979469
H	-23.094013	-7.361979	31.213278
C	-21.623782	-6.559541	32.529193
C	-19.998884	-6.417278	34.339634
H	-19.438012	-6.853498	35.161284
C	-21.431542	-5.214923	32.188306
H	-21.979306	-4.758333	31.367607
C	-19.807382	-5.080651	34.005845
H	-19.099884	-4.488668	34.579335
C	-20.516051	-4.485451	32.940174
H	-20.344824	-3.438529	32.702758
H	-10.430060	-9.165470	39.914757
H	-9.060195	-9.673412	38.905293
H	-10.708027	-9.736202	38.258820
C	-9.881255	-7.716202	38.376234
H	-10.859774	-7.222267	38.418238
H	-9.222701	-7.157419	39.053275
C	-9.324157	-7.654625	36.968994
C	-10.156502	-7.816417	35.850002
H	-11.226803	-7.944439	35.994369
C	-7.954141	-7.481028	36.736687
H	-7.281566	-7.348452	37.581603
C	-9.647180	-7.810595	34.552524
H	-10.300323	-7.936437	33.694119
C	-7.425213	-7.470552	35.444112
H	-6.356779	-7.329621	35.290331
C	-8.273939	-7.637784	34.346209
O	-7.820039	-7.637716	33.051052
H	-6.858272	-7.508225	33.056694
H	-14.894664	-12.889939	41.393184
H	-15.443706	-14.002526	40.121886
H	-13.724482	-13.644437	40.283797
C	-14.477636	-15.020560	41.737805

O	-14.080596	-16.103451	41.303749
N	-14.790121	-14.839260	43.049198
H	-15.194645	-13.961890	43.343952
H	-14.804381	-15.542402	45.008260
H	-13.827734	-16.502873	43.870729
H	-15.596231	-16.632708	43.843265
H	-10.761042	-2.924644	33.053645
H	-11.501230	-2.905356	34.666316
H	-12.472817	-2.494139	33.245757
C	-12.071729	-4.631667	33.456022
H	-11.239814	-5.259914	33.799811
H	-12.208445	-4.853296	32.389198
C	-13.331901	-4.992016	34.214585
C	-13.288802	-5.340222	35.572298
H	-12.326810	-5.383953	36.080323
C	-14.591147	-4.951142	33.599413
H	-14.661130	-4.690259	32.544300
C	-14.443549	-5.637156	36.296290
H	-14.380174	-5.904815	37.347193
C	-15.757731	-5.245807	34.302156
H	-16.726855	-5.225524	33.811370
C	-15.697439	-5.593725	35.661392
O	-16.858423	-5.867352	36.302291
H	-16.682116	-6.242906	37.217883
H	-12.084113	-5.521398	44.686146
H	-10.523832	-6.104488	44.099086
C	-11.958420	-7.662108	44.303822
O	-11.584037	-8.592979	43.586062
C	-12.002396	-5.887697	42.528497
H	-11.538888	-6.629483	41.872366
H	-11.590654	-4.911597	42.247909
C	-13.519275	-5.901492	42.315618
H	-13.989550	-4.994428	42.716693
H	-13.981479	-6.731274	42.866244
C	-13.966223	-6.069010	40.868979
O	-15.287686	-6.101075	40.780983
O	-13.197609	-6.188238	39.921650
N	-12.724891	-7.860877	45.406482
H	-13.057606	-7.060157	45.924037
H	-13.722346	-9.078584	46.733971
C	-12.125363	-10.171946	45.867505
O	-11.089726	-9.862076	46.456036
C	-14.439346	-9.505112	44.760071
H	-14.988621	-8.572017	44.588012
H	-14.028602	-9.825670	43.794962
O	-15.310627	-10.501863	45.263073
H	-16.109671	-10.013933	45.590708
N	-12.339939	-11.398451	45.340379
H	-13.202306	-11.575111	44.842586
H	-10.523354	-12.124436	45.964669
H	-10.713960	-12.089937	44.213231
C	-11.692689	-13.771243	45.167969
H	-10.837674	-14.438765	45.015285
H	-12.415997	-13.967539	44.367600
H	-12.163446	-14.026432	46.123952
H	-20.064931	-18.546503	47.876432
H	-21.076713	-17.304954	47.123015
H	-21.364054	-17.786433	48.801728
C	-19.597470	-16.516207	48.520665
H	-18.786835	-16.438778	47.782472

H	-19.121676	-16.831130	49.462064
C	-20.212278	-15.116064	48.717621
H	-20.715694	-14.837243	47.778911
C	-21.262916	-15.087967	49.838404
H	-21.690867	-14.084390	49.945191
H	-20.812278	-15.364914	50.801443
H	-22.090165	-15.779989	49.645475
C	-19.114843	-14.075057	48.984081
H	-19.537040	-13.067201	49.070542
H	-18.374533	-14.059651	48.175064
H	-18.582533	-14.296969	49.918981
H	-16.401298	-21.087596	47.151873
H	-14.797122	-21.741104	46.771715
H	-15.554882	-20.562807	45.686585
C	-14.762857	-19.685803	47.520687
H	-14.647013	-20.010931	48.565858
C	-13.364927	-19.368646	46.970477
H	-12.713676	-20.250743	47.002246
H	-12.880559	-18.571516	47.547349
H	-13.423383	-19.035338	45.925749
C	-15.655323	-18.436428	47.514517
H	-16.644037	-18.649043	47.939097
H	-15.805354	-18.069714	46.490259
H	-15.207041	-17.622791	48.097399
H	-22.857167	-6.657287	49.787725
H	-22.701095	-5.354951	48.596040
H	-22.944094	-4.964759	50.304138
C	-20.921403	-5.636568	49.824471
H	-20.448043	-6.353253	49.143528
H	-20.684410	-5.981348	50.841486
C	-20.297136	-4.251965	49.602584
H	-20.675777	-3.543347	50.348965
H	-20.608224	-3.878128	48.615216
C	-18.749181	-4.255846	49.650611
O	-18.159115	-5.040417	48.833071
O	-18.187188	-3.497989	50.469921
H	-26.805873	-13.517984	46.630268
H	-25.722546	-12.537106	45.624682
H	-26.311949	-11.900070	47.167616
C	-24.753483	-13.418552	47.374533
H	-24.458627	-14.340328	46.857473
H	-25.043994	-13.708378	48.392194
C	-23.573255	-12.470786	47.434528
C	-23.395564	-11.607585	48.525427
H	-24.098803	-11.645966	49.355185
C	-22.651742	-12.404316	46.378723
H	-22.770870	-13.068018	45.524566
C	-22.332057	-10.702162	48.561349
H	-22.212427	-10.042681	49.417354
C	-21.587873	-11.500370	46.409067
H	-20.884413	-11.463783	45.581304
C	-21.423912	-10.643922	47.501188
H	-20.597071	-9.939422	47.518988
H	-28.939760	-16.547348	45.273900
H	-30.035714	-16.967726	43.941428
H	-28.687450	-15.857452	43.658737
C	-28.088050	-17.952524	43.825526
H	-28.398587	-18.831057	44.406568
H	-27.058919	-17.726003	44.136331
C	-28.114017	-18.288268	42.348216

C	-27.336400	-17.572023	41.425641
H	-26.680684	-16.779691	41.785243
C	-28.937287	-19.303257	41.839971
H	-29.551875	-19.884141	42.526881
C	-27.371478	-17.844528	40.059658
H	-26.764074	-17.280116	39.358883
C	-28.986291	-19.593733	40.477383
H	-29.624852	-20.388605	40.100143
C	-28.202726	-18.868441	39.560221
O	-28.277318	-19.182994	38.255509
H	-27.643535	-18.588908	37.694950
H	-21.978724	-5.143378	45.641512
H	-20.456237	-5.159012	44.727949
H	-20.441044	-4.829452	46.467666
C	-20.783302	-6.935844	45.966162
H	-21.269626	-7.501475	45.162761
H	-21.346395	-7.170780	46.880971
C	-19.380662	-7.464207	46.101819
N	-18.459819	-6.988944	47.016965
H	-18.543717	-6.186922	47.694315
C	-18.745028	-8.517382	45.474175
H	-19.132856	-9.150498	44.686554
C	-17.345707	-7.750788	46.905146
H	-16.469439	-7.580171	47.515769
N	-17.471160	-8.696506	45.982159
C	-22.914146	-7.066348	37.890386
N	-21.702814	-7.722703	37.903734
C	-20.719866	-6.947975	38.304960
S	-21.252057	-5.302356	38.706009
C	-22.876166	-5.758000	38.291089
H	-23.694171	-5.054128	38.356892
C	-24.140055	-7.807808	37.440095
H	-25.028895	-7.172429	37.492463
H	-24.311105	-8.694331	38.062353
H	-24.023791	-8.155442	36.406404
C	-19.322518	-7.303569	38.429160
H	-18.624079	-6.523423	38.703965
C	-18.876604	-8.571206	38.221604
H	-19.636180	-9.314175	37.977820
C	-17.527181	-9.097581	38.302671
C	-16.360342	-8.291817	38.550815
C	-15.111462	-8.959734	38.655163
H	-14.233869	-8.350437	38.852701
C	-14.999284	-10.335315	38.501255
H	-14.020523	-10.804724	38.581500
C	-16.132149	-11.119025	38.231382
H	-16.046085	-12.193784	38.097180
C	-17.367553	-10.493185	38.139662
H	-18.257667	-11.089180	37.943420
O	-16.432522	-6.981050	38.668784
H	-15.622510	-6.396522	39.848844
